# Does life history shape sexual size dimorphism in anurans? A comparative analysis

**DOI:** 10.1186/1471-2148-13-27

**Published:** 2013-01-31

**Authors:** Xu Han, Jinzhong Fu

**Affiliations:** 1 Department of Integrative Biology, University of Guelph, Guelph, Ontario, N1G 2W1, Canada; 2 Present address: Department of Biology, Queen’s University, Kingston, Ontario, K7L 3N6, Canada

**Keywords:** Sexual size dimorphism, Phylogenetic comparative analysis, Life history, Anuran, Male combat, Parental care, Fecundity advantage, Rensch’s rule

## Abstract

**Background:**

The evolution of sexual size dimorphism (SSD) is likely constrained by life history. Using phylogenetic comparative methods, we examined correlations between SSD among anurans and their life history traits, including egg size, clutch size, mating combat, and parental care behaviour. We used sexual dimorphism index (SDI = Body-size_female_ /Body-size_male_ –1) as the measurement for SSD. Body size, life history and phylogenetic data were collected from published literature. Data were analysed at two levels: all anuran species and within individual families.

**Results:**

Female-biased SSD is the predominant form in anurans. SSD decreases along with the body size increase, following the prediction of Rensch’s rule, but the magnitude of decrease is very small. More importantly, female body size is positively correlated with both fecundity related traits, egg size and clutch size, and SDI is also positively correlated with clutch size, suggesting fecundity advantage may have driven the evolution of female body size and consequently leads to the evolution of female-biased SSD. Furthermore, the presence of parental care, male parental care in particular, is negatively correlated with SDI, indicating that species with parental care tend to have a smaller SDI. A negative correlation between clutch size and parental care further suggests that parental care likely reduces the fecundity selection pressure on female body size. On the other hand, there is a general lack of significant correlation between SDI and the presence of male combat behaviour, which is surprising and contradictory to previous studies.

**Conclusions:**

We find clear evidence to support the ‘fecundity advantage hypothesis’ and the ‘parental care hypothesis’ in shaping SSD in anurans. Nevertheless, the relationships of both parental care and combat behaviour to the evolution of SSD are complex in anurans and the extreme diversity of life history traits may have masked some potential interesting relationships. Our study represents the most comprehensive study of SSD in anurans to date.

## Background

Sexual size dimorphism (SSD), where males and females differ in body size, is the most conspicuous difference between the two sexes in many animals. Females are larger than males in most animals, while the opposite pattern is predominant in birds and mammals [1,2]. SSD has been hypothesized as adaptation of males and females to their disparate reproductive roles and associated differences in ecology and life history strategies [2,3]. A widely accepted explanation of female-biased SSD is that selection for fecundity favors larger females, because they can produce more offspring or can have larger energy storage for reproduction (“the fecundity advantage hypothesis” [1]). In contrast, a male-biased SSD is expected when natural selection for female fecundity is less intense than sexual selection on male body size. In this scenario, large size gives advantages to males in contests for females or for female choice of larger males [1]. Selective advantages may bias SSD to one way or another; and the extent of SSD is likely constrained by the shared genomes of the two sexes [2]. A widespread SSD pattern in the animal kingdom is that the magnitude of SSD decreases with body size in groups where females are the larger sex, but increases with body size in groups that males are the larger sex (Rensch’s rule) [4].

Selection on many aspects of life history undoubtedly plays a major role in determining direction and magnitude of SSD, and studies on many animal groups have attempted to explain SSD by their life history traits. Fecundity-related life history traits, such as egg size and clutch size, are probably the most evident [1,5], but others are often controversial [2]. For example, parental care is likely associated with the body size of the care-providing parent, and it may lead to dimorphism between two sexes. In some cases, selection may favor larger parents because they provide more resources to offspring (*e.g.*[6]). In other cases, parental care activities may result in the small body size of the care-providing parents because the energy expenditure on breeding activities may constrain growth (*e.g.*[7]).

We know very little about the impact of life history traits on the evolution of SSD in anuran amphibians, despite the fact that they have a tremendous diversity of life history traits [8-10]. Shine (1979) presented the most comprehensive study to date that tested adaptive hypotheses of SSD in relation to mating-related life history traits [11]. Assessing 589 anuran species, Shine concluded that female-biased SSD is the common pattern in anurans with only approximately 10% species showing male-biased SSD. Furthermore, male-biased SSD was strongly correlated with the presence of male combat behaviour and the appearance of male weaponry, such as spines and tusks [11]. Several recent case studies supported this “male combat hypothesis”. For example, in two distantly related species, the tusked frog (*Adelotus brevis*) and the fanged frog (*Limnonectes kuhlii*), males are larger than conspecific females and have larger paired projections in their low jaw [12,13]. Males defend their calling sites by attacking rival males using their fangs or tusks; hence larger body size and larger fangs or tusks may provide males with a better chance of winning battles and enhancing their reproductive success [13]. Zheng *et al*. [14] also found a positive correlation between the presence of weapon-like keratinized maxillary spines and male-biased SSD in megophryid frogs of the genus *Leptobrachium*. Hudson *et al.*[15] confirmed that the Emei mustache toads (*L. boringii*) indeed use the spines (“moustache”) as weaponry to fight their rivals. Other life history traits, such as parental care, length of breeding/growth season, developmental rate/time and ages of sexual maturity have also been explored in anurans [16-19].

Several phylogenetic comparative methods (PCMs) have been developed [20,21] since Shine’s study in 1979 [11], which provide better tools to analyse correlated evolution.Closely related species share phenotypic similarities inherited from a common ancestor, so direct correlation studies that treat each species as an independent data point tend to overestimate the degrees of freedom for statistical tests. Differing from traditional cross-species analysis, PCMs correct this statistical non-independence within a phylogenetic framework. The advent of DNA sequence analysis and the development of phylogenetic methods in the last few decades have also led to an explosion of new phylogenies of many major organism groups. Anurans are no exception; several large-scale phylogenies have been reconstructed (*e.g.*[22,23]). Additionally, tremendous amounts of data on body size and life history have been accumulated for many anuran species since Shine’s study. Together, these new tools and new data provide an excellent opportunity to utilize phylogenetically corrected analysis to re-examine the role of life history in shaping SSD in anurans.

We attempt a comprehensive evaluation of SSD in all anurans with two objectives. First, we assess the overall patterns of SSD and test for Rensch’s rule in anurans. Second, we investigate correlated evolution between SSD and life history traits, particularly traits that are related to fecundity, combat, and parental care behaviour. We used size dimorphism index (SDI; Body-size_female_/Body-size_male_ -1) [24] to represent SSD, and all tests were controlled for evolutionary relatedness among species using PCMs. We made several predictions based on current understanding of SSD evolution and observations in anurans, assuming that inter-specific differences reflect a history of selection at an intra-specific level: 1) Egg size and clutch size are positively correlated with female body size and SDI (fecundity advantage hypothesis). 2) The presence of male mating combat behaviour is positively correlated with male body size and negatively correlated with SDI (male combat hypothesis). 3) Rensch’s rule predicts a negative correlation between the ratio of female to male body size and the mean body size of a species for a group with female-biased SSD as the predominate form. How parental care is associated with SSD is uncertain; it may be positively or negatively correlated with the body size of the care providing sex and hence SSD.

## Methods

### Taxa sampling

Body size, life history, and phylogenetic data were collected from published literature. For behavioural data, only species with sufficiently detailed descriptions were used. Thus, many species in Shine’s study [11] were not included. In total, body size and life history data were collected for 688 anuran species, including 534 species with body size data and at least one life history trait. For the remaining 154 species, we did not have body size information. Among them, 119 species had egg size and clutch size data and were used for correlation analysis; 36 species had only mating combat and/or parental care information and they were not used for correlation analysis but used for detecting phylogenetic signals of these traits. In total, the 688 species represented approximately 10% of the described extant anuran species [25]. The majority of the data came from six groups: the superfamily Dendrobatoidea (family Aromobatidae and family Dendrobatidae; 56 species), the families Bufonidae (70 species), Dicroglossidae (37 species), Hylidae (191 species), Megophryidae (36 species), and Ranidae (115 species). The remaining 183 species were scattered across 35 other families. Species names and family names followed the “Amphibian Species of the World version 5.5” website as of March 2012 [25]. All body size and life history data used in this study are presented in Additional file 1, and their sources are provided in Additional file 2.

### Body size and life history traits

#### Body size

Snout-vent length (SVL) was used to represent anuran body size. Different publications provided different levels of detail for body size measurement, and consequently, the body size data were separated into two categories: mean body size (‘mean’) and body size range (‘range’). ‘Mean’ referred to body size data given as a mean SVL, and it required measurements with a minimum sample size of three for each sex per species. ‘Range’ included body size provided as maximum and minimum values, and also included body size measured on only one or two individuals in each sex. If more than one study provided ‘mean’ data, an unweighted average across studies was calculated for each sex. This criterion was also applied for other life history traits that were reported in multiple sources. Body size of a species was represented by the mean of both sexes.

All analyses pertaining to body size and sexual size dimorphism were conducted twice: once with only the high quality data, the ‘mean’ data, and once with pooled ‘mean’ and ‘range’ data. For ‘range’ data, the midpoint of the range was used to represent the body size in each sex. Although the ‘range’ data are not as accurate as the ‘mean’ data, they represent an additional 154 species (Table 1), and sample size is important for statistical tests (*e.g.* AIC scores).

**Table 1:** Sample sizes for body size data and all other life history traits

**Traits**	**Number of species**
Body size (mean)	380
Body size (range)	154
Egg size	440
Clutch size	386
Male combat	121
Male scramble competition	56
Male territory defence	70
Male combat in both forms	9
Male combat in unknown form	4
Female combat	12
Combat in both sexes	9
Absence in mating combat	475
Parental care	130
Male parental care	92
Female parental care	48
Parental care in both sexes	18
Paternal care of unknown sex	8
Absence in parental care	418

The numbers may not sum up because some species may perform both male and female combat or male and female parental care.

#### Sexual size dimorphism

SSD was treated as a continuous trait using size dimorphism index (SDI) [24]. SDI was estimated by taking the ratio of female to male body size and subtracting one (Body-size_female_/Body-size_male_ -1). The index has been widely used in many previous studies because of its intuitive appeal for both direction and degree of SSD as well as other advantages [2].

#### Egg size and clutch size

Egg size was represented by the diameter of the ovum. Clutch size referred to the number of eggs a female laid in a clutch. When females produced multiple clutches in a year, we only used the reported size of a single clutch for that species. The mean values of egg size and clutch size were preferentially used, but the median of a range was accepted when a mean value was not available. Egg size and clutch size are potentially correlated with female body size and SDI, and therefore, we considered both as explanatory variables in a linear multiple regression analysis. In the full model of the regression, we considered the effects of both traits and their interaction.

#### Mating combat

The presence of mating combat was defined as observing aggressive physical contests between individuals of the same sex to gain access to mates or attractive territories [26]. The absence of a record of mating combat may result from lack of observation. Therefore, only species for which other mating behaviours had been observed with no mention of combat were considered as exhibiting no form of mating combat. Such mating information includes descriptions of nest building, mate searching, advertisement call, male satellite behaviour, and breeding aggregation. In contrast to Shine [11], tusks and spines on male’s body were not viewed as signals of combat potential, as they might be primarily developed and generally used for other purposes, such as aids in amplexus, anti-predation or foraging adaptations [8]. Both male and female anurans are known to partake in combat [27], therefore, each sex was analysed separately. Species, in which both males and females exhibited combat behaviour, were included in both male and female combat analyses.

#### Parental care

Parental care, defined as any form of post-ovipositional parental investment that increases the survival of offspring [10], was reviewed based on descriptions in the literature of parental attendance of clutches, building of foam nests when laying eggs, carrying eggs, transporting tadpoles, larval development in or on parents’ bodies, and female deposition of unfertilized eggs to feed tadpoles [10]. The absence of parental care was defined as no evidence for any of these behaviours when other breeding information was available. This breeding information includes the descriptions of breeding habitat, egg development habitat, breeding behaviour during egg-laying, egg developmental time, egg size, and clutch size. Parental care in the two sexes was analysed separately. Species with both parents exhibiting parental care were included in both analyses.

### Phylogenetic comparative analysis

We used two PCMs to remove phylogenetic autocorrelation between species: multiple regression with a phylogenetic generalized least squares (PGLS) model [28,29] and phylogenetic independent contrasts (PIC) [20,30]. All analyses were conducted using the ‘Comparative analysis of phylogenetics and evolution package in R (caper)’ [31]. PCMs require a phylogenetic tree, and we used Pyron & Wiens’ (2011) tree of amphibians [23], which represents the most recent and comprehensive phylogeny of anurans. Anuran classification is undergoing major revisions (*e.g.*[22]), and some formerly recognized species have been split into several new species. In these cases, we matched the source populations of life history data with the species in the phylogeny.

Anuran families differ dramatically in mating systems and reproductive strategies, and so the correlated evolution of certain life history traits may be specific to certain families. Accordingly, most analyses were conducted at two levels: across all anuran species (all-anuran analysis) and within each of the six families or superfamily (within-family analysis). Species in the superfamily Dendrobatoidea, including the families Dendrobatidae and Aromobatidae, have many life history similarities [8,32], therefore, they were analysed together. It was noteworthy that, for each correlation analysis, only species with data for both traits were used; species with missing data were automatically pruned from the analyses in ‘caper’. Consequently, the sample size in each correlation analysis on given traits was always smaller than the number of species in a group. Since each comparison had a different composition of species, a Bonferroni correction could not be applied [33].

#### Testing phylogenetic signals of traits

We used the *D* statistic [34] and a phylogenetic parameter *λ*[29] as the measures of phylogenetic signals of life history traits. *D* is applicable only for binary traits, and was carried out with the ‘phylo.d’ function in ‘caper’. *D* typically varies between 0 and 1. A *D* of 0 indicates that a trait evolves on a tree following the Brownian model (strong phylogenetic signal), and a *D* of 1 indicates that a trait evolves following a random model (no phylogenetic signal). *D* can be negative, which means that a trait evolves in a conserved way: more conserved than predicted by the Brownian model. Additionally, we conducted a simulation (1000 permutations) to test whether an estimated *D* was significantly different from the predictions of a random or a Brownian style evolution.

The parameter λ is applicable for both continuous and binary traits, which reveal the dependence among species for a given phylogeny and a given trait. Its optimum value and confidence limits were estimated using the ‘pgls’ function in ‘caper’ with a maximum likelihood method when performing a correlation analysis. λ shows the strength of the phylogenetic signal of a phylogenetic generalized linear model, which ranges between 0 and 1. When λ=0, the variation of a trait is modeled as a function of an independent evolution along the branches leading to the tips. When λ=1, the trait shows variation expected under the Brownian model. The estimated λ was used not only for measuring strength of phylogenetic signal, but also for transformation of internal branch lengths for phylogenetic linear model analysis.

#### Multiple regression analysis using a phylogenetic generalized least squares model

PGLS includes a phylogenetic tree as a covariance matrix in a linear model [31]. It is capable of evaluating multiple causative variables simultaneously and incorporating polytomies [35].

We first used a model selection approach to test the relative importance of all life history traits and their interactions. The SDI was set as the response variable, and initially egg size, clutch size, male combat, and parental care were included as explanatory variables in the model. Other measured variables either overlap with male combat (*i.e.* male scramble competition and male territory defence) or with parental care (*i.e.* male and female parental care), so they are not independent predictors and cannot be used in the same model. To further test the combat hypothesis, female combat and the two different forms of male combat were analysed separately by replacing male combat with each of them. Similarly, female parental care and male parental care were analysed separately by replacing parental care with each of them to further explore the parental care hypothesis. From the saturated full model, including four explanatory variables and their interactions, we excluded the non-significant interactions and variables step by step. The reduced model was then compared to the unreduced model using the ‘anova’ function, and the difference in AIC scores was used to determine the significance of the exclusion. Model comparison requires complete data for each species. Consequently, approximately 68% of the species were excluded from this analysis because of missing data in at least one of the variables. This dataset with no missing values is denoted as the ‘reduced dataset’. The analysis was conducted for all-anurans and for each of the six (super)families, and was carried out using the ‘pgls’ function in ‘caper’.

Branch lengths were transformed by three scaling parameters, λ, κ, and σ in order to make the data fit the Brownian model of evolution. Although all of them could be optimized by ‘caper’, altering three parameters simultaneously would make the biological interpretation difficult [31]. Consequently, we only estimated the parameter λ, because it was significantly different from its upper and lower bound values (0 and 1) for all traits. The other two parameters were kept constant with their default values. The λs for ‘range + mean’ data and ‘mean’ data were estimated separately because of different accuracy in body size measurements. The λs used in the family-level analysis were the same λs estimated for all-anurans, because they were based on more data and thus were more likely to be accurate.

We further tested the correlation between SDI and each of the explanatory variables independently (egg size and clutch size were analysed in combination), as well as between several pairs of life history traits. Compared with the model selection approach, this simple linear regression analysis only excluded species with missing values of the focal traits, and therefore, utilized the most available data and likely had higher statistical power to detect a correlation. The full dataset used in this set of analyses is denoted as the ‘extended dataset’. It was noteworthy that since several traits (*e.g*. SDI) were repeatedly used in several linear regressions, the Type I errors of this set of analyses were inevitably inflated.

#### Comparative analysis with phylogenetic independent contrasts

PIC only allows pairwise correlation analysis. We used the ‘crunch’ function in ‘caper’ to calculate standardized contrasts of all continuous traits. This function then used a simple linear model to test for a correlation between contrasts of two continuous traits. We used the ‘brunch’ function to test for correlation between a continuous trait and a categorical trait. ‘Brunch’ estimates independent contrast values of a continuous trait on the nodes where the state of the categorical variable changes [36]. A one-sample *t*-test was then used to determine if the mean of the independent contrasts of the continuous response variable was significantly different from zero. After running ‘crunch’ and ‘brunch’, the ‘caic.diagnostics’ function in ‘caper’ carried out diagnostic tests for the robustness of contrasts and identified the outlier contrasts that had absolute studentised residuals greater than three. The outlier contrasts might bias the correlation analysis, and so were filtered out using the ‘caic.robust’ function before the final analyses [31]. Ultimately, all diagnostic tests of our correlation analyses were insignificant, indicating our data met the assumption of evolution under a Brownian model.

#### Testing Rensch’s rule

Rensch’s rule predicts that the magnitude of SSD decreases along with the increase of body size in anurans where females are generally the larger sex [4]. This pattern was detected as a slope significantly smaller than one when male body size was on the x-axis in a major-axis regression between the independent contrasts of logarithm transformed male and female body size [37]. A major-axis regression through the origin was conducted in R [38].

## Results

### General pattern of SSD in anurans

**Figure 1:**
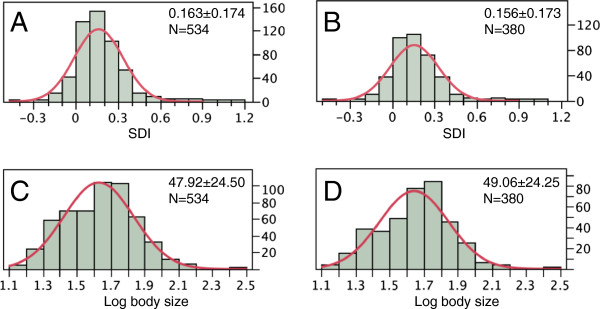
**Distributions of logarithm transformed body size and sexual dimorphism index.** SDI = Body-size_female_/Body-size_male_ –1. **A **&**C.** Range + mean data. **B** &**D.** Mean data only.

This study presents the most comprehensive study of SSD in anurans to date. Of the 688 species that we could gather data for, body size data were available for 534 species, within which 380 had ‘mean’ data, and 154 had ‘range’ data. The sample size for each trait was different; all traits and their sample sizes are presented in Table 1. The distributions of all continuous traits (SDI) or their logarithm-transformed forms (body size, egg size, and clutch size) were close to normal. Figure 1 presents the distribution of SDI and log body size.

Body size (SVL) had an average of 47.92 mm for the ‘range + mean’ data and 49.06 mm for the ‘mean’ data (Figure 1), and ranged from 13.35 mm in the blue-bellied poison frog (*Ranitomeya minutus*) to 260.00 mm in the goliath frog (*Conraua goliath*) (Additional file 1). The majority of species (97%) had body sizes smaller than 100 mm.

Sexual dimorphism in anurans was predominantly biased towards females (Figure 1). The distribution of SDI was close to normal, with an average of 0.163 for the ‘range + mean’ data and 0.156 for the ‘mean’ data. Male size was found to significantly exceed the size of conspecific females in only nine species, although 58 species (10.9%) with ‘range + mean’ data and 48 species (12.6%) with ‘mean’ data had negative SDIs. The magnitude of male-biased SSD was similar to the magnitude of female-biased SSD. Females could be almost twice the size of conspecific males (*e.g.* 188% in the odorous frog *Odorrana schmackeri*[39]; Additional file 1), while the African bullfrog (*Pyxicephalus adspersus*)*,* which demonstrated an extreme male-biased anuran SSD, had male sizes 176% that of conspecific females [40].

Species with negative SDI (male-biased SSD) were present in 19 anuran families (Aromobatidae, Bufonidae, Bombinatoridae, Centrolenidae, Cryptobatrachidae, Dendrobatidae, Dicroglossidae, Hylidae, Leiuperidae, Leptodactylidae, Limnodynastidae, Mantellidae, Megophryidae, Microhylidae, Myobatrachidae, Pelobatidae, Petropedetidae, Pipidae, Pyxicephalidae, Ranidae, Rhacophoridae, and Scaphiopodidae) and distributed across six continents (Asia, Africa, Australia, Europe, North America, and South America) in both temperate and tropical zones. Some of these species were not included in the final analysis because of a lack of other life history information.

### The impact of phylogeny on the evolution of life history traits

The *D* statistics of all but one of the binary traits fell between 0 and 1 (Additional file 3). The evolution of all traits was significantly different from random evolution, and the evolution of three binary traits (female combat, parental care, and male parental care) did not differ significantly from the Brownian model (p > 0.05). *D* of ‘male parental care’ was < 0, indicating the evolution of this trait was more conservative than predicted by the Brownian evolution model. Lambdas (*λ*) were between 0 and 1 for all models, and were significantly different from the two bounds, indicating that the evolution of all the traits depended on their phylogenetic history (not random), but might not strictly follow the Brownian model.

Clearly, the evolution of all traits was phylogenetically constrained at various degrees. Therefore, including the phylogenetic perspective in the comparative analysis was essential. In the meantime, the evolutionary history of these traits did not strictly follow the Brownian evolution, on which all our analyses were based, and therefore, branch length transformation was necessary to best-fit the evolution of these traits to the Brownian model.

### Correlations between SSD, body size, and life history traits

#### Comparative analysis using PGLS

Based on the ‘reduced dataset’, the model selection analysis for all-anurans found that the best model was SDI to be negatively correlated with parental care, and with male parental care in particular (Table 2). Parental care was the only significant, and the most influential, variable that might explain SDI (p < 0.01). Both ‘range + mean’ data and ‘mean’ data supported this correlation. Family level analysis revealed several correlative relationships within specific families (Table 2): a positive correlation between SDI and egg size in the families Bufonidae and Ranidae, a negative correlation between SDI and parental care in the family Hylidae, a positive correlation between SDI and clutch size and negative correlations between SDI and female parental care in the superfamily Dendrobatoidea, and a negative correlation between SDI and parental care in the family Hylidae.

**Table 2:** Summary of the best models from model selection analysis on the ‘reduced dataset’ using the phylogenetic generalized least square (PGLS) model

**All anurans**		**Bufonidae**		**Dendrobatoidea**		**Hylidae**	**Ranidae**	
‘Range + mean’ data	‘Mean’ data	‘Range + mean’ data	‘Mean’ data	‘Range + mean’ data	‘Mean’ data	‘Range + mean’ data	‘Range + mean’ data	‘Mean’ data
SDI ~ PC	SDI ~ PC	SDI ~ ES	SDI ~ ES	SDI ~ CS	SDI ~ CS+	SDI ~ PC	SDI ~ ES	SDI ~ ES
λ = 0.797	λ = 0.799	λ = 0.810	λ = 0.827	λ = 0.805	FPC	λ = 0.823	λ = 0.810	λ = 0.827
df = 213	df = 164	df = 21	df = 13	df = 25	λ = 0.603	df = 128	df = 70	df = 65
t = −3.43	t = −2.91	t = 2.43	t = 1.89	t = 3.39	df = 21	t = −2.54	t = −2.16	t = −1.94
p < 0.01	p < 0.01	p = 0.02	p = 0.08	p < 0.01		p = 0.01	p = 0.03	p = 0.06
AIC = −182.21	AIC = −138.14	AIC = −19.94	AIC = −24.75	AIC = −93.30	CS:	AIC = −161.42	AIC = −32.61	AIC = −32.50
SDI ~ MPC	SDI ~ MPC				t = 2.63			
λ = 0.810	λ = 0.783				p = 0.02			
df = 213	df = 164				FPC:			
	t = −3.81				t = −3.91				t = −3.03
	p < 0.01				p < 0.01				p < 0.01
AIC = −184.97	AIC = −144.52				AIC = −86.52				

SDI is the response variable and life history traits are the explanatory variables, and species with missing values on any of the traits are excluded. PGLS is conducted at both all-anuran and family levels. No significant models are detected in the families Dicroglossidae and Megophryidae using either ‘range + mean’ or ‘mean’ data, and in the family Hylidae using ‘mean’ data.

*SDI* sexual size dimorphism index, *CS* log clutch size, *ES* log egg size, *PC* parental care, *MPC* male parental care, *FPC* female parental care, and *AIC* Akaike information criterion.

Simple linear regression analysis based on the ‘extended dataset’ of all-anurans again showed that SDI was negatively correlated with parental care (p < 0.01), particularly with male parental care (p < 0.01; Table 3). In addition, SDI was negatively correlated with egg size (p = 0.05). Furthermore, female body size was positively correlated with both egg size and clutch size (p < 0.01), and parental care, particularly female parental care, was negatively correlated with clutch size (p < 0.01). Nevertheless, none of the combat behaviour was correlated with SDI or body size, except for a marginal correlation between SDI and male territory defence (p = 0.09; Table 3). More detailed results are provided in Additional file 4.

**Table 3:** Summary of the simple linear regression analysis on the ‘extended dataset’ using phylogenetic generalized least square (PGLS) model

**Models**	**FBS ~ ES + CS**	**SDI ~ ES + CS**	**SDI ~ MTD**	**SDI ~ PC**	**SDI ~ MPC**	**CS ~ PC**	**CS ~ FPC**	**ES ~ MPC**
‘Range + Mean’ data	λ = 0.966		λ = 0.805	λ = 0.902	λ = 0.901	λ = 0.964	λ = 0.961	λ = 0.963
	R^2^ = 0.36		df = 453	df = 407	df = 402	df = 371	df = 366	df = 404
	df = 2, 249		t = −1.71	t = −3.21	t = −2.86	t = −2.84	t = −2.87	t = 1.74
	ES: p < 0.01		p = 0.09	p < 0.01	p < 0.01	p < 0.01	p < 0.01	p = 0.08
	Slope = 0.46							
	t = 8.06							
	CS: p < 0.01							
	Slope = 0.15							
	t = 10.24							
‘Mean’ data	λ = 0.970	λ = 0.895		λ = 0.899	λ = 0.894			
	R^2^ = 0.41	df = 255		df = 295	df = 291			
	df = 2, 196	R^2^ = 0.02		t = −2.59	t = −2.84			
	ES: p < 0.01	ES: p = 0.05		p = 0.01	p < 0.01			
	Slope = 0.49	Slope=						
	t = 7.92	−0.15						
	CS: p < 0.01	t = −1.99						
	Slope = 0.16							
	t = 9.96							

All species with relevant data are included in each pairwise correlation analysis. Only correlations with degrees of freedom ≥ 3 and p < 0.1 are presented here, and the complete results are available in Additional file 4.

*FBS* female body size, *CS* log clutch size, *ES* log egg size, *SDI* sexual size dimorphism index, *MTD* male territory defence, *PC* parental care, *MPC* male parental care, and *FPC* female parental care.

Overall, the presence of parental care behaviour was clearly associated with SDI and fecundity, but its correlation with body size was less evident. The fecundity traits were correlated with SDI and female body size. On the other hand, considering previous findings in anurans (*e.g*. [11]) and patterns of SSD in other animal groups (*e.g*. mammals and birds [2]), the lack of significant correlation between male combat behaviour and SSD was surprising.

#### Comparative analysis using PIC

PIC analysis produced similar results to that of PGLS. For example, it detected a negative correlation between SDI and parental care, male parental care in particular, and a positive correlation between female body size and both egg size and clutch size in all-anuran analysis. The two analyses also agreed on several correlations at the family level, *e.g.* clutch size was positively correlated with SDI in the superfamily Dendrobatoidea, and parental care was negatively correlated with SDI in the family Hylidae.

PIC analysis revealed several additional significant correlative relationships within individual families (Additional file 4). In Dendrobatoidea, male body size and male combat behaviour were positively correlated (p = 0.04), and female body size and female parental care were negative correlated (p < 0.05). In Hylidae, SDI was negatively correlated with female parental care (p < 0.05). Additionally, PIC had several minor disagreements with PGLS on the importance of different fecundity traits. For example, PGLS suggested that SDI was positively correlated with egg size in Bufonidae, but PIC suggested a positive correlation with clutch size. Complete results of PIC analysis for both all-anurans and individual families are presented in Additional file 4.

#### Rensch’s rule

**Figure 2:**
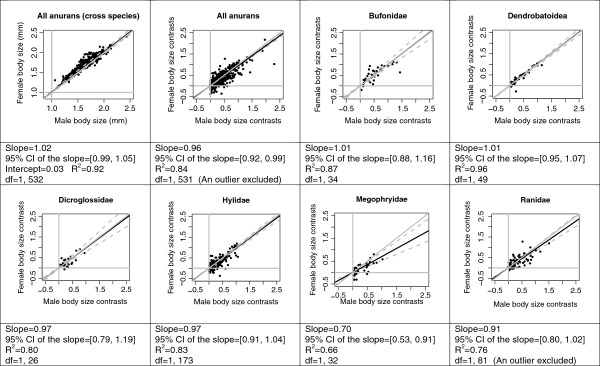
**Test of Rensch’s rule.** Asymmetry for sexual size dimorphism in anurans using major-axis regressions between independent contrasts of logarithm transformed male and female body sizes. Black solid lines: major-axis regression line; grey dash lines: confidence intervals of the major axis regression; grey solid lines: slope = 1.

The independent contrasts of log female body size and log male body size were significantly correlated with each other in the all-anuran analysis and in every within-family analysis (p < 0.01, Figure 2). The slopes were significantly lower than one in the all-anuran analysis and in the family Megophryidae, which was consistent with the prediction of Rensch’s rule (Figure 2). The slope and its upper confidence limit in the all-anuran analysis, however, were close to one (slope = 0.96, upper confidence limit = 0.99), and therefore, the negative correlation between body size and SSD was statistically significant but biologically weak in anurans. With an increase of body size, male body size increased only slightly faster than female body size. Slope of one was within the confidence intervals of all other families.

## Discussion

### Sexual size dimorphism in anurans

Female-biased SSD is the predominant pattern in anurans (Figure 1). This is congruent with Shine’s early assessment [11]. Deviation or reversal from this common pattern has independently evolved many times and its occurrence is not restricted to certain lineages or in certain geographic regions. SSD is relatively small in magnitude, and in the species with the largest SSD, females do not exceed twice the size (snout-vent length) of conspecific males. Male-biased SSD in anurans, however, are among the most extreme cases. Male African bullfrogs exceed 1.7 times the size of conspecific females, which is comparable to the most extreme known case in animals, in which the males are twice the size of females (cichlid fish *Lamprologus callipterus*) [41].

The SSD in anurans shows a weak trend that is consistent with Rensch’s rule. The trend is statistically significant but biologically weak in anurans as a whole, and it is apparent only in the family Megophryidae (Figure 2). The weak, or lack of, support for Rensch’s rule in anurans is not particularly surprising. Supports for the rule are mostly seen in groups that have a male-biased SSD [37], and exceptions to the rule are commonplace [2]. Anurans have a high diversity of life history traits, which may also contribute to the weak support for Rensch’s rule, as many of these traits may alter the general pattern of body size dimorphism.

### Correlated life history traits

The negative correlation between SDI and parental care provides clear evidence for the ‘parental care hypothesis’ that parental care directly contributes to the patterns of SSD (Tables 2 and 3). The idea that male parental care may drive the evolution of male-biased SSD in anurans has recently been suggested several times, but has never been formally tested (*e.g.*[16,42]). Similar to fish [43], but unlike most other vertebrate groups, male anurans are the primary care providers (Table 1). The correlation between SDI and parental care may be mostly contributed by the species with male care in anurans, as a significant correlation between SDI and male parental care was found in all-anurans. Nevertheless, significant negative correlation between SDI and female parental care was also found in the (super) families Dendrobatoidea (PGLS) and Hylidae (PIC).

The mechanisms linking parental care and SSD remain to be tested. Several hypotheses have been proposed. First, parental care may improve the survival of offspring and hence ‘relax’ fecundity selection on female body size, which may reduce the magnitude of female-biased SSD or reverse its direction. Parental care and clutch size were negatively correlated in anurans (Table 3 & Additional file 4), which is consistent with the prediction of this ‘relaxed’ fecundity selection scenario. Summers *et al.*[44] also reported a significant positive correlation between parental care and egg size in anurans, although we only detected a marginal correlation between male parental care and egg size (Table 3). This correlation has also been documented in many other vertebrate groups, *e.g*. fishes [45]. Furthermore, male-biased SSD is often present in lineages where females do not produce large numbers of eggs, such as those in the families Bombinatoridae, Megophryidae, the superfamily Dendrobatoidea, and some clades in the family Hylidae (Additional file 1). Second, some forms of parental care, such as protecting offspring from predation, may demand strength or size of the parents [8,10]. Males in some species with male-biased SSD, such as the African bullfrog (*Pyxicephalus adspersus*), were observed to defend tadpoles against vertebrate predators [46]. Third, the care-giving parents may benefit from large amount of fat storage in their body, and hence large body size. Looking after young may require additional energy consumption and/or a reduction in energetic intake [10]. Case studies also demonstrate significant body weight loss during the breeding period in Emei mustache toads (*Leptobrachium boringii*) [16]. We did not find support for the last two hypotheses because of the lack of any positive correlation between parental care and body size in both genders. Furthermore, the negative correlations between female body size and female parental care (PIC; Additional file 4) in Dendrobatoidea, and between SDI and female parental care in both Dendrobatoidea (PGLS; Table 2) and Hylidae (PIC; Additional file 4) are consistent with the first hypothesis, but not with the last two. Therefore, our data support the relaxed fecundity hypothesis, although it does not necessarily reject the other two alternatives.

Fecundity advantage appears to select for large females in anurans, and considering that nearly 90% of all anurans have female-biased SSD, it certainly is a leading selecting force in shaping SSD in this group. The increase of female body size is strongly linked to the increase of egg size and/or clutch size in all-anurans as well as in individual families (Table 3 & Additional file 4). This pattern is congruent with observations from the majority of animal groups [2], and is consistent with the fecundity advantage hypothesis [1] (but see [47]). Furthermore, significant correlation between SDI and the fecundity-related traits (egg size and/or clutch size) was found in all-anurans and several families (Additional file 4). The positive correlations between these fecundity-related traits with SDI and female body size provide support for fecundity driven SSD evolution. Female body size may have driven the evolution of female-biased SSD through selection on egg size and clutch size. On the other hand, the relaxation of selection on female fecundity and accordingly on female body size, may result in male-biased SSD.

Surprisingly, our data do not support the ‘male combat hypothesis’. We did not detect any significant correlations between SDI and any male combat behavioural traits. Significant correlation between male body size and male combat behaviour was detected only in the superfamily Dentrobatoidea (PIC, Additional file 4). This weak, or lack of, correlation is contradictory to the well-established concept that male-male competition (sexual selection) drives to increase male body size and leads to male-biased SSD. Within anurans, Shine’s early evaluation and several case studies also associated the combat behaviour with male-biased SSD [11-13,15]. This apparent contradiction is unlikely the result of small sample sizes, and hence low statistical power in our analysis. We have 599 species with combat (or lack of combat) information (Table 1). In the all-anuran analysis with PIC, the number of contrasts between SDI and male combat is 73 (‘range + mean’ data) and 51 (‘mean’ data only), respectively (Additional file 4). We are convinced that the lack of support for the ‘male combat hypothesis’ in anurans is real and there are several potential causes. First, male body size may influence mating success in some anuran species, but may have no impact in others [48]. Second, the common occurrence of alternative mating tactics in anurans, such as satellite male behaviour [10], may reduce the selection pressure on male body size. Small males may gain fitness by using alternative mating tactics. For example, while large male bullfrogs (*Rana catesbeiana*) are territory defenders, small males are often satellites, and satellite males may account for as much as 20% of the total mating in a population [49]. Third, the extreme diversity of mating systems, life history traits and their plasticity in anurans may mask the potential contribution of male-male competition to body size evolution. The few case studies that clearly link them together likely represent extreme cases, as a consequence of researchers sampling bias. For example, in all three aforementioned case studies [12,13,15], males have weaponry structure and are significantly larger than conspecific females. These characters make them more attractive to be study objects than others.

It is worthwhile to note that the three alternative hypotheses, parental care, fecundity advantage, and male combat, are not mutually exclusive. In nature, different selection forces may complement or oppose each other, and their interactions may vary among taxa. Therefore, the sparse or lack of significant correlation in many cases may reflect the true complexity of various selection forces that are involved in the evolution of body size and SSD.

## Conclusions

Sexual size dimorphism in anurans is predominantly female-biased and fecundity advantage may play a significant role in determining this pattern. Paternal care in anurans may lead to deviations from this common pattern and contribute to the evolution of male-biased SSD. This discovery provides a new insight in understanding the evolution of SSD: natural selection, as well as sexual selection, can drive male-biased SSD. More research in fish, amphibians and invertebrates are needed to understand the general patterns of SSD in animals.

A large number of detailed case studies may be necessary to understand the alternative functional explanations of SSD. Correlations are not causations, but well-established correlations provide guidance for future experimental work, and only the latter can unambiguously establish causation relationships. Species in the superfamily Dendrobatoidea, and the families Dicroglossidae and Megophryidae exhibit both male-biased and female-biased SSD in a large number of clades (*e.g.*[50]). They may provide a fertile ground for both family level comparative studies and detailed case studies.

### Availability of supporting data

The data supporting the results of this article are included within the article and its additional files.

## Abbreviations

AIC: Akaike information criterion; PCM: Phylogenetic comparative method; PGLS: Phylogenetic generalized least squares model; PIC: Phylogenetic independent contrasts; SDI: Sexual dimorphism index; SSD: Sexual size dimorphism; SVL: Snout-vent length.

## Competing interests

The authors declare that they have no competing interests.

## Authors’ contributions

XH collected most of the data, conducted most analyses and drafted the manuscript. JF conceived the project, helped with the data collection and analysis, and finalized the manuscript. Both authors read and approved the final manuscript.

## Authors’ information

XH is currently a PH.D candidate at the Queen’s University and her primary interests are the evolutionary consequences of sperm senescence in *Drosophila melanogaster*. JF is a herpetologists and is interested in a broad range of evolutionary questions.

## Supplementary Material

Additional file 1 **Mean body size in each sex, mean egg size, mean clutch size, mating combat, and parental care behavior in 688 anuran species.** SDI = sexual dimorphism index [(female body size/male body size) – 1]. * R = range body size data, M = mean body size data. ^$^ The presence of a trait is coded as “1”, and the absence of a trait is coded as “0”; missing data are represented by “-”. Click here for file

Additional file 2 **References for body size, egg size, clutch size, mating combat, and parental care in 688 anuran species.** The general mating or breeding descriptions are used to define the absence of mating combat or parental care behaviour, respectively. Click here for file

Additional file 3 **The*D*statistic for all binary traits.** The non-significant *p* values are in bold, which means the traits are under Brownian evolution. Click here for file

Additional file 4 **Results from simple linear regression analyses on the ‘extended dataset’ using phylogenetic generalized least squares (PGLS) model and phylogenetic independent contrasts (PIC).** PIC analyses are conducted at both all-anuran and family levels; outliers, which deviated from the majority of the data points by more than three interquartile ranges from the quartiles, were excluded. All species with relevant data are included in each pairwise correlation analysis. Only correlations with degrees of freedom ≥ 3 are presented. P < 0.10 are in bold. * Dicro = Dicroglossidae; the family has ‘mean’ data only. Click here for file
