# Supplementary material for: Does life history shape sexual size dimorphism in anurans? A comparative analysis
Source: BMC Evol Biol. 2013 Jan 31;13:27. doi: 10.1186/1471-2148-13-27 (PMC3570426; doi:10.1186/1471-2148-13-27)
Supplement: Additional file 1 — Mean body size in each sex, mean egg size, mean clutch size, mating combat, and parental care behavior in 688 anuran species. SDI = sexual dimorphism index [(female body size/male body size) – 1]. * R = range body size data, M = mean body size data. $ The presence of a trait is coded as “1”, and the absence of a trait is coded as “0”; missing data are represented by “-”. [file 1471-2148-13-27-S1.docx]

**Additional file 1: Mean body size in each sex, mean egg size, mean clutch size, mating combat, and parental care behavior in 688 anuran species.** SDI = sexual dimorphism index [(female body size / male body size) – 1]. * R = range body size data, M = mean body size data. ^$^ The presence of a trait is coded as “1”, and the absence of a trait is coded as “0”; missing data are represented by “-”.

| **Family** | **Species** | **Female Body Size (mm)** | **Male Body Size (mm)** | **SDI** | **Range or Mean*** | **Egg Size (mm)** | **Clutch Size** | **Male Combat**^$^ | **Female Combat**^$^ | **Male Scramble Competition**^$^ | **Male Territory Defense**^$^ | **Male Parental Care**^$^ | **Female Parental Care**^$^ | **Paternal Care**^$^ |
| --- | --- | --- | --- | --- | --- | --- | --- | --- | --- | --- | --- | --- | --- | --- |
| Allophrynidae | *Allophryne ruthveni* | 27.0 | 22.6 | 0.19 | R | - | 300 | 0 | 0 | 0 | 0 | 0 | 0 | 0 |
| Alytidae | *Alytes obstetricans* | - | - | - | - | 2.6 | 53 | 0 | 1 | 0 | 0 | 1 | 0 | 1 |
| Alytidae | *Discoglossus pictus* | - | - | - | - | 1.3 | 750 | 0 | 0 | 0 | 0 | 0 | 0 | 0 |
| Aromobatidae | *Allobates brunneus* | 19.6 | 17.9 | 0.09 | M | - | - | - | - | - | - | - | - | - |
| Aromobatidae | *Allobates caeruleodactylus* | 16.3 | 15.5 | 0.05 | M | - | - | 1 | 0 | 0 | 1 | 1 | 0 | 1 |
| Aromobatidae | *Allobates femoralis* | 26.9 | 24.9 | 0.08 | M | 2.0 | 43 | 1 | 0 | 1 | 1 | 1 | 0 | 1 |
| Aromobatidae | *Allobates nidicola* | 20.2 | 19.6 | 0.03 | M | 2.4 | 4 | 0 | 0 | 0 | 0 | 0 | 0 | 0 |
| Aromobatidae | *Allobates talamancae* | 24.0 | 20.4 | 0.18 | R | - | 18 | 1 | 0 | 0 | 1 | 1 | 1 | 1 |
| Aromobatidae | *Allobates zaparo* | 28.0 | 27.8 | 0.01 | M | - | - | - | - | - | - | - | - | 1 |
| Aromobatidae | *Anomaloglossus beebei* | - | - | - | - | 2.1 | 4 | 0 | 0 | 0 | 0 | 1 | 1 | 1 |
| Aromobatidae | *Anomaloglossus stepheni* | 17.1 | 16.6 | 0.03 | M | - | 4 | 1 | 0 | 0 | 1 | 1 | 0 | 1 |
| Aromobatidae | *Mannophryne trinitatis* | 25.6 | 23.1 | 0.11 | M | - | 10 | 1 | 1 | 0 | 1 | 1 | 0 | 1 |
| Aromobatidae | *Rheobates palmatus* | - | - | - | - | 2.4 | 22 | 0 | 0 | 0 | 0 | 1 | 0 | 1 |
| Arthroleptidae | *Trichobatrachus robustus* | - | - | - | - | - | - | 0 | 0 | 0 | 0 | 1 | 0 | 1 |
| Bombinatoridae | *Barbourula busuangensis* | 85.3 | 84.8 | 0.01 | M | 5.9 | 78 | - | - | - | - | 0 | 0 | 0 |
| Bombinatoridae | *Bombina bombina* | - | - | - | - | 1.8 | 190 | 1 | 0 | 1 | 1 | 0 | 0 | 0 |
| Bombinatoridae | *Bombina fortinuptialis* | 56.5 | 58.4 | -0.03 | M | 2.8 | - | - | - | - | - | - | - | - |
| Bombinatoridae | *Bombina lichuanensis* | 63.4 | 58.4 | 0.09 | M | 3.4 | - | - | - | - | - | - | - | - |
| Bombinatoridae | *Bombina maxima* | 50.9 | 49.2 | 0.03 | M | 3.2 | - | 0 | 0 | 0 | 0 | - | - | - |
| Bombinatoridae | *Bombina orientalis* | 47.0 | 42.1 | 0.12 | M | 1.5 | 165 | 1 | 0 | 1 | 0 | 0 | 0 | 0 |
| Bombinatoridae | *Bombina variegata* | - | - | - | - | - | 72 | 1 | 0 | 1 | 1 | 0 | 0 | 0 |
| Brevicipitidae | *Breviceps mossambicus* | - | - | - | - | 4.0 | 25 | 0 | 0 | 0 | 0 | 0 | 0 | 0 |
| Bufonidae | *Amietophrynus garmani* | - | - | - | - | 1.2 | - | 0 | 0 | 0 | 0 | 0 | 0 | 0 |
| Bufonidae | *Amietophrynus gutturalis* | - | - | - | - | 1.5 | 20000 | 1 | 0 | 1 | 0 | 0 | 0 | 0 |
| Bufonidae | *Amietophrynus kisoloensis* | - | - | - | - | - | - | 0 | 0 | 0 | 0 | 0 | 0 | 0 |
| Bufonidae | *Amietophrynus maculatus* | - | - | - | - | 1.3 | - | 1 | 0 | 1 | 0 | 0 | 0 | 0 |
| Bufonidae | *Amietophrynus pardalis* | 109.0 | 94.8 | 0.15 | M | - | - | 1 | 0 | 1 | 0 | 0 | 0 | 0 |
| Bufonidae | *Amietophrynus regularis* | - | - | - | - | 1.5 | 15000 | 1 | 0 | 1 | 1 | 0 | 0 | 0 |
| Bufonidae | *Amietophrynus xeros* | - | - | - | - | - | - | 0 | 0 | 0 | 0 | 0 | 0 | 0 |
| Bufonidae | *Anaxyrus americanus* | 64.1 | 53.1 | 0.21 | M | 1.2 | 6000 | 1 | 0 | 1 | 0 | 0 | 0 | 0 |
| Bufonidae | *Anaxyrus boreas* | - | - | - | - | 1.7 | 5213 | 1 | 0 | 1 | 0 | 0 | 0 | 0 |
| Bufonidae | *Anaxyrus californicus* | - | - | - | - | 1.4 | 4714 | 0 | 0 | 0 | 0 | 0 | 0 | 0 |
| Bufonidae | *Anaxyrus canorus* | - | - | - | - | 2.1 | - | 1 | 0 | 1 | 0 | 0 | 0 | 0 |
| Bufonidae | *Anaxyrus cognatus* | - | - | - | - | 1.2 | 22148 | 1 | 0 | 1 | 0 | 0 | 0 | 0 |
| Bufonidae | *Anaxyrus debilis* | 36.5 | 35.1 | 0.04 | M | 1.0 | - | 0 | 0 | 0 | 0 | 0 | 0 | 0 |
| Bufonidae | *Anaxyrus exsul* | 50.9 | 51.6 | -0.01 | M | - | - | 1 | 0 | 1 | 0 | 0 | 0 | 0 |
| Bufonidae | *Anaxyrus fowleri* | 71.5 | 55.7 | 0.28 | M | - | 8000 | 0 | 0 | 0 | 0 | 0 | 0 | 0 |
| Bufonidae | *Anaxyrus hemiophrys* | - | - | - | - | - | - | 0 | 0 | 0 | 0 | 0 | 0 | 0 |
| Bufonidae | *Anaxyrus microscaphus* | - | - | - | - | 1.4 | 3650 | 0 | 0 | 0 | 0 | 0 | 0 | 0 |
| Bufonidae | *Anaxyrus nelsoni* | - | - | - | - | - | - | 0 | 0 | 0 | 0 | 0 | 0 | 0 |
| Bufonidae | *Anaxyrus punctatus* | - | - | - | - | 1.2 | - | 0 | 0 | 0 | 0 | 0 | 0 | 0 |
| Bufonidae | *Anaxyrus quercicus* | 28.9 | 27.5 | 0.05 | M | 1.0 | - | 0 | 0 | 0 | 0 | 0 | 0 | 0 |
| Bufonidae | *Anaxyrus retiformis* | 52.5 | 44.4 | 0.18 | M | - | - | 0 | 0 | 0 | 0 | 0 | 0 | 0 |
| Bufonidae | *Anaxyrus terrestris* | - | - | - | - | 1.2 | 4000 | 0 | 0 | 0 | 0 | 0 | 0 | 0 |
| Bufonidae | *Anaxyrus woodhousii* | - | - | - | - | 1.3 | 10500 | 1 | 0 | 1 | 0 | 0 | 0 | 0 |
| Bufonidae | *Ansonia malayana* | 27.0 | 22.1 | 0.22 | M | - | - | 0 | 0 | 0 | 0 | - | - | - |
| Bufonidae | *Atelopus chiriquiensis* | 42.5 | 31.0 | 0.37 | R | 2.1 | 364 | 1 | 0 | 0 | 1 | 0 | 0 | 0 |
| Bufonidae | *Atelopus senex* | 36.5 | 30.0 | 0.22 | R | - | - | 0 | 0 | 0 | 0 | - | - | - |
| Bufonidae | *Atelopus spumarius* | 35.0 | 27.5 | 0.27 | R | 2.0 | - | 0 | 0 | 0 | 0 | 0 | 0 | 0 |
| Bufonidae | *Atelopus varius* | 40.5 | 33.0 | 0.23 | R | 2.4 | 950 | 1 | 0 | 0 | 1 | 0 | 0 | 0 |
| Bufonidae | *Bufo aspinius* | 95.0 | 74.4 | 0.28 | M | - | 2250 | 1 | 0 | 1 | 0 | 0 | 0 | 0 |
| Bufonidae | *Bufo bankorensis* | 99.0 | 86.0 | 0.15 | M | 1.5 | 3725 | 0 | 0 | 0 | 0 | 0 | 0 | 0 |
| Bufonidae | *Bufo bufo* | - | - | - | - | 1.8 | 5332 | 1 | 0 | 1 | 0 | 0 | 0 | 0 |
| Bufonidae | *Bufo gargarizans* | 91.1 | 71.3 | 0.28 | M | 2.0 | 5000 | 0 | 0 | 0 | 0 | 0 | 0 | 0 |
| Bufonidae | *Bufo mauritanicus* | - | - | - | - | 1.5 | - | 0 | 0 | 0 | 0 | 0 | 0 | 0 |
| Bufonidae | *Bufo stejnegeri* | 54.6 | 55.0 | -0.01 | M | - | 830 | 0 | 0 | 0 | 0 | 0 | 0 | 0 |
| Bufonidae | *Bufo tibetanus* | 72.2 | 61.7 | 0.17 | M | 1.9 | 4000 | 0 | 0 | 0 | 0 | 0 | 0 | 0 |
| Bufonidae | *Bufo verrucosissimus* | 141.0 | 83.5 | 0.69 | R | 2.2 | 5685 | 0 | 0 | 0 | 0 | 0 | 0 | 0 |
| Bufonidae | *Capensibufo rosei* | 39.0 | 28.0 | 0.39 | R | 2.6 | - | - | - | - | - | 0 | 0 | 0 |
| Bufonidae | *Capensibufo tradouwi* | - | - | - | - | 2.0 | - | - | - | - | - | 0 | 0 | 0 |
| Bufonidae | *Dendrophryniscus minutus* | 20.5 | 16.5 | 0.24 | R | 1.0 | 160 | - | - | - | - | 0 | 0 | 0 |
| Bufonidae | *Duttaphrynus himalayanus* | 95.8 | 87.8 | 0.09 | M | 1.5 | - | - | - | - | - | 0 | 0 | 0 |
| Bufonidae | *Duttaphrynus melanostictus* | 75.2 | 68.5 | 0.10 | M | 1.4 | - | 0 | 0 | 0 | 0 | 0 | 0 | 0 |
| Bufonidae | *Incilius alvarius* | - | - | - | - | 1.4 | 7750 | 1 | 0 | 1 | 0 | 0 | 0 | 0 |
| Bufonidae | *Incilius coccifer* | 67.7 | 51.4 | 0.32 | M | 1.3 | 3000 | 0 | 0 | 0 | 0 | 0 | 0 | 0 |
| Bufonidae | *Incilius coniferus* | - | - | - | - | 1.8 | - | 0 | 0 | 0 | 0 | 0 | 0 | 0 |
| Bufonidae | *Incilius fastidiosus* | - | - | - | - | 4.3 | 85 | 1 | 0 | 1 | 0 | 0 | 0 | 0 |
| Bufonidae | *Incilius ibarrai* | 78.4 | 62.2 | 0.26 | M | - | - | - | - | - | - | - | - | - |
| Bufonidae | *Incilius luetkenii* | - | - | - | - | 1.5 | 3300 | 0 | 0 | 0 | 0 | 0 | 0 | 0 |
| Bufonidae | *Incilius melanochlorus* | - | - | - | - | - | - | 0 | 0 | 0 | 0 | 0 | 0 | 0 |
| Bufonidae | *Incilius nebulifer* | 52.0 | 44.2 | 0.18 | M | 1.0 | 9000 | 0 | 0 | 0 | 0 | 0 | 0 | 0 |
| Bufonidae | *Incilius valliceps* | - | - | - | - | 1.2 | - | 0 | 0 | 0 | 0 | 0 | 0 | 0 |
| Bufonidae | *Ingerophrynus biporcatus* | 59.8 | 49.0 | 0.22 | M | - | - | 0 | 0 | 0 | 0 | 0 | 0 | 0 |
| Bufonidae | *Mertensophryne taitana* | 28.5 | 23.4 | 0.22 | R | 2.5 | - | 0 | 0 | 0 | 0 | - | - | - |
| Bufonidae | *Nannophryne variegata* | - | - | - | - | - | 138 | 0 | 0 | 0 | 0 | 0 | 0 | 0 |
| Bufonidae | *Peltophryne fustiger* | 154.7 | 131 | 0.18 | R | 2.0 | - | 0 | 0 | 0 | 0 | 0 | 0 | 0 |
| Bufonidae | *Peltophryne peltocephala* | 134.1 | 120.9 | 0.11 | M | - | - | 0 | 0 | 0 | 0 | - | - | - |
| Bufonidae | *Phrynoidis asper* | 102.7 | 83.0 | 0.24 | M | 1.3 | 12792 | 0 | 0 | 0 | 0 | 0 | 0 | 0 |
| Bufonidae | *Phrynoidis juxtaspera* | - | - | - | - | - | - | 0 | 0 | 0 | 0 | - | - | - |
| Bufonidae | *Pseudepidalea pewzowi* | 80.3 | 72.2 | 0.11 | M | 1.3 | 3250 | 0 | 0 | 0 | 0 | 0 | 0 | 0 |
| Bufonidae | *Pseudepidalea viridis* | - | - | - | - | 1.7 | 11000 | 0 | 0 | 0 | 0 | 0 | 0 | 0 |
| Bufonidae | *Rhaebo glaberrimus* | 80.0 | 80.0 | 0.00 | R | - | - | 0 | 0 | 0 | 0 | - | - | - |
| Bufonidae | *Rhaebo haematiticus* | - | - | - | - | - | - | 0 | 0 | 0 | 0 | 0 | 0 | 0 |
| Bufonidae | *Rhinella arenarum* | - | - | - | - | - | 4500 | 0 | 0 | 0 | 0 | 0 | 0 | 0 |
| Bufonidae | *Rhinella castaneotica* | - | - | - | - | 1.5 | 178 | 0 | 0 | 0 | 0 | 0 | 0 | 0 |
| Bufonidae | *Rhinella chavin* | 59.5 | 50.2 | 0.19 | M | 2.4 | 276 | 0 | 0 | 0 | 0 | 0 | 0 | 0 |
| Bufonidae | *Rhinella dapsilis* | 77.0 | 65.0 | 0.18 | R | - | - | 0 | 0 | 0 | 0 | - | - | - |
| Bufonidae | *Rhinella margaritifera* | 74.0 | 51.8 | 0.43 | M | 2.0 | 1537 | 1 | 0 | 1 | 0 | 0 | 0 | 0 |
| Bufonidae | *Rhinella marina* | 118.4 | 96.0 | 0.23 | M | 1.9 | 35000 | 1 | 0 | 1 | 0 | 0 | 0 | 0 |
| Bufonidae | *Rhinella schneideri* | - | - | - | - | 1.8 | - | 0 | 0 | 0 | 0 | 0 | 0 | 0 |
| Bufonidae | *Rhinella spinulosa* | - | - | - | - | - | - | 0 | 0 | 0 | 0 | 0 | 0 | 0 |
| Bufonidae | *Schismaderma carens* | - | - | - | - | 2.0 | 6264 | 0 | 0 | 0 | 0 | 0 | 0 | 0 |
| Centrolenidae | *Centrolene geckoideum* | 62.0 | 74.5 | -0.17 | M | 3.8 | 112 | 0 | 0 | 0 | 0 | 1 | 0 | 1 |
| Centrolenidae | *Centrolenella prosoblepon* | 26.4 | 24.1 | 0.10 | M | - | 20 | 1 | 0 | 0 | 1 | 0 | 1 | 1 |
| Centrolenidae | *Cochranella euknemos* | 31.0 | 25.5 | 0.22 | R | - | 55 | 0 | 0 | 0 | 0 | 0 | 0 | 0 |
| Centrolenidae | *Cochranella granulosa* | 30.5 | 25.8 | 0.18 | R | 1.5 | 55 | 1 | 0 | 0 | 1 | 0 | 0 | 0 |
| Centrolenidae | *Hyalinobatrachium chirripoi* | 29.0 | 25.5 | 0.14 | R | - | 70 | 0 | 0 | 0 | 0 | 1 | 0 | 1 |
| Centrolenidae | *Hyalinobatrachium colymbiphyllum* | 26.8 | 25.0 | 0.07 | R | 1.5 | 50 | 1 | 0 | 0 | 1 | 1 | 0 | 1 |
| Centrolenidae | *Hyalinobatrachium fleischmanni* | 27.5 | 22.4 | 0.23 | R | 2.0 | 30 | 0 | 0 | 0 | 0 | 1 | 0 | 1 |
| Centrolenidae | *Hyalinobatrachium talamancae* | 25.5 | 23.5 | 0.09 | R | - | 28 | 0 | 0 | 0 | 0 | 1 | 0 | 1 |
| Centrolenidae | *Hyalinobatrachium valerioi* | 24.3 | 21.8 | 0.11 | R | 2.0 | 35 | 1 | 0 | 0 | 1 | 1 | 0 | 1 |
| Centrolenidae | *Nymphargus griffithsi* | 23.4 | 24.1 | -0.03 | M | - | - | 1 | 0 | 0 | 1 | 0 | 0 | 0 |
| Centrolenidae | *Sachatamia albomaculata* | 27.0 | 24.8 | 0.09 | R | - | - | 0 | 0 | 0 | 0 | - | - | - |
| Centrolenidae | *Sachatamia ilex* | 32.5 | 28.5 | 0.14 | R | - | 20 | 1 | 0 | 0 | 1 | - | - | - |
| Centrolenidae | *Teratohyla midas* | 22.7 | 18.4 | 0.23 | M | 1.5 | 28 | - | - | - | - | 0 | 0 | 0 |
| Centrolenidae | *Teratohyla pulverata* | 29.5 | 26.0 | 0.13 | R | - | 42 | 0 | 0 | 0 | 0 | 0 | 0 | 0 |
| Centrolenidae | *Teratohyla spinosa* | 21.5 | 18.9 | 0.14 | R | - | 20 | 0 | 0 | 0 | 0 | - | - | - |
| Ceratobatrachidae | *Ingerana baluensis* | - | - | - | - | 2.4 | - | - | - | - | - | - | - | - |
| Craugastoridae | *Craugastor augusti* | - | - | - | - | - | 59 | 0 | 0 | 0 | 0 | 1 | 0 | 1 |
| Craugastoridae | *Haddadus binotatus* | - | - | - | - | - | - | - | - | - | - | 0 | 0 | 0 |
| Cycloramphidae | *Rhinoderma darwinii* | - | - | - | - | 3.6 | 40 | 0 | 0 | 0 | 0 | 1 | 0 | 1 |
| Dendrobatidae | *Adelphobates galactonotus* | 35.7 | 33.2 | 0.08 | M | - | - | - | - | - | - | - | - | - |
| Dendrobatidae | *Adelphobates quinquevittatus* | 17.3 | 16.6 | 0.04 | M | - | - | 0 | 0 | 0 | 0 | 1 | 0 | 1 |
| Dendrobatidae | *Ameerega hahneli* | 20.5 | 18.0 | 0.14 | R | 2.3 | 22 | 0 | 0 | 0 | 0 | 1 | 0 | 1 |
| Dendrobatidae | *Ameerega parvula* | 21.2 | 19.0 | 0.12 | M | 3.0 | 8 | 0 | 0 | 0 | 0 | 1 | 0 | 1 |
| Dendrobatidae | *Ameerega petersi* | 28.0 | 24.8 | 0.13 | M | - | - | 0 | 0 | 0 | 0 | 1 | 0 | 1 |
| Dendrobatidae | *Ameerega picta* | 24.4 | 23.2 | 0.05 | M | 2.0 | 18 | 0 | 0 | 0 | 0 | 1 | 1 | 1 |
| Dendrobatidae | *Ameerega pulchripecta* | 26.0 | 22.6 | 0.15 | M | - | - | - | - | - | - | - | - | - |
| Dendrobatidae | *Ameerega silverstonei* | 41.8 | 35.9 | 0.17 | M | 2.0 | 30 | 0 | 0 | 0 | 0 | 1 | 0 | 1 |
| Dendrobatidae | *Ameerega trivittata* | 43.6 | 37.9 | 0.15 | M | 3.2 | 50 | 1 | 1 | 0 | 1 | 1 | 0 | 1 |
| Dendrobatidae | *Colostethus inguinalis* | - | - | - | - | - | - | 1 | 1 | 0 | 1 | 0 | 1 | 1 |
| Dendrobatidae | *Dendrobates auratus* | 33.7 | 30.3 | 0.11 | M | 2.0 | 8 | 1 | 1 | 1 | 1 | 1 | 0 | 1 |
| Dendrobatidae | *Dendrobates leucomelas* | 35.1 | 32.7 | 0.07 | M | 3.9 | 5 | 1 | 0 | 1 | 0 | 1 | 0 | 1 |
| Dendrobatidae | *Dendrobates tinctorius* | 46.5 | 41.0 | 0.13 | M | - | 5 | 0 | 0 | 0 | 0 | 1 | 0 | 1 |
| Dendrobatidae | *Dendrobates truncatus* | 28.7 | 25.6 | 0.12 | M | - | 5 | 0 | 0 | 0 | 0 | 1 | 0 | 1 |
| Dendrobatidae | *Epipedobates anthonyi* | 20.0 | 18.1 | 0.10 | M | - | - | 0 | 0 | 0 | 0 | 1 | 0 | 1 |
| Dendrobatidae | *Epipedobates boulengeri* | 18.5 | 16.9 | 0.09 | M | - | - | 0 | 0 | 0 | 0 | 1 | 0 | 1 |
| Dendrobatidae | *Epipedobates espinosai* | - | - | - | - | - | - | 0 | 0 | 0 | 0 | 1 | 0 | 1 |
| Dendrobatidae | *Epipedobates tricolor* | 23.3 | 21.0 | 0.11 | M | - | 22 | 1 | 0 | 0 | 1 | 1 | 0 | 1 |
| Dendrobatidae | *Hyloxalus awa* | 22.5 | 19.3 | 0.17 | R | - | 21 | 1 | 0 | 0 | 1 | 1 | 0 | 1 |
| Dendrobatidae | *Hyloxalus elachyhistus* | 21.2 | 18.3 | 0.16 | M | - | 19 | 0 | 0 | 0 | 0 | 1 | 0 | 1 |
| Dendrobatidae | *Hyloxalus idiomelus* | 25.1 | 22.0 | 0.14 | M | - | - | - | - | - | - | - | - | - |
| Dendrobatidae | *Hyloxalus insulatus* | 18.3 | 21.3 | -0.14 | M | - | - | - | - | - | - | - | - | - |
| Dendrobatidae | *Hyloxalus nexipus* | 21.4 | 19.3 | 0.11 | M | - | - | - | - | - | - | - | - | - |
| Dendrobatidae | *Hyloxalus subpunctatus* | 24.3 | 21.4 | 0.14 | M | 1.5 | 21 | 0 | 0 | 0 | 0 | 1 | 0 | 1 |
| Dendrobatidae | *Hyloxalus sylvaticus* | 28.6 | 23.7 | 0.20 | M | - | - | - | - | - | - | - | - | - |
| Dendrobatidae | *Hyloxalus vertebralis* | 17.9 | 16.1 | 0.11 | M | - | - | 0 | 0 | 0 | 0 | 1 | 0 | 1 |
| Dendrobatidae | *Oophaga granulifera* | 20.8 | 20.7 | 0.00 | M | - | 3 | 1 | 0 | 1 | 1 | 1 | 1 | 1 |
| Dendrobatidae | *Oophaga histrionica* | 32.7 | 33.0 | -0.01 | M | 1.5 | - | 1 | 1 | 1 | 0 | 0 | 1 | 1 |
| Dendrobatidae | *Oophaga lehmanni* | 32.9 | 33.4 | -0.02 | M | 1.5 | - | 1 | 0 | - | - | 0 | 1 | 1 |
| Dendrobatidae | *Oophaga pumilio* | 20.7 | 20.9 | -0.01 | M | 1.1 | 3 | 1 | 1 | 0 | 1 | 1 | 1 | 1 |
| Dendrobatidae | *Oophaga speciosa* | 29.1 | 28.4 | 0.02 | M | - | - | 1 | 1 | - | - | 0 | 1 | 1 |
| Dendrobatidae | *Phyllobates aurotaenia* | 30.4 | 26.9 | 0.13 | M | - | - | - | - | - | - | - | - | - |
| Dendrobatidae | *Phyllobates bicolor* | 39.2 | 36.2 | 0.08 | M | - | 17 | 0 | 0 | 0 | 0 | 1 | 0 | 1 |
| Dendrobatidae | *Phyllobates lugubris* | 22.2 | 19.2 | 0.16 | M | - | 18 | 0 | 0 | 0 | 0 | 1 | 0 | 1 |
| Dendrobatidae | *Phyllobates terribilis* | 43.2 | 41.1 | 0.05 | M | 2.5 | 15 | 0 | 1 | 0 | 0 | 1 | 0 | 1 |
| Dendrobatidae | *Phyllobates vittatus* | 27.7 | 24.4 | 0.14 | M | 2.5 | 19 | 1 | 1 | 1 | 1 | 1 | 0 | 1 |
| Dendrobatidae | *Ranitomeya fantastica* | - | - | - | - | - | - | 0 | 0 | 0 | 0 | 1 | 1 | 1 |
| Dendrobatidae | *Ranitomeya fulgurita* | 15.2 | 14.5 | 0.05 | M | - | - | - | - | - | - | - | - | - |
| Dendrobatidae | *Ranitomeya imitator* | 18.2 | 17.4 | 0.05 | M | - | 2 | 0 | 0 | 0 | 0 | 1 | 1 | 1 |
| Dendrobatidae | *Ranitomeya minuta* | 13.5 | 13.2 | 0.02 | M | - | - | 0 | 0 | 0 | 0 | 1 | 0 | 1 |
| Dendrobatidae | *Ranitomeya reticulata* | 15.1 | 14.4 | 0.05 | M | 2.0 | 2 | 0 | 0 | 0 | 0 | 1 | 0 | 1 |
| Dendrobatidae | *Ranitomeya vanzolinii* | 17.9 | 17.8 | 0.01 | R | - | - | 0 | 0 | 0 | 0 | 1 | 1 | 1 |
| Dendrobatidae | *Ranitomeya variabilis* | 18.0 | 17.4 | 0.03 | M | - | 6 | 1 | 0 | 1 | 0 | 1 | 0 | 1 |
| Dendrobatidae | *Ranitomeya ventrimaculata* | 17.0 | 16.0 | 0.06 | R | 2.0 | 2 | 1 | 0 | 0 | 1 | 1 | 1 | 1 |
| Dendrobatidae | *Silverstoneia flotator* | 16.4 | 15.5 | 0.06 | R | - | - | 1 | 0 | 0 | 1 | 1 | 0 | 1 |
| Dendrobatidae | *Silverstoneia nubicola* | 20.3 | 18.0 | 0.13 | R | - | - | 0 | 0 | 0 | 0 | 1 | 0 | 1 |
| Dicroglossidae | *Euphlyctis cyanophlyctis* | 48.0 | 67.0 | -0.28 | M | - | - | 0 | 0 | 0 | 0 | 0 | 0 | 0 |
| Dicroglossidae | *Fejervarya cancrivora* | 68.6 | 58.8 | 0.17 | M | 1.6 | - | 0 | 0 | 0 | 0 | 0 | 0 | 0 |
| Dicroglossidae | *Fejervarya limnocharis* | 48.8 | 39.0 | 0.25 | M | 1.0 | 1560 | 1 | 0 | 1 | 0 | 0 | 0 | 0 |
| Dicroglossidae | *Hoplobatrachus occipitalis* | - | - | - | - | 5.0 | - | 0 | 0 | 0 | 0 | 0 | 0 | 0 |
| Dicroglossidae | *Hoplobatrachus rugulosus* | 71.7 | 82.4 | -0.13 | M | 1.8 | 2000 | 0 | 0 | 0 | 0 | 0 | 0 | 0 |
| Dicroglossidae | *Limnonectes blythii* | - | - | - | - | 2.1 | 1852 | 1 | 0 | 0 | 1 | 1 | 1 | 1 |
| Dicroglossidae | *Limnonectes finchi* | - | - | - | - | 2.0 | - | - | - | - | - | 1 | 0 | 1 |
| Dicroglossidae | *Limnonectes gyldenstolpei* | 52.9 | 58.5 | -0.10 | M | - | - | 0 | 0 | 0 | 0 | - | - | - |
| Dicroglossidae | *Limnonectes ibanorum* | - | - | - | - | - | 1122 | - | - | - | - | 0 | 0 | 0 |
| Dicroglossidae | *Limnonectes kuhlii* | 54.9 | 55.6 | -0.01 | M | 2.3 | 80 | 1 | 0 | 0 | 1 | 0 | 0 | 0 |
| Dicroglossidae | *Limnonectes laticeps* | 40.8 | 43.8 | -0.07 | M | - | - | 0 | 0 | 0 | 0 | - | - | - |
| Dicroglossidae | *Limnonectes macrodon* | - | - | - | - | 1.4 | 1660 | 0 | 0 | 0 | 0 | 0 | 0 | 0 |
| Dicroglossidae | *Limnonectes microdiscus* | 37.3 | 32.4 | 0.15 | M | 4.5 | 53 | - | - | - | - | 1 | 0 | 1 |
| Dicroglossidae | *Limnonectes palavanensis* | - | - | - | - | 2.3 | - | - | - | - | - | 1 | 0 | 1 |
| Dicroglossidae | *Limnonectes paramacrodon* | - | - | - | - | - | - | 0 | 0 | 0 | 0 | - | - | - |
| Dicroglossidae | *Limnonectes parvus* | - | - | - | - | 2.0 | 11 | - | - | - | - | - | - | - |
| Dicroglossidae | *Nannophrys ceylonensis* | 48.7 | 46.6 | 0.05 | M | - | - | 1 | 0 | 0 | 1 | 1 | 1 | **1** |
| Dicroglossidae | *Nanorana parkeri* | 49.4 | 45.7 | 0.08 | M | 2.0 | - | 0 | 0 | 0 | 0 | 0 | 0 | 0 |
| Dicroglossidae | *Nanorana pleskei* | 37.3 | 36.6 | 0.02 | M | 1.9 | 50 | 0 | 0 | 0 | 0 | 0 | 0 | 0 |
| Dicroglossidae | *Nanorana unculuanus* | 77.9 | 74.6 | 0.04 | M | 4.0 | 97 | 0 | 0 | 0 | 0 | 0 | 0 | 0 |
| Dicroglossidae | *Nanorana yunnanensis* | 95.0 | 95.5 | -0.01 | M | 3.5 | 633 | 0 | 0 | 0 | 0 | 0 | 0 | 0 |
| Dicroglossidae | *Occidozyga laevis* | 32.6 | 24.4 | 0.34 | M | - | - | - | - | - | - | - | - | - |
| Dicroglossidae | *Occidozyga lima* | 27.6 | 21.2 | 0.31 | M | 1.0 | 707 | 0 | 0 | 0 | 0 | - | - | - |
| Dicroglossidae | *Occidozyga martensii* | 25.0 | 20.4 | 0.22 | M | 1.0 | - | 0 | 0 | 0 | 0 | - | - | - |
| Dicroglossidae | *Quasipaa boulengeri* | 103.3 | 100.6 | 0.03 | M | 3.9 | - | 0 | 0 | 0 | 0 | 0 | 0 | 0 |
| Dicroglossidae | *Quasipaa spinosa* | 93.2 | 89.2 | 0.05 | M | 4.3 | 228 | 0 | 0 | 0 | 0 | 0 | 0 | 0 |
| Dicroglossinae | *Feirana taihangnica* | 80.8 | 66.9 | 0.21 | M | 3.5 | - | - | - | - | - | 0 | 0 | 0 |
| Dicroglossinae | *Limnonectes fujianensis* | 47.5 | 53.9 | -0.12 | M | 2.3 | 53 | 0 | 0 | 0 | 0 | 0 | 0 | 0 |
| Dicroglossinae | *Nanorana ventripunctata* | 49.4 | 44.1 | 0.12 | M | 2.1 | 290 | - | - | - | - | 0 | 0 | 0 |
| Dicroglossinae | *Paa conaensis* | 55.2 | 58.0 | -0.05 | M | 3.3 | - | - | - | - | - | 0 | 0 | 0 |
| Dicroglossinae | *Paa exilispinosa* | 57.1 | 61.2 | -0.07 | M | 3.3 | - | 0 | 0 | 0 | 0 | - | - | - |
| Dicroglossinae | *Paa jiulongensis* | 91.1 | 74.8 | 0.22 | M | - | - | 0 | 0 | 0 | 0 | - | - | - |
| Dicroglossinae | *Paa liebigii* | 83.1 | 77.9 | 0.07 | M | 3.0 | - | - | - | - | - | 0 | 0 | 0 |
| Dicroglossinae | *Paa liui* | 61.2 | 64.2 | -0.05 | M | - | - | 0 | 0 | 0 | 0 | - | - | - |
| Dicroglossinae | *Paa robertingeri* | 86.3 | 87.3 | -0.01 | M | 3.7 | - | 0 | 0 | 0 | 0 | - | - | - |
| Dicroglossinae | *Paa shini* | 94.9 | 98.6 | -0.04 | M | 3.5 | 10 | - | - | - | - | 0 | 0 | 0 |
| Dicroglossinae | *Yerana yei* | 75.0 | 58.1 | 0.29 | M | - | - | - | - | - | - | 0 | 0 | 0 |
| Eleutherodactylidae | *Diasporus diastema* | 21.0 | 18.5 | 0.14 | R | 4.3 | 16 | 0 | 1 | 0 | 0 | 0 | 0 | 0 |
| Eleutherodactylidae | *Eleutherodactylus coqui* | 33.4 | 33.4 | 0.00 | M | - | 28 | 1 | 1 | 0 | 1 | 1 | 0 | 1 |
| Eleutherodactylidae | *Eleutherodactylus cundalli* | 41.7 | 32.3 | 0.29 | M | 4.2 | 48 | 0 | 0 | 0 | 0 | 0 | 1 | 1 |
| Eleutherodactylidae | *Eleutherodactylus johnstonei* | 26.9 | 23.1 | 0.16 | M | 3.0 | 20 | 1 | 0 | 0 | 1 | 1 | 1 | 1 |
| Eleutherodactylidae | *Eleutherodactylus planirostris* | - | - | - | - | - | 14 | 0 | 0 | 0 | 0 | 0 | 0 | 0 |
| Hemiphractidae | *Hemiphractus helioi* | - | - | - | - | - | - | 0 | 0 | 0 | 0 | 0 | 1 | 1 |
| Hemiphractidae | *Stefania evansi* | 63.9 | 45.8 | 0.40 | M | 1.0 | 23 | 0 | 0 | 0 | 0 | 0 | 1 | 1 |
| Hemisotidae | *Hemisus marmoratum* | 31.8 | 27.9 | 0.14 | R | 2.0 | 200 | 0 | 0 | 0 | 0 | 0 | 1 | 1 |
| Hylidae | *Acris crepitans* | 24.2 | 22.8 | 0.06 | M | 1.5 | 500 | 1 | 0 | 0 | 1 | 0 | 0 | 0 |
| Hylidae | *Acris gryllus* | 24.5 | 22.0 | 0.11 | R | 1.1 | 232 | 0 | 0 | 0 | 0 | 0 | 0 | 0 |
| Hylidae | *Agalychnis callidryas* | 62.5 | 48.2 | 0.30 | M | 3.2 | 265 | 1 | 0 | 0 | 1 | 0 | 0 | 0 |
| Hylidae | *Agalychnis litodryas* | - | - | - | - | - | - | 0 | 0 | 0 | 0 | 0 | 0 | 0 |
| Hylidae | *Agalychnis saltator* | 57.0 | 43.3 | 0.32 | R | 5.0 | 46 | 1 | 0 | 1 | 0 | 0 | 0 | 0 |
| Hylidae | *Agalychnis spurrelli* | 75.0 | 62.0 | 0.21 | M | - | - | 0 | 0 | 0 | 0 | 0 | 0 | 0 |
| Hylidae | *Anotheca spinosa* | 63.4 | 61.1 | 0.04 | M | 1.6 | 316 | 0 | 0 | 0 | 0 | 0 | 1 | 1 |
| Hylidae | *Aplastodiscus albofrenatus* | - | - | - | - | - | - | 0 | 0 | 0 | 0 | - | - | - |
| Hylidae | *Aplastodiscus perviridis* | - | - | - | - | 1.8 | 227 | 0 | 0 | 0 | 0 | 1 | 0 | 1 |
| Hylidae | *Bokermannohyla circumdata* | 63.0 | 63.0 | 0.00 | R | - | - | 0 | 0 | 0 | 0 | - | - | - |
| Hylidae | *Bokermannohyla martinsi* | 56.0 | 61.5 | -0.09 | R | - | - | 0 | 0 | 0 | 0 | - | - | - |
| Hylidae | *Bromeliohyla bromeliacia* | 32.4 | 27.0 | 0.20 | R | - | 14 | 0 | 0 | 0 | 0 | 0 | 1 | 1 |
| Hylidae | *Charadrahyla taeniopus* | 64.2 | 58.0 | 0.11 | M | 2.2 | - | 0 | 0 | 0 | 0 | 0 | 0 | 0 |
| Hylidae | *Cruziohyla calcarifer* | 65.0 | 52.0 | 0.25 | M | 3.8 | 80 | 0 | 0 | 0 | 0 | 0 | 0 | 0 |
| Hylidae | *Dendropsophus allenorum* | 26.0 | 19.0 | 0.37 | R | - | - | - | - | - | - | 0 | 0 | 0 |
| Hylidae | *Dendropsophus anceps* | 40.3 | 37.0 | 0.09 | M | 1.0 | - | 0 | 0 | 0 | 0 | 0 | 0 | 0 |
| Hylidae | *Dendropsophus aperomeus* | 25.0 | 19.8 | 0.26 | M | - | - | - | - | - | - | - | - | - |
| Hylidae | *Dendropsophus bifurcus* | 31.8 | 25.1 | 0.27 | M | 1.5 | 186 | 0 | 0 | 0 | 0 | 0 | 0 | 0 |
| Hylidae | *Dendropsophus bipunctatus* | 25.5 | 16.5 | 0.55 | R | - | - | 0 | 0 | 0 | 0 | - | - | - |
| Hylidae | *Dendropsophus brevifrons* | 21.4 | 18.5 | 0.16 | M | 1.0 | 79 | 0 | 0 | 0 | 0 | 0 | 0 | 0 |
| Hylidae | *Dendropsophus ebraccatus* | 36.5 | 25.1 | 0.45 | R | 1.3 | - | 1 | 0 | 0 | 1 | 0 | 0 | 0 |
| Hylidae | *Dendropsophus elegans* | - | - | - | - | - | - | 1 | 0 | 0 | 1 | 0 | 0 | 0 |
| Hylidae | *Dendropsophus koechlini* | 26.0 | 20.5 | 0.27 | R | - | - | 0 | 0 | 0 | 0 | 0 | 0 | 0 |
| Hylidae | *Dendropsophus leucophyllatus* | 42.0 | 33.9 | 0.24 | M | 1.1 | 587 | 1 | 0 | 0 | 1 | 0 | 0 | 0 |
| Hylidae | *Dendropsophus marmoratus* | 49.3 | 39.1 | 0.26 | M | 1.5 | 979 | - | - | - | - | 0 | 0 | 0 |
| Hylidae | *Dendropsophus microcephalus* | 26.8 | 22.8 | 0.17 | M | 1.0 | 180 | 0 | 0 | 0 | 0 | 0 | 0 | 0 |
| Hylidae | *Dendropsophus minusculus* | 23.3 | 20.0 | 0.17 | M | - | - | 0 | 0 | 0 | 0 | - | - | - |
| Hylidae | *Dendropsophus minutus* | 23.8 | 21.3 | 0.12 | M | 1.0 | 211 | 0 | 0 | 0 | 0 | 0 | 0 | 0 |
| Hylidae | *Dendropsophus miyatai* | 25.0 | 20.0 | 0.25 | R | - | - | 0 | 0 | 0 | 0 | 0 | 0 | 0 |
| Hylidae | *Dendropsophus nanus* | 20.5 | 18.5 | 0.11 | M | 1.2 | 100 | - | - | - | - | 0 | 0 | 0 |
| Hylidae | *Dendropsophus parviceps* | 23.4 | 16.8 | 0.39 | M | 1.0 | 234 | 0 | 0 | 0 | 0 | 0 | 0 | 0 |
| Hylidae | *Dendropsophus pelidna* | 38.5 | 34.4 | 0.12 | R | - | - | 0 | 0 | 0 | 0 | - | - | - |
| Hylidae | *Dendropsophus rhodopeplus* | 26.7 | 20.7 | 0.29 | M | 1.0 | 285 | - | - | - | - | 0 | 0 | 0 |
| Hylidae | *Dendropsophus riveroi* | 22.8 | 18.5 | 0.23 | M | - | - | 0 | 0 | 0 | 0 | - | - | - |
| Hylidae | *Dendropsophus robertmertensi* | 26.6 | 24.7 | 0.08 | M | - | - | 0 | 0 | 0 | 0 | - | - | - |
| Hylidae | *Dendropsophus rubicundulus* | 22.0 | 20.0 | 0.10 | R | - | - | 0 | 0 | 0 | 0 | - | - | - |
| Hylidae | *Dendropsophus sanborni* | 18.5 | 16.0 | 0.16 | R | 0.9 | 60 | 0 | 0 | 0 | 0 | 0 | 0 | 0 |
| Hylidae | *Dendropsophus sarayacuensis* | 33.5 | 25.0 | 0.34 | M | 2.0 | 113 | 0 | 0 | 0 | 0 | 0 | 0 | 0 |
| Hylidae | *Dendropsophus sartori* | 28.6 | 24.8 | 0.15 | R | - | - | 0 | 0 | 0 | 0 | - | - | - |
| Hylidae | *Dendropsophus seniculus* | 39.5 | 32.5 | 0.22 | R | - | - | 0 | 0 | 0 | 0 | - | - | - |
| Hylidae | *Dendropsophus triangulum* | 35.4 | 25.4 | 0.40 | M | 1.5 | 501 | 0 | 0 | 0 | 0 | 0 | 0 | 0 |
| Hylidae | *Duellmanohyla rufioculis* | 36.7 | 28.2 | 0.30 | M | 2.4 | 112 | 0 | 0 | 0 | 0 | 0 | 0 | 0 |
| Hylidae | *Ecnomiohyla miliaria* | 69.7 | 81.6 | -0.15 | R | - | - | 1 | 0 | 0 | 1 | - | - | - |
| Hylidae | *Ecnomiohyla miotympanum* | 39.7 | 28.5 | 0.39 | M | 2.4 | 120 | 0 | 0 | 0 | 0 | 0 | 0 | 0 |
| Hylidae | *Exerodonta melanomma* | 30.1 | 27.1 | 0.11 | M | - | - | 0 | 0 | 0 | 0 | - | - | - |
| Hylidae | *Exerodonta sumichrasti* | 30.2 | 26.2 | 0.15 | M | 1.6 | - | 0 | 0 | 0 | 0 | 0 | 0 | 0 |
| Hylidae | *Hyla andersonii* | 42.5 | 35.5 | 0.20 | R | 1.3 | 500 | 0 | 0 | 0 | 0 | 0 | 0 | 0 |
| Hylidae | *Hyla annectans* | 38.8 | 34.2 | 0.14 | M | - | - | 0 | 0 | 0 | 0 | - | - | - |
| Hylidae | *Hyla arborea* | 33.0 | 24.8 | 0.33 | M | 1.2 | 1282 | 0 | 0 | 0 | 0 | 0 | 0 | 0 |
| Hylidae | *Hyla arenicolor* | 47.2 | 35.3 | 0.34 | M | 2.1 | - | 0 | 0 | 0 | 0 | 0 | 0 | 0 |
| Hylidae | *Hyla avivoca* | 40.5 | 33.5 | 0.21 | R | 1.2 | 632 | 1 | 0 | 0 | 1 | 0 | 0 | 0 |
| Hylidae | *Hyla chinensis* | 34.2 | 30.6 | 0.12 | M | 1.3 | 459 | 0 | 0 | 0 | 0 | 0 | 0 | 0 |
| Hylidae | *Hyla chrysoscelis* | 42.0 | 39.5 | 0.06 | R | 1.2 | 2000 | 1 | 0 | - | - | 0 | 0 | 0 |
| Hylidae | *Hyla cinerea* | 48.9 | 53.1 | -0.08 | M | 1.2 | 875 | 1 | 0 | 0 | 1 | 0 | 0 | 0 |
| Hylidae | *Hyla euphorbiacea* | 36.4 | 34.7 | 0.05 | M | - | - | 0 | 0 | 0 | 0 | 0 | 0 | 0 |
| Hylidae | *Hyla eximia* | 28.5 | 27.8 | 0.03 | M | - | - | 0 | 0 | 0 | 0 | 0 | 0 | 0 |
| Hylidae | *Hyla femoralis* | 31.5 | 30.5 | 0.03 | R | 0.9 | - | 0 | 0 | 0 | 0 | 0 | 0 | 0 |
| Hylidae | *Hyla gratiosa* | 59.0 | 58.5 | 0.01 | R | 1.9 | - | 0 | 0 | 0 | 0 | 0 | 0 | 0 |
| Hylidae | *Hyla japonica* | 28.0 | 32.7 | -0.14 | M | 1.3 | 920 | 0 | 0 | 0 | 0 | 0 | 0 | 0 |
| Hylidae | *Hyla meridionalis* | - | - | - | - | 1.3 | - | - | - | - | - | 0 | 0 | 0 |
| Hylidae | *Hyla plicata* | 43.8 | 39.7 | 0.10 | M | - | - | 0 | 0 | 0 | 0 | - | - | - |
| Hylidae | *Hyla savignyi* | - | - | - | - | 1.3 | 600 | 0 | 0 | 0 | 0 | 0 | 0 | 0 |
| Hylidae | *Hyla squirella* | 30.0 | 29.5 | 0.02 | R | 0.9 | 950 | 1 | 0 | - | - | 0 | 0 | 0 |
| Hylidae | *Hyla tsinlingensis* | 44.1 | 40.5 | 0.09 | M | 1.3 | - | 0 | 0 | 0 | 0 | - | - | - |
| Hylidae | *Hyla versicolor* | 46.5 | 41.5 | 0.12 | R | 1.2 | 1800 | 1 | 0 | 0 | 1 | 0 | 0 | 0 |
| Hylidae | *Hyla walkeri* | 31.6 | 32.0 | -0.01 | M | - | - | 0 | 0 | 0 | 0 | - | - | - |
| Hylidae | *Hyla wrightorum* | 36.0 | 34.0 | 0.06 | R | 1.2 | - | 0 | 0 | 0 | 0 | 0 | 0 | 0 |
| Hylidae | *Hylomantis lemur* | 42.6 | 33.8 | 0.26 | M | 3.3 | 45 | 1 | 0 | 1 | 0 | 0 | 0 | 0 |
| Hylidae | *Hyloscirtus armatus* | 64.3 | 59.6 | 0.08 | M | - | - | 0 | 0 | 0 | 0 | - | - | - |
| Hylidae | *Hyloscirtus colymba* | 36.2 | 34.7 | 0.05 | M | - | - | 0 | 0 | 0 | 0 | - | - | - |
| Hylidae | *Hyloscirtus palmeri* | 43.0 | 40.5 | 0.06 | R | - | - | 0 | 0 | 0 | 0 | 0 | 0 | 0 |
| Hylidae | *Hyloscirtus phyllognathus* | 36.9 | 33.0 | 0.12 | M | 3.1 | - | 0 | 0 | 0 | 0 | 0 | 0 | 0 |
| Hylidae | *Hyloscirtus simmonsi* | 44.3 | 36.6 | 0.21 | R | - | - | - | - | - | - | 0 | 0 | 0 |
| Hylidae | *Hypsiboas albomarginatus* | 57.3 | 48.6 | 0.18 | M | - | - | 0 | 0 | 0 | 0 | - | - | - |
| Hylidae | *Hypsiboas albopunctatus* | 60.5 | 50.0 | 0.21 | R | 1.7 | - | 0 | 0 | 0 | 0 | - | - | - |
| Hylidae | *Hypsiboas andinus* | 49.8 | 46.5 | 0.07 | M | - | - | 0 | 0 | 0 | 0 | 0 | 0 | 0 |
| Hylidae | *Hypsiboas balzani* | 48.5 | 40.4 | 0.20 | M | - | - | 0 | 0 | 0 | 0 | - | - | - |
| Hylidae | *Hypsiboas bischoffi* | 54.5 | 39.0 | 0.40 | R | - | - | 0 | 0 | 0 | 0 | - | - | - |
| Hylidae | *Hypsiboas boans* | 102.1 | 101.0 | 0.01 | M | 2.0 | 3154 | 0 | 0 | 0 | 0 | 1 | 0 | 1 |
| Hylidae | *Hypsiboas calcaratus* | 53.9 | 35.9 | 0.50 | M | 1.5 | 1143 | - | - | - | - | 0 | 0 | 0 |
| Hylidae | *Hypsiboas cinerascens* | 41.2 | 39.0 | 0.06 | M | 1.5 | 426 | 0 | 0 | 0 | 0 | 0 | 0 | 0 |
| Hylidae | *Hypsiboas crepitans* | 65.5 | 53.9 | 0.22 | M | 1.5 | 1064 | 0 | 0 | 0 | 0 | 0 | 0 | 0 |
| Hylidae | *Hypsiboas faber* | - | - | - | - | 1.5 | - | 1 | 0 | 0 | 1 | 1 | 0 | 1 |
| Hylidae | *Hypsiboas fasciatus* | 46.2 | 35.6 | 0.30 | M | 1.5 | 569 | - | - | - | - | 0 | 0 | 0 |
| Hylidae | *Hypsiboas geographicus* | 66.6 | 44.2 | 0.51 | M | 1.0 | 2797 | 0 | 0 | 0 | 0 | 0 | 0 | 0 |
| Hylidae | *Hypsiboas guentheri* | 45.5 | 36.5 | 0.25 | R | - | - | 0 | 0 | 0 | 0 | - | - | - |
| Hylidae | *Hypsiboas heilprini* | 49.3 | 49.4 | 0.00 | M | - | - | 0 | 0 | 0 | 0 | 0 | 0 | 0 |
| Hylidae | *Hypsiboas lanciformis* | 87.0 | 74.9 | 0.16 | M | 2.0 | 1617 | - | - | - | - | 0 | 0 | 0 |
| Hylidae | *Hypsiboas lemai* | 34.7 | 29.6 | 0.17 | R | 2.5 | 54 | 0 | 0 | 0 | 0 | 0 | 0 | 0 |
| Hylidae | *Hypsiboas marginatus* | - | - | - | - | - | - | - | - | - | - | 0 | 0 | 0 |
| Hylidae | *Hypsiboas marianitae* | 51.4 | 47.6 | 0.08 | M | - | - | 0 | 0 | 0 | 0 | - | - | - |
| Hylidae | *Hypsiboas pardalis* | 70.0 | 60.0 | 0.17 | M | - | - | 1 | 0 | 0 | 1 | 1 | 0 | 1 |
| Hylidae | *Hypsiboas polytaenius* | - | - | - | - | - | 150 | 0 | 0 | 0 | 0 | 0 | 0 | 0 |
| Hylidae | *Hypsiboas prasinus* | - | - | - | - | - | - | 0 | 0 | 0 | 0 | - | - | - |
| Hylidae | *Hypsiboas pulchellus* | 49.5 | 45.8 | 0.08 | R | 1.5 | - | 0 | 0 | 0 | 0 | 0 | 0 | 0 |
| Hylidae | *Hypsiboas raniceps* | 63.5 | 54.0 | 0.18 | R | 1.4 | 2100 | 0 | 0 | 0 | 0 | 0 | 0 | 0 |
| Hylidae | *Hypsiboas rosenbergi* | 26.6 | 24.7 | 0.08 | R | 2.0 | 2400 | 1 | 0 | 0 | 1 | 1 | 0 | 1 |
| Hylidae | *Hypsiboas rufitelus* | 51.1 | 46.2 | 0.11 | R | 1.8 | - | 0 | 0 | 0 | 0 | 0 | 0 | 0 |
| Hylidae | *Hypsiboas semiguttatus* | 40.5 | 40.3 | 0.00 | R | - | - | 0 | 0 | 0 | 0 | - | - | - |
| Hylidae | *Hypsiboas sibleszi* | 35.0 | 32.3 | 0.08 | M | 2.3 | 42 | 0 | 0 | 0 | 0 | 0 | 0 | 0 |
| Hylidae | *Isthmohyla pseudopuma* | 44.3 | 39.7 | 0.12 | M | 1.7 | 2150 | 1 | 0 | 1 | 0 | 0 | 0 | 0 |
| Hylidae | *Isthmohyla rivularis* | 35.7 | 32.3 | 0.11 | M | 2.4 | 90 | 0 | 0 | 0 | 0 | 0 | 0 | 0 |
| Hylidae | *Isthmohyla tica* | 38.9 | 31.6 | 0.23 | M | 2.3 | 192 | 0 | 0 | 0 | 0 | 0 | 0 | 0 |
| Hylidae | *Isthmohyla zeteki* | 25.4 | 22.5 | 0.13 | M | - | 24 | 0 | 0 | 0 | 0 | 0 | 1 | 1 |
| Hylidae | *Itapotihyla langsdorffii* | 99.0 | 72.0 | 0.38 | R | 1.0 | - | 0 | 0 | 0 | 0 | - | - | - |
| Hylidae | *Litoria arfakiana* | 60.0 | 50.0 | 0.20 | R | - | - | 0 | 0 | 0 | 0 | - | - | - |
| Hylidae | *Litoria aurea* | 93.9 | 91.8 | 0.02 | R | 1.4 | - | 0 | 0 | 0 | 0 | 0 | 0 | 0 |
| Hylidae | *Litoria australis* | 97.0 | 87.0 | 0.11 | R | 1.6 | 550 | 0 | 0 | 0 | 0 | 0 | 0 | 0 |
| Hylidae | *Litoria brevipes* | 42.0 | 37.0 | 0.14 | R | 1.3 | - | 0 | 0 | 0 | 0 | 0 | 0 | 0 |
| Hylidae | *Litoria caerulea* | 84.0 | 72.1 | 0.17 | R | 1.3 | 1800 | 0 | 0 | 0 | 0 | 0 | 0 | 0 |
| Hylidae | *Litoria cheesmani* | 60.0 | 40.0 | 0.50 | R | - | - | 0 | 0 | 0 | 0 | - | - | - |
| Hylidae | *Litoria foricula* | 50.0 | 40.0 | 0.25 | R | - | - | 0 | 0 | 0 | 0 | - | - | - |
| Hylidae | *Litoria freycineti* | 49.0 | 40.0 | 0.23 | R | 1.5 | - | 0 | 0 | 0 | 0 | 0 | 0 | 0 |
| Hylidae | *Litoria infrafrenata* | - | - | - | - | 3.0 | 300 | 0 | 0 | 0 | 0 | 0 | 0 | 0 |
| Hylidae | *Litoria kubori* | 60.0 | 45.0 | 0.33 | R | - | - | 0 | 0 | 0 | 0 | - | - | - |
| Hylidae | *Litoria meiriana* | - | - | - | - | - | 37 | 0 | 0 | 0 | 0 | 0 | 0 | 0 |
| Hylidae | *Litoria papua* | 60.0 | 50.0 | 0.20 | R | - | - | 0 | 0 | 0 | 0 | 0 | 0 | 0 |
| Hylidae | *Litoria peronii* | 55.7 | 48.4 | 0.15 | R | 1.5 | - | 1 | 0 | 0 | 1 | 0 | 0 | 0 |
| Hylidae | *Litoria rubella* | 34.9 | 32.9 | 0.06 | R | 1.1 | 170 | 0 | 0 | 0 | 0 | 0 | 0 | 0 |
| Hylidae | *Nyctimantis rugiceps* | 61.3 | 61.9 | -0.01 | M | - | - | 0 | 0 | 0 | 0 | 0 | 1 | 1 |
| Hylidae | *Osteocephalus alboguttatus* | 65.0 | 59.0 | 0.10 | R | 1.3 | - | 0 | 0 | 0 | 0 | 0 | 0 | 0 |
| Hylidae | *Osteocephalus buckleyi* | 61.7 | 43.3 | 0.42 | M | 1.4 | 1600 | 0 | 0 | 0 | 0 | 0 | 0 | 0 |
| Hylidae | *Osteocephalus leprieurii* | 57.1 | 44.7 | 0.28 | M | 1.0 | 848 | 0 | 0 | 0 | 0 | 0 | 0 | 0 |
| Hylidae | *Osteocephalus oophagus* | - | - | - | - | 1.7 | 516 | 0 | 0 | 0 | 0 | 0 | 1 | 1 |
| Hylidae | *Osteocephalus taurinus* | 81.0 | 69.9 | 0.16 | M | 1.0 | 550 | 0 | 0 | 0 | 0 | 0 | 0 | 0 |
| Hylidae | *Osteocephalus verruciger* | 64.5 | 53.0 | 0.22 | M | - | - | 0 | 0 | 0 | 0 | - | - | - |
| Hylidae | *Osteopilus brunneus* | 60.5 | 45.5 | 0.33 | M | 0.7 | 552 | 0 | 0 | 0 | 0 | 0 | 1 | 1 |
| Hylidae | *Osteopilus crucialis* | 104.0 | 92.6 | 0.12 | R | - | - | 0 | 0 | 0 | 0 | 0 | 1 | 1 |
| Hylidae | *Osteopilus dominicensis* | 76.8 | 57.8 | 0.33 | M | - | - | 0 | 0 | 0 | 0 | - | - | - |
| Hylidae | *Osteopilus marianae* | 38.7 | 28.0 | 0.38 | R | - | - | 0 | 0 | 0 | 0 | 0 | 0 | 0 |
| Hylidae | *Osteopilus pulchrilineatus* | 40.1 | 31.6 | 0.27 | M | - | - | 0 | 0 | 0 | 0 | 0 | 0 | 0 |
| Hylidae | *Osteopilus septentrionalis* | 71.2 | 53.9 | 0.32 | M | 3.0 | 130 | 1 | 0 | 1 | 0 | 0 | 0 | 0 |
| Hylidae | *Osteopilus vastus* | 136.9 | 96.7 | 0.42 | R | 2.5 | - | 0 | 0 | 0 | 0 | 0 | 0 | 0 |
| Hylidae | *Osteopilus wilderi* | 27.3 | 25.8 | 0.06 | M | - | - | - | - | - | - | 0 | 0 | 0 |
| Hylidae | *Pachymedusa dacnicolor* | 79.2 | 67.6 | 0.17 | M | 2.6 | 467 | 1 | 0 | 1 | 1 | 0 | 0 | 0 |
| Hylidae | *Phyllodytes luteolus* | - | - | - | - | 1.1 | 13 | - | - | - | - | 0 | 0 | 0 |
| Hylidae | *Phyllomedusa atelopoides* | 42.5 | 36.5 | 0.16 | R | 3.0 | 20 | 0 | 0 | 0 | 0 | 0 | 1 | 1 |
| Hylidae | *Phyllomedusa bicolor* | 115.0 | 97.0 | 0.19 | R | - | - | 0 | 0 | 0 | 0 | 0 | 0 | 0 |
| Hylidae | *Phyllomedusa hypochondrialis* | 44.0 | 35.2 | 0.25 | R | - | - | 1 | 0 | 0 | 1 | 0 | 0 | 0 |
| Hylidae | *Phyllomedusa palliata* | 46.3 | 42.1 | 0.10 | M | 2.5 | 60 | - | - | - | - | 0 | 0 | 0 |
| Hylidae | *Phyllomedusa tarsius* | 104.9 | 86.9 | 0.21 | M | 3.0 | 548 | 0 | 0 | 0 | 0 | 0 | 0 | 0 |
| Hylidae | *Phyllomedusa tomopterna* | 57.2 | 45.3 | 0.26 | M | 3.4 | 71 | - | - | - | - | 0 | 0 | 0 |
| Hylidae | *Phyllomedusa vaillantii* | 78.8 | 52.1 | 0.51 | M | 2.2 | 1114 | 0 | 0 | 0 | 0 | 0 | 0 | 0 |
| Hylidae | *Plectrohyla arborescandens* | 45.6 | 34.8 | 0.31 | M | 2.2 | - | 0 | 0 | 0 | 0 | 0 | 0 | 0 |
| Hylidae | *Plectrohyla glandulosa* | 44.3 | 44.6 | -0.01 | M | - | - | 0 | 0 | 0 | 0 | - | - | - |
| Hylidae | *Plectrohyla guatemalensis* | 48.6 | 47.5 | 0.02 | M | 2.0 | - | 0 | 0 | 0 | 0 | 0 | 0 | 0 |
| Hylidae | *Plectrohyla matudai* | 49.0 | 33.1 | 0.48 | R | - | - | 0 | 0 | 0 | 0 | 0 | 0 | 0 |
| Hylidae | *Plectrohyla pentheter* | 56.2 | 46.2 | 0.22 | R | - | - | 0 | 0 | 0 | 0 | - | - | - |
| Hylidae | *Pseudacris brachyphona* | 30.3 | 24.6 | 0.23 | M | 1.6 | 950 | 0 | 0 | 0 | 0 | 0 | 0 | 0 |
| Hylidae | *Pseudacris brimleyi* | 29.8 | 26.0 | 0.14 | R | - | 300 | 0 | 0 | 0 | 0 | 0 | 0 | 0 |
| Hylidae | *Pseudacris cadaverina* | 40.9 | 33.0 | 0.24 | M | 2.0 | - | 0 | 0 | 0 | 0 | 0 | 0 | 0 |
| Hylidae | *Pseudacris clarkii* | 28.0 | 24.5 | 0.14 | R | 0.8 | 1000 | 0 | 0 | 0 | 0 | 0 | 0 | 0 |
| Hylidae | *Pseudacris crucifer* | 31.8 | 25.2 | 0.26 | M | 1.1 | 750 | 1 | 0 | 0 | 1 | 0 | 0 | 0 |
| Hylidae | *Pseudacris feriarum* | 27.5 | 25.5 | 0.08 | R | 1.0 | - | 0 | 0 | 0 | 0 | 0 | 0 | 0 |
| Hylidae | *Pseudacris maculata* | 27.0 | 25.5 | 0.06 | R | 1.3 | - | 0 | 0 | 0 | 0 | 0 | 0 | 0 |
| Hylidae | *Pseudacris nigrita* | 26.0 | 24.5 | 0.06 | R | 1.0 | 59 | 0 | 0 | 0 | 0 | 0 | 0 | 0 |
| Hylidae | *Pseudacris ocularis* | 14.8 | 13.5 | 0.09 | R | 0.7 | 150 | 0 | 0 | 0 | 0 | 0 | 0 | 0 |
| Hylidae | *Pseudacris ornata* | 32.0 | 30.0 | 0.07 | R | 1.0 | 55 | 0 | 0 | 0 | 0 | 0 | 0 | 0 |
| Hylidae | *Pseudacris regilla* | 37.2 | 32.5 | 0.14 | M | 1.3 | 807 | 1 | 0 | 0 | 1 | 0 | 0 | 0 |
| Hylidae | *Pseudacris streckeri* | 39.0 | 33.0 | 0.18 | R | 1.4 | 450 | 0 | 0 | 0 | 0 | 0 | 0 | 0 |
| Hylidae | *Pseudacris triseriata* | 28.8 | 26.5 | 0.08 | R | 1.0 | 449 | 0 | 0 | 0 | 0 | 0 | 0 | 0 |
| Hylidae | *Ptychohyla euthysanota* | 38.2 | 35.0 | 0.09 | M | - | - | 0 | 0 | 0 | 0 | 0 | 0 | 0 |
| Hylidae | *Ptychohyla hypomykter* | - | - | - | - | 2.4 | - | - | - | - | - | 0 | 0 | 0 |
| Hylidae | *Ptychohyla leonhardschultzei* | 39.9 | 31.6 | 0.26 | M | - | - | 0 | 0 | 0 | 0 | - | - | - |
| Hylidae | *Ptychohyla spinipollex* | 42.8 | 37.1 | 0.15 | M | - | - | 0 | 0 | 0 | 0 | - | - | - |
| Hylidae | *Scarthyla goinorum* | 21.5 | 19.0 | 0.13 | R | - | - | - | - | - | - | 0 | 0 | 0 |
| Hylidae | *Scinax berthae* | 25.0 | 19.0 | 0.32 | M | 0.9 | - | 0 | 0 | 0 | 0 | 0 | 0 | 0 |
| Hylidae | *Scinax boulengeri* | 49.4 | 41.6 | 0.19 | M | 1.6 | 650 | 0 | 0 | 0 | 0 | 0 | 0 | 0 |
| Hylidae | *Scinax catharinae* | 42.7 | 33.0 | 0.29 | M | - | - | 0 | 0 | 0 | 0 | 0 | 0 | 0 |
| Hylidae | *Scinax elaeochrous* | 35.0 | 30.6 | 0.14 | M | - | - | 1 | 0 | 0 | 1 | 0 | 0 | 0 |
| Hylidae | *Scinax fuscovarius* | 47.6 | 44.6 | 0.07 | M | 1.0 | 2892 | 0 | 0 | 0 | 0 | 0 | 0 | 0 |
| Hylidae | *Scinax garbei* | 42.0 | 35.4 | 0.19 | M | 1.5 | 550 | 0 | 0 | 0 | 0 | 0 | 0 | 0 |
| Hylidae | *Scinax nasicus* | 31.0 | 31.5 | -0.02 | R | - | - | 0 | 0 | 0 | 0 | - | - | - |
| Hylidae | *Scinax ruber* | 39.1 | 32.6 | 0.20 | M | 1.5 | 591 | 0 | 0 | 0 | 0 | 0 | 0 | 0 |
| Hylidae | *Scinax squalirostris* | - | - | - | - | 1.0 | - | 0 | 0 | 0 | 0 | 0 | 0 | 0 |
| Hylidae | *Scinax staufferi* | 26.6 | 24.1 | 0.10 | M | - | - | 0 | 0 | 0 | 0 | - | - | - |
| Hylidae | *Smilisca baudinii* | 90.0 | 60.2 | 0.50 | R | 1.3 | 2225 | 0 | 0 | 0 | 0 | 0 | 0 | 0 |
| Hylidae | *Smilisca cyanosticta* | 70.0 | 56.0 | 0.25 | R | 1.2 | 1147 | 0 | 0 | 0 | 0 | 0 | 0 | 0 |
| Hylidae | *Smilisca fodiens* | 61.1 | 49.4 | 0.24 | M | - | - | 0 | 0 | 0 | 0 | - | - | - |
| Hylidae | *Smilisca phaeota* | 78.0 | 65.0 | 0.20 | R | - | 1829 | 0 | 0 | 0 | 0 | 0 | 0 | 0 |
| Hylidae | *Smilisca puma* | 46.0 | 38.0 | 0.21 | R | - | - | 0 | 0 | 0 | 0 | - | - | - |
| Hylidae | *Sphaenorhynchus lacteus* | 42.5 | 36.5 | 0.16 | R | 1.0 | - | 0 | 0 | 0 | 0 | 0 | 0 | 0 |
| Hylidae | *Tlalocohyla loquax* | 40.5 | 39.3 | 0.03 | M | - | 250 | 0 | 0 | 0 | 0 | 0 | 0 | 0 |
| Hylidae | *Tlalocohyla picta* | 22.1 | 20.2 | 0.09 | R | - | - | 0 | 0 | 0 | 0 | - | - | - |
| Hylidae | *Tlalocohyla smithii* | 27.7 | 24.3 | 0.14 | M | - | - | 0 | 0 | 0 | 0 | - | - | - |
| Hylidae | *Trachycephalus coriaceus* | 61.5 | 58.5 | 0.05 | R | 2.0 | 1430 | - | - | - | - | 0 | 0 | 0 |
| Hylidae | *Trachycephalus mesophaeus* | 80.5 | 68.5 | 0.18 | R | - | 700 | 1 | 0 | 1 | 0 | 0 | 0 | 0 |
| Hylidae | *Trachycephalus resinifictrix* | 76.0 | 76.0 | 0.00 | R | 1.6 | 436 | 1 | 0 | 0 | 1 | 0 | 1 | 1 |
| Hylidae | *Trachycephalus venulosus* | 98.1 | 88.6 | 0.11 | M | 2.8 | 5635 | 0 | 0 | 0 | 0 | 0 | 0 | 0 |
| Hylidae | *Triprion petasatus* | 70.7 | 54.6 | 0.29 | M | - | - | 0 | 0 | 0 | 0 | - | - | - |
| Hylidae | *Xenohyla truncata* | 42.0 | 38.0 | 0.11 | R | - | - | 0 | 0 | 0 | 0 | - | - | - |
| Hylodidae | *Hylodes phyllodes* | 27.6 | 27.2 | 0.01 | M | - | - | 1 | 0 | 0 | 1 | - | - | - |
| Hyperoliidae | *Afrixalus fornasini* | 35.0 | 33.5 | 0.04 | R | 1.6 | 80 | 0 | 0 | 0 | 0 | 0 | 0 | 0 |
| Hyperoliidae | *Alexteroon obstetricans* | - | - | - | - | - | - | 0 | 0 | 0 | 0 | 0 | 1 | 1 |
| Hyperoliidae | *Hyperolius lateralis* | 25.7 | 23.0 | 0.12 | M | 1.8 | 109 | - | - | - | - | 0 | 0 | 0 |
| Hyperoliidae | *Hyperolius marmoratus* | 29.6 | 28.9 | 0.02 | M | - | - | 1 | 0 | 0 | 1 | - | - | - |
| Hyperoliidae | *Hyperolius nasutus* | 22.2 | 22.3 | 0.00 | M | 1.0 | 200 | 1 | 0 | 0 | 1 | 0 | 0 | 0 |
| Hyperoliidae | *Hyperolius puncticulatus* | 33.5 | 25.0 | 0.34 | R | - | - | 0 | 0 | 0 | 0 | - | - | - |
| Hyperoliidae | *Hyperolius tuberilinguis* | - | - | - | - | 1.5 | 350 | 0 | 0 | 0 | 0 | 0 | 0 | 0 |
| Hyperoliidae | *Kassina senegalensis* | - | - | - | - | 1.6 | 400 | - | - | - | - | 0 | 0 | 0 |
| Leiopelmatidae | *Ascaphus truei* | - | - | - | - | 4.5 | 28 | 0 | 0 | 0 | 0 | 0 | 0 | 0 |
| Leiopelmatidae | *Leiopelma archeyi* | 37.0 | 31.0 | 0.19 | R | 4.5 | - | - | - | - | - | 1 | 0 | 1 |
| Leiopelmatidae | *Leiopelma hochstetteri* | 47.0 | 38.0 | 0.24 | R | 5.5 | - | 0 | 0 | 0 | 0 | 1 | 0 | 1 |
| Leiuperidae | *Edalorhina perezi* | 32.2 | 26.9 | 0.20 | M | 2.0 | 93 | - | - | - | - | - | - | 1 |
| Leiuperidae | *Pleurodema brachyops* | 34.5 | 31.5 | 0.10 | R | - | - | 0 | 0 | 0 | 0 | - | - | 1 |
| Leiuperidae | *Pleurodema bufoninum* | 56.0 | 45.0 | 0.24 | M | - | - | 0 | 0 | 0 | 0 | - | - | - |
| Leiuperidae | *Pleurodema thaul* | 49.7 | 41.7 | 0.19 | M | - | - | 0 | 0 | 0 | 0 | - | - | - |
| Leiuperidae | *Pseudopaludicola falcipes* | - | - | - | - | 1.0 | - | 0 | 0 | 0 | 0 | 0 | 0 | 0 |
| Leptodactylidae | *Leptodactylus albilabris* | 40.7 | 35.2 | 0.16 | M | 2.8 | 138 | 0 | 0 | 0 | 0 | 0 | 0 | 0 |
| Leptodactylidae | *Leptodactylus andreae* | 24.1 | 23.3 | 0.03 | M | 3.0 | 9 | - | - | - | - | 0 | 0 | 0 |
| Leptodactylidae | *Leptodactylus bufonius* | 60.0 | 56.4 | 0.06 | M | - | 550 | 0 | 0 | 0 | 0 | 1 | 0 | 1 |
| Leptodactylidae | *Leptodactylus fuscus* | 42.6 | 41.1 | 0.04 | M | 2.0 | - | 0 | 0 | 0 | 0 | 1 | 0 | 1 |
| Leptodactylidae | *Leptodactylus gracilis* | 43.0 | 43.0 | 0.00 | M | - | - | 0 | 0 | 0 | 0 | - | - | 1 |
| Leptodactylidae | *Leptodactylus hylaedactylus* | 23.2 | 22.7 | 0.02 | M | - | - | 0 | 0 | 0 | 0 | - | - | - |
| Leptodactylidae | *Leptodactylus labyrinthicus* | 127.3 | 136.5 | -0.07 | M | 2.3 | 2101 | 1 | 0 | 0 | 1 | 1 | 1 | 1 |
| Leptodactylidae | *Leptodactylus leptodactyloides* | 47.5 | 40.9 | 0.16 | M | - | - | 0 | 0 | 0 | 0 | - | - | - |
| Leptodactylidae | *Leptodactylus melanonotus* | 45.0 | 37.5 | 0.20 | R | 1.3 | 1500 | 1 | 0 | 0 | 1 | 1 | 1 | 1 |
| Leptodactylidae | *Leptodactylus mystaceus* | 46.9 | 45.1 | 0.04 | M | 2.5 | 281 | 0 | 0 | 0 | 0 | 0 | 0 | 0 |
| Leptodactylidae | *Leptodactylus mystacinus* | 53.0 | 56.5 | -0.06 | M | 2.5 | 124 | 0 | 0 | 0 | 0 | 1 | 0 | 1 |
| Leptodactylidae | *Leptodactylus ocellatus* | 60.0 | 71.5 | -0.16 | R | - | - | 0 | 0 | 0 | 0 | 1 | 1 | 1 |
| Leptodactylidae | *Leptodactylus pallidirostris* | 36.9 | 33.2 | 0.11 | M | - | - | 0 | 0 | 0 | 0 | - | - | - |
| Leptodactylidae | *Leptodactylus pentadactylus* | 151.5 | 141.5 | 0.07 | R | 2.5 | 1000 | 1 | 0 | 1 | 1 | 1 | 0 | 1 |
| Leptodactylidae | *Leptodactylus podicipinus* | 41.4 | 39.2 | 0.06 | M | - | - | 0 | 0 | 0 | 0 | - | - | - |
| Leptodactylidae | *Leptodactylus validus* | 44.3 | 37.8 | 0.17 | M | - | - | 0 | 0 | 0 | 0 | 0 | 1 | 1 |
| Leptodactylidae | *Leptodactylus wagneri* | 66.9 | 50.0 | 0.34 | M | 1.5 | 1740 | 0 | 0 | 0 | 0 | 0 | 0 | 0 |
| Leptodactylidae | *Lithodytes lineatus* | - | - | - | - | 2.0 | 195 | - | - | - | - | - | - | 1 |
| Leptodactylidae | *Vanzolinius discodactylus* | 33.6 | 27.4 | 0.23 | M | 1.0 | 234 | - | - | - | - | 0 | 0 | 0 |
| Limnodynastidae | *Adelotus brevis* | 32.8 | 35.5 | -0.08 | M | 2.0 | - | 1 | 0 | 0 | 1 | 1 | 0 | 1 |
| Limnodynastidae | *Heleioporus australiacus* | 77.0 | 80.0 | -0.04 | R | 2.5 | 400 | 0 | 0 | 0 | 0 | 0 | 0 | 0 |
| Limnodynastidae | *Lechriodus fletcheri* | - | - | - | - | 1.7 | 300 | 0 | 0 | 0 | 0 | - | - | 1 |
| Limnodynastidae | *Limnodynastes convexiusculus* | 51.0 | 44.0 | 0.16 | R | - | - | 0 | 0 | 0 | 0 | - | - | - |
| Limnodynastidae | *Limnodynastes dumerilii* | - | - | - | - | 1.7 | 3950 | 1 | 0 | 0 | 1 | 0 | 1 | 1 |
| Limnodynastidae | *Limnodynastes peronii* | 46.6 | 51.7 | -0.10 | M | 1.5 | 850 | 1 | 0 | 0 | 1 | - | - | 1 |
| Limnodynastidae | *Limnodynastes salmini* | - | - | - | - | - | 2000 | 0 | 0 | 0 | 0 | 0 | 0 | 0 |
| Limnodynastidae | *Limnodynastes tasmaniensis* | 34.9 | 33.2 | 0.05 | M | - | 250 | 0 | 0 | 0 | 0 | 0 | 1 | 1 |
| Limnodynastidae | *Limnodynastes terraereginae* | 76.0 | 69.0 | 0.10 | R | - | - | 0 | 0 | 0 | 0 | - | - | - |
| Limnodynastidae | *Neobatrachus pictus* | - | - | - | - | - | 1000 | 0 | 0 | 0 | 0 | 0 | 0 | 0 |
| Limnodynastidae | *Notaden melanoscaphus* | - | - | - | - | 1.4 | 833 | 0 | 0 | 0 | 0 | 0 | 0 | 0 |
| Limnodynastidae | *Philoria sphagnicolus* | - | - | - | - | - | 48 | 0 | 0 | 0 | 0 | 1 | 0 | 1 |
| Limnodynastidae | *Platyplectrum ornatum* | - | - | - | - | 1.2 | 645 | 0 | 0 | 0 | 0 | - | - | 1 |
| Mantellidae | *Aglyptodactylus madagascariensis* | - | - | - | - | 1.4 | 1600 | 0 | 0 | 0 | 0 | 0 | 0 | 0 |
| Mantellidae | *Boophis goudotii* | 80.0 | 50.0 | 0.60 | R | 2.0 | 1000 | 0 | 0 | 0 | 0 | 0 | 0 | 0 |
| Mantellidae | *Boophis luteus* | 54.0 | 38.5 | 0.40 | R | 2.0 | 120 | 0 | 0 | 0 | 0 | 0 | 0 | 0 |
| Mantellidae | *Boophis rappiodes* | 32.0 | 24.0 | 0.33 | R | 2.0 | 260 | 0 | 0 | 0 | 0 | 0 | 0 | 0 |
| Mantellidae | *Boophis tephraeomystax* | 45.5 | 38.5 | 0.18 | R | 1.4 | - | 0 | 0 | 0 | 0 | 0 | 0 | 0 |
| Mantellidae | *Gephyromantis asper* | 29.7 | 30.9 | -0.04 | M | - | - | 0 | 0 | 0 | 0 | - | - | - |
| Mantellidae | *Gephyromantis horridus* | 35.0 | 27.0 | 0.30 | R | - | - | 0 | 0 | 0 | 0 | - | - | - |
| Mantellidae | *Gephyromantis luteus* | 44.1 | 39.0 | 0.13 | M | - | - | 0 | 0 | 0 | 0 | - | - | - |
| Mantellidae | *Gephyromantis malagasius* | 24.5 | 22.0 | 0.11 | R | - | - | 0 | 0 | 0 | 0 | - | - | - |
| Mantellidae | *Gephyromantis plicifer* | 46.9 | 45.0 | 0.04 | M | - | - | 0 | 0 | 0 | 0 | - | - | - |
| Mantellidae | *Gephyromantis striatus* | 25.5 | 23.0 | 0.11 | R | - | - | 0 | 0 | 0 | 0 | - | - | - |
| Mantellidae | *Mantella aurantiaca* | - | - | - | - | 1.8 | 40 | 0 | 0 | 0 | 0 | 0 | 1 | 1 |
| Mantellidae | *Mantella laevigata* | 28.1 | 26.8 | 0.05 | M | - | 1 | 1 | 0 | 0 | 1 | 0 | 1 | 1 |
| Mantellidae | *Mantidactylus femoralis* | - | - | - | - | 2.5 | - | 0 | 0 | 0 | 0 | 0 | 0 | 0 |
| Megophryidae | *Leptobrachium ailaonicum* | 72.9 | 75.6 | -0.04 | M | 3.5 | 245 | 0 | 0 | 0 | 0 | 1 | 0 | 1 |
| Megophryidae | *Leptobrachium banae* | - | - | - | - | - | - | 0 | 0 | 0 | 0 | - | - | - |
| Megophryidae | *Leptobrachium boringii* | 66.8 | 76.7 | -0.13 | M | 3.0 | 298 | 0 | 0 | 0 | 0 | 1 | 0 | 1 |
| Megophryidae | *Leptobrachium chapaense* | 71.6 | 54.4 | 0.32 | M | - | - | 0 | 0 | 0 | 0 | - | - | - |
| Megophryidae | *Leptobrachium hasseltii* | 74.3 | 55.7 | 0.33 | M | - | - | 0 | 0 | 0 | 0 | - | - | - |
| Megophryidae | *Leptobrachium leishanense* | 69.9 | 78.7 | -0.11 | R | 3.9 | 295 | 0 | 0 | 0 | 0 | 1 | 0 | 1 |
| Megophryidae | *Leptobrachium liui* | 70.3 | 86.7 | -0.19 | M | 3.4 | 334 | 0 | 0 | 0 | 0 | 1 | 0 | 1 |
| Megophryidae | *Leptobrachium montanum* | 60.2 | 52.1 | 0.16 | M | - | - | - | - | - | - | - | - | - |
| Megophryidae | *Leptobrachium mouhoti* | 70.2 | 58.3 | 0.20 | R | - | - | 0 | 0 | 0 | 0 | - | - | - |
| Megophryidae | *Leptobrachium promustache* | 61.1 | 56.7 | 0.08 | M | - | - | 0 | 0 | 0 | 0 | - | - | - |
| Megophryidae | *Leptolalax liui* | 25.6 | 26.3 | -0.03 | R | - | - | 0 | 0 | 0 | 0 | - | - | - |
| Megophryidae | *Leptolalax pelodytoides* | 29.2 | 26.0 | 0.13 | M | 2.0 | 144 | 0 | 0 | 0 | 0 | - | - | - |
| Megophryidae | *Oreolalax jingdongensis* | 52.6 | 54.3 | -0.03 | M | 3.6 | 170 | 0 | 0 | 0 | 0 | 0 | 0 | 0 |
| Megophryidae | *Oreolalax liangbeiensis* | 60.0 | 51.6 | 0.16 | M | 3.5 | 350 | 0 | 0 | 0 | 0 | 0 | 0 | 0 |
| Megophryidae | *Oreolalax multipunctatus* | - | - | - | - | 3.5 | 78 | 0 | 0 | 0 | 0 | 1 | 0 | 1 |
| Megophryidae | *Oreolalax pingii* | 45.6 | 45.2 | 0.01 | M | 3.4 | 175 | 0 | 0 | 0 | 0 | 0 | 0 | 0 |
| Megophryidae | *Oreolalax popei* | 61.9 | 65.2 | -0.05 | M | 3.3 | 350 | 0 | 0 | 0 | 0 | - | - | - |
| Megophryidae | *Oreolalax rugosus* | 49.7 | 47.1 | 0.06 | M | - | - | 0 | 0 | 0 | 0 | - | - | - |
| Megophryidae | *Oreolalax schmidti* | 51.0 | 43.1 | 0.18 | M | 4.0 | 120 | 1 | 0 | 1 | 0 | 0 | 0 | 0 |
| Megophryidae | *Scutiger boulengeri* | 53.8 | 47.4 | 0.13 | M | 3.0 | 380 | 0 | 0 | 0 | 0 | - | - | - |
| Megophryidae | *Scutiger glandulatus* | 66.5 | 67.0 | -0.01 | M | 3.5 | - | 0 | 0 | 0 | 0 | 0 | 0 | 0 |
| Megophryidae | *Scutiger mammatus* | 69.2 | 70.5 | -0.02 | M | 2.9 | 606 | 0 | 0 | 0 | 0 | 1 | 0 | 1 |
| Megophryidae | *Scutiger muliensis* | 63.8 | 73.4 | -0.13 | M | - | 200 | 0 | 0 | 0 | 0 | - | - | - |
| Megophryidae | *Scutiger tuberculatus* | 71.5 | 72.0 | -0.01 | M | 3.0 | - | 0 | 0 | 0 | 0 | 0 | 0 | 0 |
| Megophyidae | *Brachytarsophrys feae* | 101.2 | 95.6 | 0.06 | M | 4.5 | 1000 | 0 | 0 | 0 | 0 | 0 | 0 | 0 |
| Megophyidae | *Ophryophryne microstoma* | 47.3 | 30.6 | 0.55 | M | 1.8 | - | - | - | - | - | 0 | 0 | 0 |
| Megophyidae | *Oreolalax chuanbeiensis* | 56.7 | 53.0 | 0.07 | M | 3.5 | 170 | 0 | 0 | 0 | 0 | 1 | 0 | 1 |
| Megophyidae | *Oreolalax lichuanensis* | 59.1 | 58.7 | 0.01 | M | 3.7 | 215 | 0 | 0 | 0 | 0 | 0 | 0 | 0 |
| Megophyidae | *Oreolalax major* | 67.5 | 64.6 | 0.04 | M | 3.8 | - | 0 | 0 | 0 | 0 | 0 | 0 | 0 |
| Megophyidae | *Oreolalax omeimontis* | 54.1 | 53.7 | 0.01 | M | 3.2 | 183 | - | - | - | - | - | - | - |
| Megophyidae | *Oreolalax xiangchengensis* | 57.4 | 48.6 | 0.18 | M | 3.8 | - | - | - | - | - | - | - | - |
| Megophyidae | *Scutiger chintingensis* | 50.6 | 42.2 | 0.20 | M | 3.5 | 150 | 0 | 0 | 0 | 0 | 0 | 0 | 0 |
| Megophyidae | *Xenophrys minor* | 43.4 | 36.8 | 0.18 | M | - | - | 0 | 0 | 0 | 0 | - | - | - |
| Megophyidae | *Xenophrys omeimontis* | 64.1 | 55.2 | 0.16 | M | 2.8 | 357 | 0 | 0 | 0 | 0 | 0 | 0 | 0 |
| Megophyidae | *Xenophrys shapingensis* | 94.0 | 68.0 | 0.38 | R | 3.2 | - | 0 | 0 | 0 | 0 | - | - | - |
| Megophyidae | *Xenophrys spinata* | 54.5 | 50.0 | 0.09 | R | 3.0 | - | 0 | 0 | 0 | 0 | - | - | - |
| Microhylidae | *Chaperina fusca* | 22.4 | 19.2 | 0.17 | M | - | - | 0 | 0 | 0 | 0 | - | - | - |
| Microhylidae | *Gastrophryne olivacea* | - | - | - | - | 0.9 | 622 | 0 | 0 | 0 | 0 | 0 | 0 | 0 |
| Microhylidae | *Hylophorbus rufescens* | 40.5 | 30.0 | 0.35 | R | 5.4 | 13 | 0 | 0 | 0 | 0 | 1 | 1 | 1 |
| Microhylidae | *Kalophrynus pleurostigma* | 44.8 | 40.4 | 0.11 | M | 1.0 | 4000 | - | - | - | - | 0 | 0 | 0 |
| Microhylidae | *Kaloula pulchra* | 63.6 | 60.9 | 0.04 | M | 1.3 | 4126 | 0 | 0 | 0 | 0 | 0 | 0 | 0 |
| Microhylidae | *Metaphrynella sundana* | 23.4 | 20.9 | 0.12 | R | - | - | 0 | 0 | 0 | 0 | - | - | - |
| Microhylidae | *Microhyla butleri* | 23.0 | 23.2 | -0.01 | M | 1.0 | - | 0 | 0 | 0 | 0 | - | - | - |
| Microhylidae | *Microhyla heymonsi* | 22.2 | 19.8 | 0.12 | M | 0.7 | 157 | 0 | 0 | 0 | 0 | 0 | 0 | 0 |
| Microhylidae | *Microhyla ornata* | 23.9 | 23.2 | 0.03 | M | 0.8 | 225 | 0 | 0 | 0 | 0 | 0 | 0 | 0 |
| Microhylidae | *Microhyla pulchra* | 33.1 | 30.0 | 0.10 | M | 1.1 | 1256 | 0 | 0 | 0 | 0 | 0 | 0 | 0 |
| Microhylidae | *Phrynomantis bifasciatus* | - | - | - | - | 1.3 | 600 | 0 | 0 | 0 | 0 | 1 | 0 | 1 |
| Microphylidae | *Aphantophryne pansa* | - | - | - | - | - | 17 | 0 | 0 | 0 | 0 | 0 | 0 | 0 |
| Myobatrachidae | *Crinia nimbus* | - | - | - | - | 3.5 | 10 | 0 | 0 | 0 | 0 | 1 | 0 | 1 |
| Myobatrachidae | *Crinia signifera* | 21.7 | 18.8 | 0.16 | M | 1.5 | 150 | 0 | 0 | 0 | 0 | 0 | 0 | 0 |
| Myobatrachidae | *Geocrinia victoriana* | - | - | - | - | 2.2 | 126 | 0 | 0 | 0 | 0 | 0 | 0 | 0 |
| Myobatrachidae | *Metacrinia nichollsi* | 25.0 | 23.0 | 0.09 | R | - | 27 | 0 | 0 | 0 | 0 | 0 | 0 | 0 |
| Myobatrachidae | *Mixophyes fasciolatus* | 97.0 | 63.0 | 0.54 | R | 1.6 | 970 | 0 | 0 | 0 | 0 | 0 | 0 | 0 |
| Myobatrachidae | *Myobatrachus gouldii* | 57.0 | 44.0 | 0.30 | R | 5.1 | 40 | 0 | 0 | 0 | 0 | 0 | 0 | 0 |
| Myobatrachidae | *Paracrinia haswelli* | 32.0 | 26.0 | 0.23 | R | - | - | 0 | 0 | 0 | 0 | 0 | 0 | 0 |
| Myobatrachidae | *Pseudophryne bibronii* | 27.0 | 26.0 | 0.04 | R | 2.1 | 82 | 1 | 0 | 0 | 1 | 1 | 0 | 1 |
| Myobatrachidae | *Rheobatrachus silus* | - | - | - | - | 4.7 | 25 | 0 | 0 | 0 | 0 | 0 | 1 | 1 |
| Myobatrachidae | *Spicospina flammocaerulea* | - | - | - | - | - | 200 | 0 | 0 | 0 | 0 | 0 | 0 | 0 |
| Myobatrachidae | *Uperoleia laevigata* | - | - | - | - | - | - | 0 | 0 | 0 | 0 | 0 | 0 | 0 |
| Nyctibatrachidae | *Lankanectes corrugatus* | - | - | - | - | - | - | 0 | 0 | 0 | 0 | 0 | 0 | 0 |
| Pelobatidae | *Pelobates fuscus* | 42.6 | 41.1 | 0.04 | R | 1.7 | 1740 | 1 | 0 | 1 | 0 | 0 | 0 | 0 |
| Pelobatidae | *Pelobates syriacus* | - | - | - | - | 1.4 | 6000 | 0 | 0 | 0 | 0 | - | - | - |
| Pelodytidae | *Pelodytes caucasicus* | - | - | - | - | - | - | 1 | 0 | 1 | 0 | - | - | - |
| Petropedetidae | *Conraua goliath* | 254.0 | 270.0 | -0.06 | M | 3.5 | 100 | - | - | - | - | 0 | 0 | 0 |
| Petropedetidae | *Petropedetes yakusini* | 56.0 | 73.0 | -0.23 | R | 2.0 | 200 | 0 | 0 | 0 | 0 | 1 | 0 | 1 |
| Phrynobatrachidae | *Phrynobatrachus mababiensis* | - | - | - | - | 0.9 | - | 0 | 0 | 0 | 0 | 0 | 0 | 0 |
| Phrynobatrachidae | *Phrynobatrachus natalensis* | 28.5 | 27.5 | 0.04 | R | 1.0 | 650 | 0 | 0 | 0 | 0 | 0 | 0 | 0 |
| Pipidae | *Hymenochirus boettgeri* | - | - | - | - | 0.9 | 649 | 1 | 0 | 0 | 1 | 0 | 0 | 0 |
| Pipidae | *Pipa carvalhoi* | 51.4 | 45.7 | 0.12 | M | 2.1 | 140 | 1 | 0 | 0 | 1 | 0 | 1 | 1 |
| Pipidae | *Pipa parva* | 33.6 | 31.7 | 0.06 | M | - | - | 1 | 0 | 0 | 1 | 0 | 1 | 1 |
| Pipidae | *Pipa pipa* | 139.4 | 128.7 | 0.08 | M | 6.0 | 65 | 1 | 0 | 0 | 1 | 0 | 1 | 1 |
| Pipidae | *Xenopus laevis* | - | - | - | - | 1.4 | 17000 | 1 | 0 | 0 | 1 | 0 | 0 | 0 |
| Pipidae | *Xenopus muelleri* | 72.0 | 58.0 | 0.24 | R | - | - | 0 | 0 | 0 | 0 | - | - | - |
| Ptychadenidae | *Ptychadena anchietae* | - | - | - | - | - | - | 0 | 0 | 0 | 0 | - | - | - |
| Ptychadenidae | *Ptychadena mascareniensis* | - | - | - | - | 1.1 | - | 0 | 0 | 0 | 0 | 0 | 0 | 0 |
| Pyxicephalidae | *Afrana angolensis* | - | - | - | - | 1.5 | - | 0 | 0 | 0 | 0 | 0 | 0 | 0 |
| Pyxicephalidae | *Afrana fuscigula* | - | - | - | - | 1.5 | 15000 | 0 | 0 | 0 | 0 | 0 | 0 | 0 |
| Pyxicephalidae | *Anhydrophryne rattrayi* | - | - | - | - | - | 15 | 0 | 0 | 0 | 0 | 0 | 0 | 0 |
| Pyxicephalidae | *Natalobatrachus bonebergi* | - | - | - | - | 2.6 | 87 | 0 | 0 | 0 | 0 | 0 | 0 | 0 |
| Pyxicephalidae | *Pyxicephalus adspersus* | 11.5 | 20.3 | -0.43 | M | 2.0 | 4000 | 0 | 0 | 0 | 0 | 1 | 0 | 1 |
| Pyxicephalidae | *Strongylopus grayii* | - | - | - | - | - | - | 0 | 0 | 0 | 0 | 0 | 0 | 0 |
| Pyxicephalidae | *Tomopterna delalandii* | - | - | - | - | 1.5 | - | 0 | 0 | 0 | 0 | 0 | 0 | 0 |
| Ranidae | *Amolops bellulus* | 63.6 | 47.9 | 0.33 | R | - | - | 0 | 0 | 0 | 0 | - | - | - |
| Ranidae | *Amolops chunganensis* | 48.1 | 39.6 | 0.21 | M | 3.0 | 436 | 1 | 0 | 1 | 0 | 0 | 0 | 0 |
| Ranidae | *Amolops cremnobatus* | - | - | - | - | 3.6 | - | 0 | 0 | 0 | 0 | 0 | 0 | 0 |
| Ranidae | *Amolops daiyunensis* | 54.5 | 49.4 | 0.10 | M | 2.5 | - | - | - | - | - | 0 | 0 | 0 |
| Ranidae | *Amolops granulosus* | 51.9 | 39.8 | 0.30 | R | - | - | 0 | 0 | 0 | 0 | 0 | 0 | 0 |
| Ranidae | *Amolops hainanensis* | 72.9 | 80.4 | -0.09 | M | 2.7 | - | - | - | - | - | 0 | 0 | 0 |
| Ranidae | *Amolops hongkongensis* | 40.0 | 39.0 | 0.03 | M | 2.0 | - | - | - | - | - | - | - | - |
| Ranidae | *Amolops lifanensis* | 71.1 | 54.2 | 0.31 | M | 4.0 | - | - | - | - | - | 0 | 0 | 0 |
| Ranidae | *Amolops loloensis* | 74.2 | 58.3 | 0.27 | M | 4.0 | 153 | - | - | - | - | 0 | 0 | 0 |
| Ranidae | *Amolops mantzorum* | 65.8 | 52.2 | 0.26 | M | 2.5 | - | 0 | 0 | 0 | 0 | 0 | 0 | 0 |
| Ranidae | *Amolops ricketti* | 60.7 | 55.9 | 0.09 | M | 3.0 | 990 | 0 | 0 | 0 | 0 | 0 | 0 | 0 |
| Ranidae | *Amolops torrentis* | 37.4 | 29.7 | 0.26 | M | 2.8 | - | 0 | 0 | 0 | 0 | - | - | - |
| Ranidae | *Amolops viridimaculatus* | 98.0 | 74.8 | 0.31 | M | - | - | 0 | 0 | 0 | 0 | 0 | 0 | 0 |
| Ranidae | *Amolops wuyiensis* | 48.2 | 41.8 | 0.15 | M | 2.0 | 600 | 0 | 0 | 0 | 0 | 0 | 0 | 0 |
| Ranidae | *Babina holsti* | 103.0 | 118.7 | -0.13 | R | 2.7 | - | - | - | - | - | 0 | 0 | 0 |
| Ranidae | *Babina okinavana* | 43.5 | 40.2 | 0.08 | M | - | 500 | 0 | 0 | 0 | 0 | 0 | 0 | 0 |
| Ranidae | *Babina pleuraden* | 49.8 | 51.0 | -0.02 | M | 2.0 | 606 | 0 | 0 | 0 | 0 | 0 | 0 | 0 |
| Ranidae | *Fejervarya nicobariensis* | 50.1 | 42.8 | 0.17 | M | - | - | 0 | 0 | 0 | 0 | 0 | 0 | 0 |
| Ranidae | *Glandirana emeljanovi* | 55.4 | 46.0 | 0.20 | M | 1.8 | 1130 | 0 | 0 | 0 | 0 | 0 | 0 | 0 |
| Ranidae | *Glandirana minima* | 28.6 | 27.0 | 0.06 | M | 1.3 | 274 | 0 | 0 | 0 | 0 | 0 | 0 | 0 |
| Ranidae | *Glandirana rugosa* | 44.7 | 42.8 | 0.04 | M | 1.6 | 1098 | - | - | - | - | 0 | 0 | 0 |
| Ranidae | *Glandirana tientaiensis* | 31.3 | 30.7 | 0.02 | M | 1.5 | 1350 | 0 | 0 | 0 | 0 | 0 | 0 | 0 |
| Ranidae | *Hydrophylax galamensis* | - | - | - | - | 1.7 | 5135 | 0 | 0 | 0 | 0 | 0 | 0 | 0 |
| Ranidae | *Hylarana baramica* | 57.3 | 42.8 | 0.34 | M | - | - | 0 | 0 | 0 | 0 | - | - | - |
| Ranidae | *Hylarana chalconota* | - | - | - | - | - | - | 0 | 0 | 0 | 0 | - | - | - |
| Ranidae | *Hylarana daemeli* | - | - | - | - | - | - | 1 | 0 | 1 | 0 | 0 | 0 | 0 |
| Ranidae | *Hylarana erythraea* | 65.7 | 39.7 | 0.66 | M | 2.0 | - | 0 | 0 | 0 | 0 | 0 | 0 | 0 |
| Ranidae | *Hylarana guentheri* | 74.9 | 69.8 | 0.07 | M | 1.5 | 3000 | 0 | 0 | 0 | 0 | 0 | 0 | 0 |
| Ranidae | *Hylarana latouchii* | 47.0 | 37.5 | 0.25 | M | 1.5 | 1126 | 0 | 0 | 0 | 0 | 0 | 0 | 0 |
| Ranidae | *Hylarana macrodactyla* | 39.8 | 28.0 | 0.42 | M | 1.3 | 300 | - | - | - | - | - | - | - |
| Ranidae | *Hylarana nigrovittata* | 47.2 | 42.7 | 0.11 | M | 1.4 | 330 | 0 | 0 | 0 | 0 | 0 | 0 | 0 |
| Ranidae | *Hylarana signata* | 56.2 | 39.4 | 0.42 | M | 1.7 | - | 0 | 0 | 0 | 0 | 0 | 0 | 0 |
| Ranidae | *Hylarana taipehensis* | 39.0 | 29.0 | 0.34 | M | 1.2 | 189 | 0 | 0 | 0 | 0 | 0 | 0 | 0 |
| Ranidae | *Hylarana temporalis* | - | - | - | - | 2.5 | - | 0 | 0 | 0 | 0 | 0 | 0 | 0 |
| Ranidae | *Lithobates areolatus* | - | - | - | - | 2.5 | 7000 | 1 | 0 | 1 | 0 | 0 | 0 | 0 |
| Ranidae | *Lithobates berlandieri* | - | - | - | - | - | - | 0 | 0 | 0 | 0 | 0 | 0 | 0 |
| Ranidae | *Lithobates blairi* | - | - | - | - | - | 5250 | 0 | 0 | 0 | 0 | 0 | 0 | 0 |
| Ranidae | *Lithobates capito* | - | - | - | - | 2.0 | 7000 | 0 | 0 | 0 | 0 | 0 | 0 | 0 |
| Ranidae | *Lithobates catesbeianus* | 119.3 | 119.3 | 0.00 | M | 1.7 | 18171 | 1 | 0 | 0 | 1 | 0 | 0 | 0 |
| Ranidae | *Lithobates chiricahuensis* | - | - | - | - | - | 892 | 0 | 0 | 0 | 0 | 0 | 0 | 0 |
| Ranidae | *Lithobates clamitans* | - | - | - | - | 1.5 | 5500 | 1 | 0 | 0 | 1 | 0 | 0 | 0 |
| Ranidae | *Lithobates forreri* | - | - | - | - | - | - | 0 | 0 | 0 | 0 | - | - | - |
| Ranidae | *Lithobates grylio* | - | - | - | - | - | 7504 | 0 | 0 | 0 | 0 | 0 | 0 | 0 |
| Ranidae | *Lithobates heckscheri* | - | - | - | - | 1.8 | 7000 | 0 | 0 | 0 | 0 | 0 | 0 | 0 |
| Ranidae | *Lithobates onca* | - | - | - | - | - | 200 | 0 | 0 | 0 | 0 | 0 | 0 | 0 |
| Ranidae | *Lithobates palmipes* | 116.8 | 92.3 | 0.27 | M | 2.0 | 2860 | 0 | 0 | 0 | 0 | 0 | 0 | 0 |
| Ranidae | *Lithobates palustris* | 66.4 | 53.0 | 0.25 | M | 1.6 | 2500 | 1 | 0 | 1 | 0 | 0 | 0 | 0 |
| Ranidae | *Lithobates pipiens* | 78.6 | 69.8 | 0.13 | M | 2.0 | 3045 | 1 | 0 | 1 | 0 | 0 | 0 | 0 |
| Ranidae | *Lithobates septentrionalis* | - | - | - | - | 1.7 | 500 | 0 | 0 | 0 | 0 | 0 | 0 | 0 |
| Ranidae | *Lithobates sevosus* | 85.4 | 80.6 | 0.06 | M | 1.8 | 6600 | 0 | 0 | 0 | 0 | 0 | 0 | 0 |
| Ranidae | *Lithobates sphenocephalus* | 63.7 | 55.8 | 0.14 | M | 1.6 | 1500 | 0 | 0 | 0 | 0 | 0 | 0 | 0 |
| Ranidae | *Lithobates sylvaticus* | 61.1 | 53.6 | 0.14 | M | 2.4 | 781 | 1 | 0 | 1 | 0 | 0 | 0 | 0 |
| Ranidae | *Lithobates tarahumarae* | - | - | - | - | 2.2 | 2200 | 0 | 0 | 0 | 0 | 0 | 0 | 0 |
| Ranidae | *Lithobates vaillanti* | - | - | - | - | - | - | 0 | 0 | 0 | 0 | - | - | - |
| Ranidae | *Lithobates vibicarius* | - | - | - | - | 2.5 | - | 0 | 0 | 0 | 0 | 0 | 0 | 0 |
| Ranidae | *Lithobates virgatipes* | - | - | - | - | 1.8 | 400 | 1 | 0 | 0 | 1 | 0 | 0 | 0 |
| Ranidae | *Lithobates warszewitschii* | - | - | - | - | - | - | 0 | 0 | 0 | 0 | - | - | - |
| Ranidae | *Meristogenys jerboa* | 67.1 | 36.4 | 0.84 | M | 1.3 | 2462 | 0 | 0 | 0 | 0 | 0 | 0 | 0 |
| Ranidae | *Meristogenys kinabaluensis* | 88.0 | 63.6 | 0.38 | M | 2.5 | - | 0 | 0 | 0 | 0 | - | - | - |
| Ranidae | *Odorrana andersonii* | 77.3 | 63.5 | 0.22 | M | 2.6 | - | 0 | 0 | 0 | 0 | - | - | - |
| Ranidae | *Odorrana banaorum* | 91.0 | 48.5 | 0.88 | R | 2.0 | - | - | - | - | - | 0 | 0 | 0 |
| Ranidae | *Odorrana chapaensis* | 92.2 | 81.1 | 0.14 | M | - | - | - | - | - | - | - | - | - |
| Ranidae | *Odorrana chloronota* | 92.0 | 46.0 | 1.00 | M | 2.0 | - | - | - | - | - | 0 | 0 | 0 |
| Ranidae | *Odorrana grahami* | 93.3 | 74.3 | 0.26 | M | 2.5 | 2446 | 0 | 0 | 0 | 0 | 0 | 0 | 0 |
| Ranidae | *Odorrana hosii* | 92.4 | 53.8 | 0.72 | M | 1.9 | 2320 | - | - | - | - | 0 | 0 | 0 |
| Ranidae | *Odorrana jingdongensis* | 97.0 | 74.8 | 0.30 | M | 2.5 | - | - | - | - | - | - | - | - |
| Ranidae | *Odorrana junlianensis* | 97.5 | 76.1 | 0.28 | M | 2.5 | - | - | - | - | - | - | - | - |
| Ranidae | *Odorrana livida* | 100.4 | 56.5 | 0.78 | M | 1.9 | 1433 | 0 | 0 | 0 | 0 | 0 | 0 | 0 |
| Ranidae | *Odorrana margaretae* | 103.4 | 81.4 | 0.27 | M | - | 705 | 0 | 0 | 0 | 0 | - | - | - |
| Ranidae | *Odorrana morafkai* | 90.0 | 42.5 | 1.12 | R | 2.0 | - | - | - | - | - | 0 | 0 | 0 |
| Ranidae | *Odorrana schmackeri* | 76.8 | 40.8 | 0.88 | M | 2.0 | 1549 | 0 | 0 | 0 | 0 | 0 | 0 | 0 |
| Ranidae | *Odorrana swinhoana* | 73.5 | 60.3 | 0.22 | M | 3.0 | 45 | 0 | 0 | 0 | 0 | 0 | 0 | 0 |
| Ranidae | *Odorrana tiannanensis* | 100.0 | 52.0 | 0.92 | M | 2.0 | - | - | - | - | - | 0 | 0 | 0 |
| Ranidae | *Odorrana tormota* | 59.5 | 33.8 | 0.76 | M | 2.5 | 538 | 0 | 0 | 0 | 0 | 0 | 0 | 0 |
| Ranidae | *Odorrana versabilis* | 62.3 | 46.4 | 0.34 | M | 3.0 | 101 | 0 | 0 | 0 | 0 | 0 | 0 | 0 |
| Ranidae | *Pelophylax esculenta* | 64.9 | 46.5 | 0.40 | M | 1.4 | 1048 | 0 | 0 | 0 | 0 | 0 | 0 | 0 |
| Ranidae | *Pelophylax fukienensis* | 55.5 | 40.2 | 0.38 | M | 1.6 | 1011 | 0 | 0 | 0 | 0 | 0 | 0 | 0 |
| Ranidae | *Pelophylax hubeiensis* | - | - | - | - | 1.8 | 1829 | 1 | 0 | 1 | 0 | 0 | 0 | 0 |
| Ranidae | *Pelophylax lessonae* | - | - | - | - | - | 2500 | 1 | 0 | 1 | 0 | 0 | 0 | 0 |
| Ranidae | *Pelophylax nigromaculatus* | 70.4 | 64.4 | 0.09 | M | 2.2 | 3250 | 0 | 0 | 0 | 0 | 0 | 0 | 0 |
| Ranidae | *Pelophylax plancyi* | 56.4 | 39.2 | 0.44 | M | 1.3 | 945 | 1 | 0 | 1 | 0 | 0 | 0 | 0 |
| Ranidae | *Pelophylax ridibundus* | - | - | - | - | 1.9 | 5884 | 1 | 0 | 1 | 0 | 0 | 0 | 0 |
| Ranidae | *Pseudorana weiningensis* | 43.0 | 35.0 | 0.23 | M | 2.3 | 313 | 1 | 0 | 1 | 0 | 0 | 0 | 0 |
| Ranidae | *Rana adenopleura* | 52.2 | 50.2 | 0.04 | M | 2.2 | 352 | 0 | 0 | 0 | 0 | 1 | 0 | 1 |
| Ranidae | *Rana amurensis* | 52.8 | 41.0 | 0.29 | M | 2.3 | 2045 | 0 | 0 | 0 | 0 | 0 | 0 | 0 |
| Ranidae | *Rana arvalis* | - | - | - | - | 2.0 | 1599 | 1 | 0 | 1 | 0 | 0 | 0 | 0 |
| Ranidae | *Rana asiatica* | 53.8 | 53.4 | 0.01 | M | 2.3 | 1150 | 0 | 0 | 0 | 0 | 0 | 0 | 0 |
| Ranidae | *Rana aurora* | - | - | - | - | 3.6 | 800 | 1 | 0 | 1 | 0 | 0 | 0 | 0 |
| Ranidae | *Rana boylii* | 73.0 | 56.0 | 0.30 | M | 2.5 | 980 | 0 | 0 | 0 | 0 | 0 | 0 | 0 |
| Ranidae | *Rana cascadae* | - | - | - | - | 2.8 | 400 | 0 | 0 | 0 | 0 | 0 | 0 | 0 |
| Ranidae | *Rana chaochiaoensis* | 52.9 | 53.6 | -0.01 | M | 1.8 | 1500 | 0 | 0 | 0 | 0 | 0 | 0 | 0 |
| Ranidae | *Rana chensinensis* | 41.9 | 48.0 | -0.13 | M | - | 1400 | 0 | 0 | 0 | 0 | 0 | 0 | 0 |
| Ranidae | *Rana dalmatina* | 56.7 | 45.2 | 0.25 | M | 3.0 | 1068 | 1 | 0 | 0 | 1 | 0 | 0 | 0 |
| Ranidae | *Rana dybowskii* | - | - | - | - | 3.0 | 1550 | 0 | 0 | 0 | 0 | 0 | 0 | 0 |
| Ranidae | *Rana graeca* | - | - | - | - | 2.8 | 1100 | 0 | 0 | 0 | 0 | 1 | 0 | 1 |
| Ranidae | *Rana huanrenensis* | 45.3 | 42.8 | 0.06 | M | 2.0 | 450 | 0 | 0 | 0 | 0 | 0 | 0 | 0 |
| Ranidae | *Rana japonica* | 53.7 | 48.1 | 0.12 | M | 2.1 | 1500 | 1 | 0 | 1 | 0 | 0 | 0 | 0 |
| Ranidae | *Rana johnsi* | 46.5 | 43.1 | 0.08 | M | 2.3 | 1500 | 0 | 0 | 0 | 0 | 0 | 0 | 0 |
| Ranidae | *Rana kukunoris* | 61.9 | 56.3 | 0.10 | M | 1.8 | 1350 | 0 | 0 | 0 | 0 | 0 | 0 | 0 |
| Ranidae | *Rana kunyuensis* | 44.8 | 40.9 | 0.10 | M | 1.8 | 918 | 0 | 0 | 0 | 0 | 0 | 0 | 0 |
| Ranidae | *Rana longicrus* | 47.9 | 41.5 | 0.15 | M | - | 400 | 0 | 0 | 0 | 0 | 0 | 0 | 0 |
| Ranidae | *Rana luteiventris* | - | - | - | - | 2.1 | 2400 | 0 | 0 | 0 | 0 | 0 | 0 | 0 |
| Ranidae | *Rana macrocnemis* | - | - | - | - | - | 2040 | 0 | 0 | 0 | 0 | 0 | 0 | 0 |
| Ranidae | *Rana muscosa* | - | - | - | - | 2.3 | 223 | 0 | 0 | 0 | 0 | 0 | 0 | 0 |
| Ranidae | *Rana omeimontis* | 66.7 | 60.1 | 0.11 | M | 2.0 | 1550 | 0 | 0 | 0 | 0 | 0 | 0 | 0 |
| Ranidae | *Rana ornativentris* | 58.5 | 49.3 | 0.19 | M | 1.7 | 12000 | 0 | 0 | 0 | 0 | 0 | 0 | 0 |
| Ranidae | *Rana pretiosa* | 69.8 | 56.0 | 0.25 | R | 2.2 | 997 | 1 | 0 | 1 | 0 | 0 | 0 | 0 |
| Ranidae | *Rana sauteri* | 50.6 | 39.9 | 0.27 | M | 2.6 | 523 | 0 | 0 | 0 | 0 | 0 | 0 | 0 |
| Ranidae | *Rana shuchinae* | 44.5 | 39.9 | 0.12 | M | 2.2 | 180 | 0 | 0 | 0 | 0 | 0 | 0 | 0 |
| Ranidae | *Rana tagoi* | 41.3 | 39.0 | 0.06 | R | 3.5 | - | - | - | - | - | 0 | 0 | 0 |
| Ranidae | *Rana temporaria* | 61.2 | 64.2 | -0.05 | M | 2.4 | 2247 | 1 | 0 | 1 | 0 | 0 | 0 | 0 |
| Ranidae | *Rana tsushimensis* | 49.8 | 41.1 | 0.21 | M | 2.0 | 450 | 0 | 0 | 0 | 0 | 0 | 0 | 0 |
| Ranidae | *Rana zhenhaiensis* | 48.1 | 46.4 | 0.04 | M | 1.8 | 883 | 1 | 0 | 1 | 0 | 0 | 0 | 0 |
| Ranidae | *Staurois latopalmatus* | 61.4 | 45.3 | 0.36 | M | - | - | - | - | - | - | - | - | - |
| Ranidae | *Staurois natator* | 45.8 | 36.0 | 0.27 | M | 1.9 | - | 0 | 0 | 0 | 0 | 0 | 0 | 0 |
| Rhacophoridae | *Buergeria buergeri* | 61.2 | 36.5 | 0.68 | M | 1.3 | - | - | - | - | - | 0 | 0 | 0 |
| Rhacophoridae | *Buergeria japonica* | 34.9 | 29.9 | 0.17 | M | 1.3 | 600 | 0 | 0 | 0 | 0 | 0 | 0 | 0 |
| Rhacophoridae | *Buergeria oxycephala* | 64.5 | 36.5 | 0.77 | M | 1.5 | - | - | - | - | - | 0 | 0 | 0 |
| Rhacophoridae | *Buergeria robusta* | 67.1 | 50.2 | 0.34 | M | 2.5 | - | 0 | 0 | 0 | 0 | 0 | 0 | 0 |
| Rhacophoridae | *Chirixalus doriae* | 27.4 | 21.6 | 0.27 | M | 1.4 | 180 | 0 | 0 | 0 | 0 | 0 | 0 | 0 |
| Rhacophoridae | *Chirixalus vittatus* | 25.7 | 23.6 | 0.09 | M | 1.5 | 217 | 0 | 0 | 0 | 0 | 0 | 0 | 0 |
| Rhacophoridae | *Chiromantis xerampelina* | - | - | - | - | 1.8 | 192 | 1 | 0 | 1 | 0 | 0 | 1 | 1 |
| Rhacophoridae | *Gracixalus gracilipes* | 29.5 | 22.0 | 0.34 | R | 2.7 | - | 0 | 0 | 0 | 0 | 0 | 0 | 0 |
| Rhacophoridae | *Kurixalus eiffingeri* | 38.0 | 32.8 | 0.16 | M | 1.9 | 79 | 0 | 0 | 0 | 0 | 1 | 1 | 1 |
| Rhacophoridae | *Philautus aurifasciatus* | 31.6 | 23.8 | 0.33 | M | 2.7 | 13 | - | - | - | - | 0 | 0 | 0 |
| Rhacophoridae | *Polypedates leucomystax* | 62.7 | 43.5 | 0.44 | M | 1.5 | 337 | 0 | 0 | 0 | 0 | 0 | 0 | 0 |
| Rhacophoridae | *Polypedates megacephalus* | 67.1 | 49.0 | 0.37 | M | 1.9 | 362 | 0 | 0 | 0 | 0 | 1 | 1 | 1 |
| Rhacophoridae | *Polypedates mutus* | 66.7 | 53.4 | 0.25 | M | 2.0 | - | 0 | 0 | 0 | 0 | 0 | 0 | 0 |
| Rhacophoridae | *Rhacophorus arboreus* | 81.4 | 60.3 | 0.35 | M | 3.2 | 288 | - | - | - | - | 0 | 0 | 0 |
| Rhacophoridae | *Rhacophorus bipunctatus* | - | - | - | - | - | - | 0 | 0 | 0 | 0 | 0 | 0 | 0 |
| Rhacophoridae | *Rhacophorus dennysi* | 100.2 | 87.5 | 0.15 | M | 2.0 | 2625 | 0 | 0 | 0 | 0 | 0 | 0 | 0 |
| Rhacophoridae | *Rhacophorus dugritei* | 56.7 | 45.0 | 0.26 | M | 2.5 | 400 | 0 | 0 | 0 | 0 | 0 | 0 | 0 |
| Rhacophoridae | *Rhacophorus feae* | 92.3 | 111.0 | -0.17 | R | - | - | 0 | 0 | 0 | 0 | 0 | 0 | 0 |
| Rhacophoridae | *Rhacophorus kio* | 81.6 | 67.9 | 0.20 | M | 3.0 | - | 1 | 0 | 1 | 0 | 0 | 0 | 0 |
| Rhacophoridae | *Rhacophorus moltrechti* | 49.8 | 42.1 | 0.18 | M | 2.5 | 180 | 0 | 0 | 0 | 0 | 0 | 0 | 0 |
| Rhacophoridae | *Rhacophorus nigropunctatus* | 44.5 | 37.5 | 0.19 | R | 1.8 | - | 0 | 0 | 0 | 0 | 0 | 0 | 0 |
| Rhacophoridae | *Rhacophorus omeimontis* | 75.7 | 59.0 | 0.28 | M | 3.3 | - | 0 | 0 | 0 | 0 | 0 | 1 | 1 |
| Rhacophoridae | *Rhacophorus reinwardtii* | 82.7 | 68.0 | 0.22 | M | 3.0 | 68 | 0 | 0 | 0 | 0 | 0 | 0 | 0 |
| Rhacophoridae | *Rhacophorus rhodopus* | 48.7 | 35.0 | 0.39 | M | - | 125 | 0 | 0 | 0 | 0 | 0 | 0 | 0 |
| Rhacophoridae | *Rhacophorus schlegelii* | 57.0 | 41.3 | 0.38 | M | 2.5 | 475 | - | - | - | - | 0 | 0 | 0 |
| Rhinophrynidae | *Rhinophrynus dorsalis* | - | - | - | - | 4.5 | 5000 | 0 | 0 | 0 | 0 | 0 | 0 | 0 |
| Scaphiopodidae | *Scaphiopus couchii* | - | - | - | - | 1.5 | 3310 | 0 | 0 | 0 | 0 | 0 | 0 | 0 |
| Scaphiopodidae | *Scaphiopus holbrookii* | - | - | - | - | 1.7 | - | 0 | 0 | 0 | 0 | 0 | 0 | 0 |
| Scaphiopodidae | *Scaphiopus hurterii* | 63.0 | 58.0 | 0.09 | M | 2.3 | - | 0 | 0 | 0 | 0 | 0 | 0 | 0 |
| Scaphiopodidae | *Spea bombifrons* | 48.5 | 45.0 | 0.08 | M | - | 462 | 0 | 0 | 0 | 0 | 0 | 0 | 0 |
| Scaphiopodidae | *Spea hammondii* | 49.3 | 48.3 | 0.02 | M | 1.6 | 400 | 0 | 0 | 0 | 0 | 0 | 0 | 0 |
| Scaphiopodidae | *Spea intermontana* | 57.2 | 50.5 | 0.13 | M | - | 325 | 0 | 0 | 0 | 0 | 0 | 0 | 0 |
| Sooglossidae | *Sooglossus sechellensis* | - | - | - | - | - | - | - | - | - | - | 1 | 0 | 1 |
| Strabomantidae | *Oreobates quixensis* | 57.6 | 43.8 | 0.32 | M | 4.0 | 35 | - | - | - | - | 0 | 0 | 0 |
| Strabomantidae | *Strabomantis bufoniformis* | - | - | - | - | - | - | 0 | 0 | 0 | 0 | - | - | - |
