# Supplementary material for: Does life history shape sexual size dimorphism in anurans? A comparative analysis
Source: BMC Evol Biol. 2013 Jan 31;13:27. doi: 10.1186/1471-2148-13-27 (PMC3570426; doi:10.1186/1471-2148-13-27)
Supplement: Additional file 2 — References for body size, egg size, clutch size, mating combat, and parental care in 688 anuran species. The general mating or breeding descriptions are used to define the absence of mating combat or parental care behaviour, respectively. [file 1471-2148-13-27-S2.docx]

**Additional file 2: References for body size, egg size, clutch size, mating combat, and parental care in 688 anuran species.** The general mating or breeding descriptions are used to define the absence of mating combat or parental care behaviour, respectively.

| **Family** | **Species** | **Body Size** | **Egg Size** | **Clutch Size** | **Mating Combat** | **Mating Descriptions** | **Parental Care** | **Breeding Descriptions** |
| --- | --- | --- | --- | --- | --- | --- | --- | --- |
| Allophrynidae | *Allophryne ruthveni* | Duellman, 1997 | N/A | Duellman, 1997 | N/A | Duellman, 1997 | N/A | AmphibiaWeb |
| Alytidae | *Alytes obstetricans* | AmphibiaWeb | Summers, 2006 | Bush, 1996;  Marquez, 1993 | Eens, 2000 | N/A | Duellman, 1994 | N/A |
| Alytidae | *Discoglossus pictus* | N/A | AmphibiaWeb | AmphibiaWeb | N/A | AmphibiaWeb | N/A | AmphibiaWeb |
| Aromobatidae | *Allobates brunneus* | Caldwells, 2003 | N/A | N/A | N/A | N/A | N/A | N/A |
| Aromobatidae | *Allobates caeruleodactylus* | Caldwells, 2003 | N/A | N/A | Lima, 2002 | N/A | Lima, 2002 | N/A |
| Aromobatidae | *Allobates femoralis* | Crump, 1974;  Silverstone, 1976 | Crump, 1974 | Crump, 1974 | Narin, 2004 | N/A | Rodriguez, 1994 | N/A |
| Aromobatidae | *Allobates nidicola* | Caldwells, 2003 | Caldwells, 2003 | Caldwells, 2003 | N/A | Caldwells, 2003 | N/A | Caldwells, 2003 |
| Aromobatidae | *Allobates talamancae* | N/A | N/A | Savage, 2002 | Prohl, 2005 | N/A | Duellman, 2004 | N/A |
| Aromobatidae | *Allobates zaparo* | Silverstone, 1976 | N/A | N/A | N/A | N/A | AmphibiaWeb | N/A |
| Aromobatidae | *Anomaloglossus beebei* | N/A | Bourne, 2001 | Bourne, 2001 | N/A | Prohl, 2005 | Bourne, 2001 | N/A |
| Aromobatidae | *Anomaloglossus stepheni* | Caldwells, 2003 | N/A | Junca, 2006 | Junca, 2006 | N/A | Junca, 2006 | N/A |
| Aromobatidae | *Mannophryne trinitatis* | Sexton, 1960 | N/A | Jowers, 2005 | Sexton, 1960 | N/A | Duellman, 1994 | N/A |
| Aromobatidae | *Rheobates palmatus* | N/A | Stebbins, 1959 | Stebbins, 1959 | N/A | Bernal, 2005 | Duellman, 1994 | N/A |
| Arthroleptidae | *Trichobatrachus robustus* | AmphibiaWeb | N/A | N/A | N/A | AmphibiaWeb | Beck, 1998 | N/A |
| Bombinatoridae | *Barbourula busuangensis* | Inger, 1954 | Inger, 1954 | Inger, 1954 | N/A | N/A | N/A | N/A/ |
| Bombinatoridae | *Bombina bombina* | N/A | Kuzmin, 1999 | Kuzmin, 1999 | Kuzmin, 1999 | N/A | N/A | N/A |
| Bombinatoridae | *Bombina fortinuptialis* | Fei, 2009 | Fei, 2009 | N/A | N/A | N/A | N/A | N/A |
| Bombinatoridae | *Bombina lichuanensis* | Fei, 2009 | Fei, 2009 | N/A | N/A | N/A | N/A | N/A |
| Bombinatoridae | *Bombina maxima* | Yang, 1991 | N/A | N/A | N/A | Fei, 1999 | N/A | N/A |
| Bombinatoridae | *Bombina orientalis* | Okada, 1966 | Kuzmin, 1999 | Kuzmin, 1999 | Kuzmin, 1999 | N/A | N/A | N/A |
| Bombinatoridae | *Bombina variegata* | N/A | N/A | Kuzmin, 1999 | Seidel, 1999 | N/A | N/A | N/A |
| Brevicipitidae | *Breviceps mossambicus* | Stewart, 1967 | Stewart, 1967 | Stewart, 1967 | N/A | AmphibiaWeb | N/A | N/A |
| Bufonidae | *Amietophrynus garm* | Channing, 2001 | Summers, 2006 | N/A | N/A | Keith, 1968 | N/A | N/A |
| Bufonidae | *Amietophrynus gutturalis* | N/A | Summers, 2006 | N/A | Telford, 1989 | Passmore, 1981 | N/A | N/A |
| Bufonidae | *Amietophrynus kisoloensis* | Loveridge, 1936 | N/A | N/A | N/A | Keith, 1968 | N/A | N/A |
| Bufonidae | *Amietophrynus maculatus* | Stewart, 1967 | Summers, 2006 | N/A | Channing, 2001 | N/A | N/A | N/A |
| Bufonidae | *Amietophrynus pardalis* | Cherry, 1992 | N/A | N/A | Cherry, 1992 | N/A | N/A | N/A |
| Bufonidae | *Amietophrynus regularis* | Loveridge, 1936 | Wager, 1965 | Wager, 1965 | Tandy, 1972 | N/A | N/A | N/A |
| Bufonidae | *Amietophrynus xeros* | N/A | N/A | N/A | N/A | Tandy, 1976 | N/A | N/A |
| Bufonidae | *Anaxyrus americanus* | Wilbur, 1978 | Wright, 1995 | Wright, 1995 | Howard, 1988 | N/A | N/A | Wright, 1995 |
| Bufonidae | *Anaxyrus boreas* | Wright, 1995 | Wright, 1995 | Degenhardt, 1996 | Olson, 1986 | N/A | N/A | Wright, 1995 |
| Bufonidae | *Anaxyrus californicus* | Wright, 1995 | Wright, 1995 | AmphibiaWeb | N/A | AmphibiaWeb | N/A | N/A |
| Bufonidae | *Anaxyrus canorus* | Wright, 1995 | Summers, 2006 | N/A | N/A | Wright, 1995 | N/A | N/A |
| Bufonidae | *Anaxyrus cognatus* | Wright, 1995 | Stebbins, 1951;  Wright, 1995 | Degenhardt, 1996 | Sullivan, 1983 | Degenhardt, 1996 | N/A | N/A |
| Bufonidae | *Anaxyrus debilis* | Bogert, 1962 | Summers, 2006 | N/A | N/A | Degenhardt, 1996 | N/A | N/A |
| Bufonidae | *Anaxyrus exsul* | Murphy, 2003 | AmphibiaWeb | N/A | Schuierer, 1962 | N/A | N/A | N/A |
| Bufonidae | *Anaxyrus fowleri* | Hulse, 2001 | N/A | Hulse, 2001 | N/A | Given, 2002 | N/A | Wright, 1995 |
| Bufonidae | *Anaxyrus hemiophrys* | Wright, 1995 | N/A | N/A | N/A | Cocroft, 1995 | N/A | N/A |
| Bufonidae | *Anaxyrus microscaphus* | N/A | Stebbins, 1951 | Degenhardt, 1996 | N/A | Degenhardt, 1996 | N/A | N/A |
| Bufonidae | *Anaxyrus nelsoni* | Wright, 1995 | N/A | N/A | N/A | Wright, 1995 | N/A | Wright, 1995 |
| Bufonidae | *Anaxyrus punctatus* | Wright, 1995 | Wright, 1995 | N/A | N/A | Degenhardt, 1996 | N/A | Wright, 1995 |
| Bufonidae | *Anaxyrus quercicus* | Wilbur, 1978 | Wright, 1995 | N/A | N/A | Wright, 1995 | N/A | N/A |
| Bufonidae | *Anaxyrus retiformis* | Bogert, 1962 | N/A | N/A | N/A | Bogert, 1962 | N/A | N/A |
| Bufonidae | *Anaxyrus terrestris* | Wright, 1995 | Wright, 1995 | Wright, 1995 | N/A | Wright, 1995 | N/A | Wright, 1995 |
| Bufonidae | *Anaxyrus woodhousii* | Wright, 1995 | Stebbins, 1951 | Degenhardt, 1996 | Degenhardt, 1996 | N/A | N/A | N/A |
| Bufonidae | *Ansonia malayana* | Dring, 1979 | N/A | N/A | N/A | Dring, 1979 | N/A | N/A |
| Bufonidae | *Atelopus chiriquiensis* | Savage, 2002 | Savage, 2002 | Savage, 2002 | Savage, 2002 | N/A | N/A | Savage, 2002 |
| Bufonidae | *Atelopus senex* | Savage, 1972 | N/A | N/A | N/A | Savage, 2002 | N/A | N/A |
| Bufonidae | *Atelopus spumarius* | Rodriguez, 1994 | Summers, 2006 | N/A | N/A | N/A | N/A | Summers, 2006 |
| Bufonidae | *Atelopus varius* | Savage, 2002 | Savage, 2002 | Savage, 2002 | Savage, 2002 | N/A | N/A | Savage, 2002 |
| Bufonidae | *Bufo aspinius* | Fei, 2009 | Fei, 2009 | Fei, 2009 | Fei, 2009 | N/A | N/A | Fei, 2009 |
| Bufonidae | *Bufo bankorensis* | Schmidt, 1927 | Pope, 1931 | Pope, 1931 | N/A | Pope, 1931 | N/A | N/A |
| Bufonidae | *Bufo bufo* | Beebee, 2000 | Kuzmin, 1999 | Beebee, 2000;  Hettyey, 2005 | Davies, 1979 | N/A | N/A | Hettyey, 2005 |
| Bufonidae | *Bufo gargarizans* | Yang, 1991 | Fei, 1999 | N/A | N/A | AmphibiaWeb | N/A | N/A |
| Bufonidae | *Bufo mauritanicus* | N/A | Summers, 2006 | Batraciens et reptiles du monde | N/A | Batraciens et reptiles du monde | N/A | N/A |
| Bufonidae | *Bufo stejnegeri* | Fei, 2009 | N/A | Fei, 2009 | N/A | Fei, 2009 | N/A | Fei, 2009 |
| Bufonidae | *Bufo tibetanus* | Fei, 2009 | Fei, 2009 | Fei, 2009 | N/A | Fei, 2009 | N/A | Fei, 2009 |
| Bufonidae | *Bufo verrucosissimus* | Kuzmin, 1999 | Kuzmin, 1999 | Kuzmin, 1999 | N/A | Kuzmin, 1999 | N/A | N/A |
| Bufonidae | *Capensibufo rosei* | Channing, 2001 | Wager, 1965 | N/A | N/A | N/A | N/A | Summers, 2006 |
| Bufonidae | *Capensibufo tradouwi* | Channing, 2001 | Summers, 2006 | N/A | N/A | N/A | N/A | Summers, 2006 |
| Bufonidae | *Dendrophryniscus minutus* | Rodriquez, 1994 | Crump, 1974 | Crump, 1974 | N/A | N/A | N/A | N/A |
| Bufonidae | *Duttaphrynus himalayanus* | Yang, 1991  Hu, 1987 | Hu, 1987 | N/A | N/A | N/A | N/A | N/A |
| Bufonidae | *Duttaphrynus melanostictus* | Inger, 1966 | N/A | N/A | N/A | Marquez, 2006 | N/A | N/A |
| Bufonidae | *Incilius alvarius* | Wright, 1995 | Wright, 1995 | Wright, 1995 | Degenhardt, 1996 | N/A | N/A | N/A |
| Bufonidae | *Incilius coccifer* | Mendelson,2005 | Savage,2002 | Savage,2002 | Savage,2002 | N/A | N/A | Savage,2002 |
| Bufonidae | *Incilius coniferus* | Savage, 2002 | Savage, 2002 | N/A | N/A | Savage, 2002 | N/A | Savage, 2002 |
| Bufonidae | *Incilius fastidiosus* | Savage, 1972 | Savage, 2002 | Savage, 2002 | Savage, 2002 | N/A | N/A | Savage, 2002 |
| Bufonidae | *Incilius ibarrai* | N/A | N/A | N/A | N/A | N/A | N/A | N/A |
| Bufonidae | *Incilius luetkenii* | Savage, 2002 | Savage, 2002 | Savage, 2002 | N/A | Savage, 2002 | N/A | Savage, 2002 |
| Bufonidae | *Incilius melanochlorus* | Savage, 2002 | N/A | N/A | N/A | Savage, 2002 | N/A | N/A |
| Bufonidae | *Incilius nebulifer* | Stebbins, 1959 | Summers, 2006 | Cei, 1980 | N/A | Cei, 1980 | N/A | Cei, 1980 |
| Bufonidae | *Incilius valliceps* | Wright, 1995 | Savage, 2002;  Wright, 1995 | N/A | N/A | Wells, 1977 | N/A | N/A |
| Bufonidae | *Ingerophrynus biporcatus* | Inger, 1966 | N/A | N/A | N/A | Marquez, 2006 | N/A | N/A |
| Bufonidae | *Mertensophryne taitana* | Stewart, 1967 | Summers, 2006 | N/A | N/A | Stewart, 1967 | N/A | N/A |
| Bufonidae | *Nannophryne variegata* | N/A | N/A | Cei, 1980 | N/A | Cei, 1980 | N/A | Cei, 1980 |
| Bufonidae | *Peltophryne fustiger* | Valdes, 1988 | Schwartz, 1991 | N/A | N/A | Schwartz, 1991 | N/A | N/A |
| Bufonidae | *Peltophryne peltocephala* | Valdes, 1988 | N/A | N/A | N/A | Valdes, 1988 | N/A | N/A |
| Bufonidae | *Phrynoidis asper* | Inger, 1966 | Inger, 1968 | Inger, 1968 | N/A | N/A | N/A | N/A |
| Bufonidae | *Phrynoidis juxtaspera* | Inger, 1966 | N/A | N/A | N/A | AmphibiaWeb | N/A | N/A |
| Bufonidae | *Pseudepidalea pewzowi* | Fei, 2009 | Fei, 2009 | Fei, 2009 | N/A | Fei, 2009 | N/A | Fei, 2009 |
| Bufonidae | *Pseudepidalea viridis* | N/A | Kuzmin, 1999 | Kuzmin, 1999 | N/A | AmphibiaWeb | N/A | N/A |
| Bufonidae | *Rhaebo glaberrimus* | Bartlett, 2003 | N/A | N/A | N/A | AmphibiaWeb | N/A | N/A |
| Bufonidae | *Rhaebo haematiticus* | Savage, 2002 | N/A | N/A | N/A | Savage, 2002 | N/A | Savage, 2002 |
| Bufonidae | *Rhinella arenarum* | Cei, 1980 | N/A | Cei, 1980 | N/A | Penna, 1990 | N/A | N/A |
| Bufonidae | *Rhinella castaneotica* | Caramaschi, 2003 | Caldwells, 2004 | Caldwells, 2004 | N/A | Caldwells, 2004 | N/A | Caldwells, 2004 |
| Bufonidae | *Rhinella chavin* | Lehr, 2001 | Lehr, 2001 | Lehr, 2001 | N/A | Lehr, 2001 | N/A | N/A |
| Bufonidae | *Rhinella dapsilis* | Rodriguez, 1994 | N/A | N/A | N/A | N/A | N/A | N/A |
| Bufonidae | *Rhinella margaritifera* | Duellman, 1996 | Crump, 1974 | Crump, 1974 | Cherry, 1992 | N/A | N/A | N/A |
| Bufonidae | *Rhinella marina* | Stebbins, 1959 | Crump, 1974;  Savage, 2002;  Stebbins, 1959 | Crump, 1974 | Savage, 2002 | N/A | N/A | Savage, 2002;  Wright, 1995 |
| Bufonidae | *Rhinella schneideri* | N/A | Cei, 1980 | N/A | N/A | Cei, 1980 | N/A | Cei, 1980 |
| Bufonidae | *Rhinella spinulosa* | N/A | N/A | N/A | N/A | Penna 1990 | N/A | N/A |
| Bufonidae | *Schismaderma carens* | Stewart, 1967 | Stewart, 1967;  Wager, 1965 | Stewart, 1967;  Wager, 1965 | N/A | AmphibiaWeb | N/A | N/A |
| Centrolenidae | *Centrolene geckoideum* | Lynch, 1973 | Lynch, 1973 | Lynch, 1973 | N/A | Grant, 1998 | Lynch, 1973 | N/A |
| Centrolenidae | *Centrolenella prosoblepon* | Lynch, 1973 | N/A | Savage, 2002  Kubicki, 2007 | Savage, 2002  Kubicki, 2007 | N/A | Zug, 2001 | N/A |
| Centrolenidae | *Cochranella euknemos* | Savage, 2002  Kubicki, 2007 | N/A | Kubicki, 2007 | N/A | Savage, 2002 | N/A | Savage, 2002 |
| Centrolenidae | *Cochranella granulosa* | Savage, 2002 | Savage, 2002 | Kubicki, 2007 | Kubicki, 2007 | Savage, 2002 | N/A | Savage, 2002 |
| Centrolenidae | *Hyalinobatrachium chirripoi* | Kubicki, 2007 | N/A | Kubicki, 2007 | N/A | Kubicki, 2007 | Kubicki, 2007 | N/A |
| Centrolenidae | *Hyalinobatrachium colymbiphyllum* | Savage, 2002  Kubicki, 2007 | Savage, 2002 | Savage, 2002  Kubicki, 2007 | Savage, 2002 | N/A | Savage, 2002  Kubicki, 2007 | N/A |
| Centrolenidae | *Hyalinobatrachium fleischmanni* | Savage, 2002 | Savage, 2002 | Savage, 2002  Kubicki, 2007 | Savage, 2002  Kubicki, 2007 | Savage, 2002 | Savage, 2002  Kubicki, 2007 | N/A |
| Centrolenidae | *Hyalinobatrachium talamancae* | Kubicki, 2007 | N/A | Kubicki, 2007 | N/A | Kubicki, 2007 | Kubicki, 2007 | N/A |
| Centrolenidae | *Hyalinobatrachium valerioi* | Savage, 2002  Kubicki, 2007 | Savage, 2002 | Savage, 2002  Kubicki, 2007 | Savage, 2002  Kubicki, 2007 | N/A | Savage, 2002  Kubicki, 2007 | N/A |
| Centrolenidae | *Nymphargus griffithsi* | Lynch, 1973 | N/A | N/A | Duellman, 1994 | N/A | N/A | N/A |
| Centrolenidae | *Sachatamia albomaculata* | Savage, 2002  Kubicki, 2007 | N/A | N/A | N/A | Savage, 2002 | N/A | N/A |
| Centrolenidae | *Sachatamia ilex* | Savage, 2002  Kubicki, 2007 | N/A | Kubicki, 2007 | Kubicki, 2007 | Savage, 2002 | N/A | N/A |
| Centrolenidae | *Teratohyla midas* | Lynch, 1973 | Crump, 1974 | Crump, 1974 | N/A | N/A | N/A | N/A |
| Centrolenidae | *Teratohyla pulverata* | Savage, 2002 | N/A | Savage, 2002 | N/A | Savage, 2002 | N/A | Savage, 2002  Kubicki, 2007 |
| Centrolenidae | *Teratohyla spinosa* | Savage, 2002  Kubicki, 2007 | N/A | Kubicki, 2007 | N/A | Savage, 2002 | N/A | N/A |
| Ceratobatrachidae | *Ingerana baluensis* | Inger, 1966 | Inger, 1966 | N/A | N/A | N/A | N/A | N/A |
| Craugastoridae | *Craugastor augusti* | N/A | N/A | Degenhardt, 1996;  Wright, 1995 | N/A | Stebbins, 2003 | Duellman, 1994 | N/A |
| Craugastoridae | *Haddadus binotatus* | N/A | N/A | N/A | N/A | N/A | N/A | AmphibiaWeb |
| Cycloramphidae | *Rhinoderma darwinii* | AmphibiaWeb | The Web Site of Huina-pukios | AmphibiaWeb | N/A | AmphibiaWeb | AmphibiaWeb | N/A |
| Dendrobatidae | *Adelphobates galactonotus* | Silverstone, 1975 | N/A | N/A | N/A | N/A | N/A | N/A |
| Dendrobatidae | *Adelphobates quinquevittatus* | Silverstone, 1975;  Myers, 1982 | N/A | N/A | N/A | Zimmermann, 1988 | Beck, 1998 | N/A |
| Dendrobatidae | *Ameerega hahneli* | Rodriguez, 1994 | Rodriguez, 1994 | Rodriguez, 1994 | N/A | Gottsberger, 2004 | www.poison-  frogs.com | N/A |
| Dendrobatidae | *Ameerega parvula* | Silverstone, 1976;  Crump, 1974 | Crump, 1974 | Crump, 1974 | N/A | Zug, 2001 | Rodriguez, 1994 | N/A |
| Dendrobatidae | *Ameerega petersi* | Silverstone, 1976 | N/A | N/A | N/A | N/A | Beck, 1998 | N/A |
| Dendrobatidae | *Ameerega picta* | Silverstone, 1976;  Crump, 1974 | Crump, 1974 | Crump, 1974 | N/A | N/A | Beck, 1998 | N/A |
| Dendrobatidae | *Ameerega pulchripecta* | Silverstone, 1976 | N/A | N/A | N/A | N/A | N/A | N/A |
| Dendrobatidae | *Ameerega silverstonei* | Myers, 1979 | Summers, 2006 | Myers, 1979 | N/A | Zimmermann, 1988 | Myers, 1979 | N/A |
| Dendrobatidae | *Ameerega trivittata* | Silverstone, 1976;  Duellman, 1996 | Roithmair, 1994 | Myers, 1979;  Rodriguez, 1994 | Roithmair, 1994;  Silverstone, 1976 | N/A | Rodriguez, 1994;  Summers, 1999a | N/A |
| Dendrobatidae | *Colostethus inguinalis* | N/A | N/A | N/A | Duellman, 1966 | N/A | Duellman, 1966 | N/A |
| Dendrobatidae | *Dendrobates auratus* | Silverstone, 1975;  Summers, 1989 | Summers, 2006 | Dunn, 1941;  Savage, 2002 | Eens, 2000 | N/A | Duellman, 1994 | N/A |
| Dendrobatidae | *Dendrobates leucomelas* | Silverstone, 1975;  Summers, 1989 | Summers, 2006 | Summers, 1992a | Summers, 1992b | N/A | Summers, 1992a | N/A |
| Dendrobatidae | *Dendrobates tinctorius* | Silverstone, 1975 | N/A | Summers, 1999b | N/A | N/A | Summers, 1999b | N/A |
| Dendrobatidae | *Dendrobates truncatus* | Silverstone, 1975 | N/A | Summers, 1999b | N/A | N/A | Summers, 1999b | N/A |
| Dendrobatidae | *Epipedobates anthonyi* | Silverstone, 1976 | N/A | N/A | N/A | N/A | Beck, 1998 | N/A |
| Dendrobatidae | *Epipedobates boulengeri* | Silverstone, 1976 | N/A | N/A | N/A | N/A | Beck, 1998 | N/A |
| Dendrobatidae | *Epipedobates espinosai* | N/A | N/A | N/A | N/A | N/A | Beck, 1998 | N/A |
| Dendrobatidae | *Epipedobates tricolor* | Duellman, 1993;  Silverstone, 1976 | N/A | www.poison-  frogs.com | Hermans, 2002 | N/A | www.poison-  frogs.com | N/A |
| Dendrobatidae | *Hyloxalus awa* | Coloma, 1995 | N/A | Coloma, 1995 | Coloma, 1995 | N/A | Coloma, 1995 | N/A |
| Dendrobatidae | *Hyloxalus elachyhistus* | Duellman, 1993 | N/A | Edwards, 1971 | N/A | N/A | Coloma, 1995 | N/A |
| Dendrobatidae | *Hyloxalus idiomelus* | Duellman, 2004 | N/A | N/A | N/A | N/A | N/A | N/A |
| Dendrobatidae | *Hyloxalus insulatus* | Duellman, 2004 | N/A | N/A | N/A | N/A | N/A | N/A |
| Dendrobatidae | *Hyloxalus nexipus* | Coloma, 1995;  Duellman, 2004 | N/A | N/A | N/A | N/A | N/A | N/A |
| Dendrobatidae | *Hyloxalus subpunctatus* | Stebbins, 1959 | Stebbins, 1959 | Stebbins, 1959 | N/A | Navas, 2001 | Stebbins, 1959 | N/A |
| Dendrobatidae | *Hyloxalus sylvaticus* | Duellman, 1993;  Duellman, 2004 | N/A | N/A | N/A | N/A | N/A | N/A |
| Dendrobatidae | *Hyloxalus vertebralis* | Coloma, 1995;  Edwards, 1971 | N/A | N/A | N/A | Edwards, 1971 | Global Amphibian Assessment | N/A |
| Dendrobatidae | *Oophaga granulifera* | Silverstone, 1975 | N/A | Savage, 2002 | Savage, 2002 | N/A | Savage, 2002;  Summers, 1999a | N/A |
| Dendrobatidae | *Oophaga histrionica* | Silverstone, 1975;  Summers, 1989;  Myers, 1996 | Summers, 2006 | N/A | Summers, 1992b | N/A | Duellman, 1994;  Summers, 1992b | N/A |
| Dendrobatidae | *Oophaga lehmanni* | Myers,1976 | Summers,2006 | N/A | Myers, 1976 | N/A | Duellman,1994; Summers,1989 | N/A |
| Dendrobatidae | *Oophaga pumilio* | Silverstone, 1975;  Summers, 1989 | Summers, 2006 | Prohl, 2005 | Wells, 1977 | N/A | Summers, 1999a;  Duellman, 1994 | N/A |
| Dendrobatidae | *Oophaga speciosa* | Silverstone, 1975 | N/A | N/A | Jungfer, 1985 | N/A | Summers, 1999b | N/A |
| Dendrobatidae | *Phyllobates aurotaenia* | Silverstone, 1976 | N/A | N/A | N/A | N/A | N/A | N/A |
| Dendrobatidae | *Phyllobates bicolor* | Silverstone, 1976;  Myers, 1996;  Myers, 1978 | N/A | Animal Diversity Web | N/A | Animal Diversity Web | Global Amphibian Assessment | N/A |
| Dendrobatidae | *Phyllobates lugubris* | Silverstone, 1976 | N/A | www.poison-  frogs.com | N/A | Savage, 2002 | Savage, 2002 | N/A |
| Dendrobatidae | *Phyllobates terribilis* | Myers, 1978 | Myers, 1978 | Myers, 1978 | Zimmermann, 1985 | N/A | Zimmermannn, 1985 | N/A |
| Dendrobatidae | *Phyllobates vittatus* | Silverstone, 1976 | Summers, 2006 | Savage, 2002 | Silverstone, 1976;  Summers, 2000 | N/A | Duellman, 1994 | N/A |
| Dendrobatidae | *Ranitomeya fantastica* | N/A | N/A | www.poison-  frogs.com | N/A | N/A | Summers, 1999a | N/A |
| Dendrobatidae | *Ranitomeya fulgurita* | Silverstone, 1975 | N/A | N/A | N/A | N/A | N/A | N/A |
| Dendrobatidae | *Ranitomeya imitator* | Brown, 2008 | N/A | Brown, 2008 | Brown, 2008 | Zimmermann, 1988 | Brown, 2008 | N/A |
| Dendrobatidae | *Ranitomeya minuta* | Silverstone, 1975 | N/A | N/A | N/A | Myers, 1976 | Beck, 1998 | N/A |
| Dendrobatidae | *Ranitomeya reticulata* | Myers, 1982 | Rodriguez, 1994 | Rodriguez, 1994 | N/A | Myers, 1982 | Rodriguez, 1994 | N/A |
| Dendrobatidae | *Ranitomeya vanzolinii* | Myers, 1982 | N/A | www.poison-  frogs.com | N/A | Caldwells, 1999;  Prohl, 2005 | Caldwells, 1999 | N/A |
| Dendrobatidae | *Ranitomeya variabilis* | Brown, 2008 | N/A | Brown, 2008 | Brown, 2008 | Zimmermann, 1988 | Brown, 2008 | N/A |
| Dendrobatidae | *Ranitomeya ventrimaculata* | Rodriguez, 1994 | Summers, 2006 | Poelman, 2006 | Poelman, 2006 | N/A | Poelman, 2006 | N/A |
| Dendrobatidae | *Silverstoneia flotator* | Savage, 2002 | N/A | N/A | Savage, 2002 | N/A | Savage, 2002 | N/A |
| Dendrobatidae | *Silverstoneia nubicola* | Savage, 2002 | N/A | N/A | N/A | Summers, 2000;  Prohl, 2005 | Duellman, 1994 | N/A |
| Dicroglossidae | *Euphlyctis cyanophlyctis* | Gramapurohit, 2005 | N/A | N/A | N/A | AmphibiaWeb | N/A | AmphibiaWeb |
| Dicroglossidae | *Fejervarya cancrivora* | Inger, 1966 | Ye, 1993 | N/A | N/A | Inger, 1966 | N/A | Alcala, 1986 |
| Dicroglossidae | *Fejervarya limnocharis* | Inger, 1966 | Ye, 1993 | Chen, 1991;  Ye, 1993 | Ye, 1993 | N/A | N/A | Ye, 1993 |
| Dicroglossidae | *Hoplobatrachus occipitalis* | AmphibiaWeb | AmphibiaWeb | N/A | N/A | AmphibiaWeb | N/A | AmphibiaWeb |
| Dicroglossidae | *Hoplobatrachus rugulosus* | Chen, 1991 | Huang, 1990;  Ye, 1993 | Huang, 1990 | N/A | Huang, 1990 | N/A | N/A |
| Dicroglossidae | *Limnonectes blythii* | N/A | Summers, 2006 | Inger, 1968 | Emerson, 1992 | N/A | Summers, 2006;  AmphibiaWeb | N/A |
| Dicroglossidae | *Limnonectes finchi* | N/A | Summers, 2006 | N/A | N/A | N/A | Beck, 1998 | N/A |
| Dicroglossidae | *Limnonectes gyldenstolpei* | Ohler, 2002 | N/A | N/A | N/A | AmphibiaWeb | N/A | N/A |
| Dicroglossidae | *Limnonectes ibanorum* | N/A | N/A | Inger, 1968 | N/A | N/A | N/A | N/A |
| Dicroglossidae | *Limnonectes kuhlii* | Yang, 1991 | Chen, 1991;  Huang, 1990;  Pope, 1931 | Chen, 1991 | Tsuji, 2004 | N/A | N/A | AmphibiaWeb |
| Dicroglossidae | *Limnonectes laticeps* | Dring, 1979 | N/A | N/A | N/A | Dring, 1979 | N/A | N/A |
| Dicroglossidae | *Limnonectes macrodon* | Inger, 1966 | Summers, 2006 | Inger, 1966 | N/A | Inger, 1966 | N/A | N/A |
| Dicroglossidae | *Limnonectes microdiscus* | Inger, 1966 | Summers, 2006 | Inger, 1966 | N/A | N/A | Inger, 1966 | N/A |
| Dicroglossidae | *Limnonectes palavanensis* | N/A | Summers, 2006 | N/A | N/A | N/A | Beck, 1998;  Summers, 2006 | N/A |
| Dicroglossidae | *Limnonectes paramacrodon* | Inger, 1966 | N/A | N/A | N/A | AmphibiaWeb | N/A | N/A |
| Dicroglossidae | *Limnonectes parvus* | N/A | Inger, 1954 | Inger, 1954 | N/A | N/A | N/A | N/A |
| Dicroglossidae | *Nannophrys ceylonensis* | Wickramasinghe, 2004 | N/A | N/A | Wickramasinghe, 2004 | N/A | Wickramasinghe, 2004 | N/A |
| Dicroglossidae | *Nanorana parkeri* | Hu, 1987 | Ye, 1993 | N/A | N/A | Hu, 1987 | N/A | N/A |
| Dicroglossidae | *Nanorana pleskei* | Ye, 1993 | N/A | N/A | N/A | AmphibiaWeb | N/A | N/A |
| Dicroglossidae | *Nanorana unculuanus* | Yang, 1991 | Ye, 1993 | Yang, 1991 | N/A | AmphibiaWeb | N/A | N/A |
| Dicroglossidae | *Nanorana yunnanensis* | Pope, 1931 | Yang, 1991;  Ye, 1993 | Ye, 1993 | N/A | AmphibiaWeb | N/A | AmphibiaWeb |
| Dicroglossidae | *Occidozyga laevis* | Inger, 1966 | N/A | N/A | N/A | N/A | N/A | N/A |
| Dicroglossidae | *Occidozyga lima* | Yang, 1991 | Yang, 1991 | Yang, 1991 | N/A | N/A | N/A | N/A |
| Dicroglossidae | *Occidozyga martensii* | Pope, 1931 | Yang, 1991 | N/A | N/A | N/A | N/A | N/A |
| Dicroglossidae | *Quasipaa boulengeri* | Yang, 1991 | Liu, 1950 | N/A | N/A | AmphibiaWeb | N/A | N/A |
| Dicroglossidae | *Quasipaa spinosa* | Chen, 1991 | Chen, 1991;  Huang, 1990;  Pope, 1931 | Chen, 1991;  Pope, 1931 | N/A | AmphibiaWeb | N/A | AmphibiaWeb |
| Dicroglossinae | *Feirana taihangnica* | Fei, 2009 | Fei, 2009 | N/A | N/A | N/A | N/A | Fei, 2009 |
| Dicroglossinae | *Limnonectes fujianensis* | Fei, 2009 | Fei, 2009 | Fei, 2009 | N/A | Fei, 2009 | N/A | Fei, 2009 |
| Dicroglossinae | *Nanorana ventripunctata* | Fei, 2009 | Fei, 2009 | Fei, 2009 | N/A | N/A | N/A | Fei, 2009 |
| Dicroglossinae | *Paa conaensis* | Fei, 2009 | Fei, 2009 | N/A | N/A | N/A | N/A | Fei, 2009 |
| Dicroglossinae | *Paa exilispinosa* | Fei, 2009 | Fei, 2009 | N/A | N/A | Fei, 2009 | N/A | N/A |
| Dicroglossinae | *Paa jiulongensis* | Fei, 2009 | N/A | N/A | N/A | Fei, 2009 | N/A | N/A |
| Dicroglossinae | *Paa liebigii* | Fei, 2009 | Fei, 2009 | N/A | N/A | N/A | N/A | Fei, 2009 |
| Dicroglossinae | *Paa liui* | Fei, 2009 | N/A | N/A | N/A | Fei, 2009 | N/A | N/A |
| Dicroglossinae | *Paa robertingeri* | Fei, 2009 | Fei, 2009 | N/A | N/A | Fei, 2009 | N/A | N/A |
| Dicroglossinae | *Paa shini* | Fei, 2009 | Fei, 2009 | Fei, 2009 | N/A | N/A | N/A | Fei, 2009 |
| Dicroglossinae | *Yerana yei* | Fei, 2009 | N/A | N/A | N/A | N/A | N/A | Fei, 2009 |
| Eleutherodactylidae | *Diasporus diastema* | Savage, 2002 | Savage, 2002  Ovaska, 2001 | Savage, 2002  Ovaska, 2001 | Ovaska, 2001 | Savage, 2002 | Ovaska, 2001 | Savage, 2002 |
| Eleutherodactylidae | *Eleutherodactylus coqui* | Townsend, 1994 | N/A | Townsend, 1994 | Duellman, 1994  Well, 1977 | Schwartz, 1991 | Duellman, 1994  Schwartz, 1991  Well, 1977 | N/A |
| Eleutherodactylidae | *Eleutherodactylus cundalli* | Diesel, 1995 | Diesel, 1995 | Schwartz, 1991  Diesel, 1995 | N/A | Schwartz, 1991 | Schwartz, 1991  Diesel, 1995 | N/A |
| Eleutherodactylidae | *Eleutherodactylus johnstonei* | Ortega, 2005 | Savage, 2002 | Savage, 2002 | Stebbins, 1995 | Savage, 2002 | Savage, 2002  Schwartz, 1991  Ortega, 2005 | N/A |
| Eleutherodactylidae | *Eleutherodactylus planirostris* | N/A | N/A | Schwartz, 1991 | N/A | Schwartz, 1991 | Schwartz, 1991 | N/A |
| Hemiphractidae | *Hemiphractus helioi* | N/A | N/A | N/A | N/A | AmphibiaWeb | AmphibiaWeb | N/A |
| Hemiphractidae | *Stefania evansi* | Duellman, 1984 | Summers, 2006 | Duellman, 1984 | N/A | Duellman, 1984 | Duellman, 1984 | N/A |
| Hemisotidae | *Hemisus marmoratum* | AmphibiaWeb | Wager, 1965 | Wager, 1965 | N/A | Wager, 1965 | Wager, 1965 | N/A |
| Hylidae | *Acris crepitans* | Wright, 1995;  Duellman, 2001 | Summers, 2006 | Degenhardt, 1996;  Smith, 1950 | Wagner, 1989 | N/A | N/A | N/A |
| Hylidae | *Acris gryllus* | Wright, 1995 | Stebbins, 1951 | Stebbins, 1951 | N/A | Wright, 1995 | N/A | Wright, 1995 |
| Hylidae | *Agalychnis callidryas* | Duellman, 2001 | Duellman, 2001;  Savage, 2002 | Duellman, 2001;  Savage, 2002 | Wells, 1977 | N/A | N/A | N/A |
| Hylidae | *Agalychnis litodryas* | Duellman, 2001 | N/A | N/A | N/A | Duellman, 2001 | N/A | AmphibiaWeb |
| Hylidae | *Agalychnis saltator* | Duellman, 2001 | Savage, 2002 | Savage, 2002 | Savage, 2002 | N/A | N/A | Savage, 2002 |
| Hylidae | *Agalychnis spurrelli* | Duellman, 2001 | N/A | N/A | N/A | Duellman, 2001 | N/A | Savage, 2002 |
| Hylidae | *Anotheca spinosa* | Duellman, 2001 | Jungfer, 1999;  Savage, 2002 | Savage, 2002;  Schiesari, 2003 | N/A | Savage, 2002 | Duellman, 1994 | N/A |
| Hylidae | *Aplastodiscus albofrenatus* | Lutz, 1973 | N/A | N/A | N/A | Lutz, 1973 | N/A | N/A |
| Hylidae | *Aplastodiscus perviridis* | N/A | Hadded, 2005 | Hadded, 2005 | N/A | Haddad, 2005 | Hadded, 2005 | N/A |
| Hylidae | *Bokermannohyla circumdata* | Lutz, 1973 | N/A | N/A | N/A | Lutz, 1973 | N/A | N/A |
| Hylidae | *Bokermannohyla martinsi* | Lutz, 1973 | N/A | N/A | N/A | Lutz, 1973 | N/A | N/A |
| Hylidae | *Bromeliohyla bromeliacia* | Lee, 2000 | N/A | Duellman, 2001 | N/A | Duellman, 2001 | Duellman, 1994 | N/A |
| Hylidae | *Charadrahyla taeniopus* | Duellman, 2001 | Summers, 2006 | N/A | N/A | Duellman, 2001 | N/A | N/A |
| Hylidae | *Cruziohyla calcarifer* | Duellman, 2001 | Savage, 2002 | Duellman, 2001;  Savage, 2002 | N/A | Savage, 2002 | N/A | Savage, 2002 |
| Hylidae | *Dendropsophus allenorum* | Rodriguez, 1994 | N/A | N/A | N/A | N/A | N/A | AmphibiaWeb |
| Hylidae | *Dendropsophus anceps* | Lutz, 1973 | Lutz, 1973 | N/A | N/A | Lutz, 1973 | N/A | N/A |
| Hylidae | *Dendropsophus aperomeus* | Duellman, 1982 | N/A | N/A | N/A | N/A | N/A | N/A |
| Hylidae | *Dendropsophus bifurcus* | Crump, 1974 | Crump, 1974 | Crump, 1974 | N/A | N/A | N/A | N/A |
| Hylidae | *Dendropsophus bipunctatus* | Lutz, 1973 | N/A | N/A | N/A | Lutz, 1973 | N/A | N/A |
| Hylidae | *Dendropsophus brevifrons* | Crump, 1974;  Duellman, 1974b | Crump, 1974 | Crump, 1974 | N/A | Duellman, 1974b | N/A | N/A |
| Hylidae | *Dendropsophus ebraccatus* | Duellman, 2001 | Duellman, 2001 | N/A | Savage, 2002 | N/A | N/A | Duellman, 2001 |
| Hylidae | *Dendropsophus elegans* | N/A | N/A | N/A | Bastos, 1996 | N/A | N/A | N/A |
| Hylidae | *Dendropsophus koechlini* | Rodriguez, 1994 | N/A | N/A | N/A | AmphibiaWeb | N/A | AmphibiaWeb |
| Hylidae | *Dendropsophus leucophyllatus* | Crump, 1974 | Crump, 1974 | Crump, 1974 | Savage, 2002 | N/A | N/A | Savage, 2002 |
| Hylidae | *Dendropsophus marmoratus* | Crump, 1974 | Crump, 1974 | Crump, 1974 | N/A | N/A | N/A | N/A |
| Hylidae | *Dendropsophus microcephalus* | Stebbins, 1959;  Duellman, 2001 | Stebbins, 1959 | Stebbins, 1959 | N/A | Stebbins, 1959 | N/A | N/A |
| Hylidae | *Dendropsophus minusculus* | Duellman, 1997 | N/A | N/A | N/A | Duellman, 1997 | N/A | N/A |
| Hylidae | *Dendropsophus minutus* | Crump, 1974 | Crump, 1974 | Crump, 1974 | N/A | Cei, 1980 | N/A | N/A |
| Hylidae | *Dendropsophus miyatai* | Bartlett, 2003 | Summers, 2006 | N/A | N/A | AmphibiaWeb | N/A | AmphibiaWeb |
| Hylidae | *Dendropsophus nanus* | Lutz, 1973 | Cei, 1980 | Cei, 1980 | N/A | N/A | N/A | N/A |
| Hylidae | *Dendropsophus parviceps* | Crump, 1974;  Duellman, 1974b | Crump, 1974 | Crump, 1974 | N/A | Duellman, 1974b | N/A | N/A |
| Hylidae | *Dendropsophus pelidna* | Duellman, 1989 | N/A | N/A | N/A | AmphibiaWeb | N/A | N/A |
| Hylidae | *Dendropsophus rhodopeplus* | Crump, 1974;  Duellman, 1996;  Rodriguez, 1994 | Crump, 1974 | Crump, 1974 | N/A | N/A | N/A | N/A |
| Hylidae | *Dendropsophus riveroi* | Duellman, 1996 | N/A | N/A | N/A | AmphibiaWeb | N/A | AmphibiaWeb |
| Hylidae | *Dendropsophus robertmertensi* | Duellman, 2001 | N/A | N/A | N/A | Duellman, 2001 | N/A | N/A |
| Hylidae | *Dendropsophus rubicundulus* | Lutz, 1973 | N/A | N/A | N/A | Lutz, 1973 | N/A | N/A |
| Hylidae | *Dendropsophus sanborni* | Lutz, 1973 | Cei, 1980 | Cei, 1980 | N/A | Lutz, 1973 | N/A | N/A |
| Hylidae | *Dendropsophus sarayacuensis* | Crump, 1974 | Crump, 1974 | Crump, 1974 | N/A | Zug, 2001 | N/A | N/A |
| Hylidae | *Dendropsophus sartori* | Duellman, 2001 | N/A | N/A | N/A | Duellman, 2001 | N/A | N/A |
| Hylidae | *Dendropsophus seniculus* | Lutz, 1973 | N/A | N/A | N/A | Lutz, 1973 | N/A | N/A |
| Hylidae | *Dendropsophus triangulum* | Crump, 1974;  Duellman, 1974b | Crump, 1974 | Crump, 1974 | N/A | AmphibiaWeb | N/A | AmphibiaWeb |
| Hylidae | *Duellmanohyla rufioculis* | Duellman, 2001 | Savage, 2002 | Savage, 2002 | N/A | Duellman, 2001 | N/A | N/A |
| Hylidae | *Ecnomiohyla miliaria* | Duellman, 2001 | N/A | N/A | Savage, 2002 | N/A | N/A | N/A |
| Hylidae | *Ecnomiohyla miotympanum* | Duellman, 2001 | Duellman, 2001 | Duellman, 2001 | N/A | Duellman, 2001 | N/A | N/A |
| Hylidae | *Exerodonta melanomma* | Duellman, 2001 | N/A | N/A | N/A | Duellman, 2001 | N/A | N/A |
| Hylidae | *Exerodonta sumichrasti* | Duellman, 2001 | Summers, 2006 | N/A | N/A | Duellman, 2001 | N/A | N/A |
| Hylidae | *Hyla andersonii* | Wright, 1995 | Wright, 1995 | Hulse, 2001 | N/A | Wright, 1995 | N/A | Hulse, 2001 |
| Hylidae | *Hyla annectans* | Yang, 1991;  Ye, 1993 | N/A | N/A | N/A | Xu, 2005 | N/A | N/A |
| Hylidae | *Hyla arborea* | Chen, 1991 | Kuzmin, 1999;  Okada, 1966 | Kuzmin, 1999 | N/A | Brzoska, 1982 | N/A | AmphibiaWeb |
| Hylidae | *Hyla arenicolor* | Wright, 1995 | Stebbins, 1951 | N/A | N/A | Degenhardt, 1996 | N/A | N/A |
| Hylidae | *Hyla avivoca* | Wright, 1995 | Summers, 2006 | AmphibiaWeb | Wright, 1995 | N/A | N/A | N/A |
| Hylidae | *Hyla chinensis* | Pope, 1931;  Ye, 1993;  Huang, 1990 | Huang, 1990 | Chen, 1991 | N/A | Chen, 1991 | N/A | N/A |
| Hylidae | *Hyla chrysoscelis* | Wright, 1995 | Hulse, 2001 | Hulse, 2001 | Fellers, 1979 | N/A | N/A | Hulse, 2001 |
| Hylidae | *Hyla cinerea* | Garton, 1975 | Wright, 1995 | Garton, 1975 | Garton, 1975 | N/A | N/A | Wright, 1995 |
| Hylidae | *Hyla euphorbiacea* | Duellman, 2001 | N/A | N/A | N/A | Duellman, 2001 | N/A | N/A |
| Hylidae | *Hyla eximia* | Duellman, 2001 | N/A | N/A | N/A | Degenhardt, 1996 | N/A | AmphibiaWeb |
| Hylidae | *Hyla femoralis* | Wright, 1995 | Wright, 1995 | N/A | N/A | Wright, 1995 | N/A | N/A |
| Hylidae | *Hyla gratiosa* | Wright, 1995 | Wright, 1995 | N/A | N/A | Murphy, 2003 |  | N/A |
| Hylidae | *Hyla japonica* | Okada, 1966 | Kuzmin, 1999 | Kuzmin, 1999 | N/A | Park, 1998 | N/A | N/A |
| Hylidae | *Hyla meridionalis* | N/A | Summers, 2006 | N/A | N/A | N/A | N/A | N/A |
| Hylidae | *Hyla plicata* | Duellman, 2001 | N/A | N/A | N/A | Duellman, 2001 | N/A | N/A |
| Hylidae | *Hyla savignyi* | N/A | Kuzmin, 1999 | Kuzmin, 1999 | N/A | Brzoska, 1982 | N/A | N/A |
| Hylidae | *Hyla squirella* | Wright, 1995 | Wright, 1995 | Wright, 1995 | Fellers, 1979 | N/A | N/A | N/A |
| Hylidae | *Hyla tsinlingensis* | Fei, 2009 | Fei, 2009 | N/A | N/A | Fei, 2009 | N/A | N/A |
| Hylidae | *Hyla versicolor* | Wright, 1995 | Summers, 2006 | Smith, 1950 | Fellers, 1979 | N/A | N/A | Wright, 1995 |
| Hylidae | *Hyla walkeri* | Duellman, 2001 | N/A | N/A | N/A | Duellman, 2001 | N/A | N/A |
| Hylidae | *Hyla wrightorum* | Wright, 1995 | Stebbins, 1951 | N/A | N/A | Wright, 1995 | N/A | N/A |
| Hylidae | *Hylomantis lemur* | Duellman, 1956 | Savage, 2002 | Savage, 2002 | Abrunhosa, 2004;  Savage, 2002 | N/A | N/A | Savage, 2002 |
| Hylidae | *Hyloscirtus armatus* | Duellman, 1997 | N/A | N/A | N/A | Duellman, 1997 | N/A | N/A |
| Hylidae | *Hyloscirtus colymba* | Duellman, 1972 | N/A | N/A | N/A | Savage, 2002 | N/A | N/A |
| Hylidae | *Hyloscirtus palmeri* | Savage, 2002 | N/A | N/A | N/A | Savage, 2002 | N/A | AmphibiaWeb |
| Hylidae | *Hyloscirtus phyllognathus* | Duellman, 1972 | Summers, 2006 | N/A | N/A | Duellman, 1972 | N/A | N/A |
| Hylidae | *Hyloscirtus simmonsi* | Duellman, 1989 | N/A | N/A | N/A | N/A | N/A | AmphibiaWeb |
| Hylidae | *Hypsiboas albomarginatus* | Hartmann, 2004 | N/A | N/A | N/A | Lutz, 1973 | N/A | N/A |
| Hylidae | *Hypsiboas albopunctatus* | Lutz, 1973 | N/A | N/A | N/A | Lutz, 1973 | N/A | N/A |
| Hylidae | *Hypsiboas andinus* | Duellman, 1997 | N/A | N/A | N/A | Duellman, 1997 | N/A | AmphibiaWeb |
| Hylidae | *Hypsiboas balzani* | Duellman, 1997 | N/A | N/A | N/A | Duellman, 1997 | N/A | N/A |
| Hylidae | *Hypsiboas bischoffi* | Lutz, 1973 | N/A | N/A | N/A | Lutz, 1973 | N/A | N/A |
| Hylidae | *Hypsiboas boans* | Crump, 1974;  Duellman, 1996;  Duellman, 2001 | Crump, 1974 | Crump, 1974 | N/A | Duellman, 2001 | Beck, 1998 | N/A |
| Hylidae | *Hypsiboas calcaratus* | Crump, 1974 | Crump, 1974 | Crump, 1974 | N/A | N/A | N/A | N/A |
| Hylidae | *Hypsiboas cinerascens* | Crump, 1974 | Crump, 1974 | Crump, 1974 | N/A | AmphibiaWeb | N/A | AmphibiaWeb |
| Hylidae | *Hypsiboas crepitans* | Duellman, 2001;  Stebbins, 1959 | Stebbins, 1959 | Stebbins, 1959 | N/A | Stebbins, 1959 | N/A | N/A |
| Hylidae | *Hypsiboas faber* | N/A | Lutz, 1973 | N/A | Martins, 1998 | N/A | Martins, 1998 | N/A |
| Hylidae | *Hypsiboas fasciatus* | Crump, 1974;  Duellman, 1996 | Crump, 1974 | Crump, 1974 | N/A | N/A | N/A | N/A |
| Hylidae | *Hypsiboas geographicus* | Crump, 1974 | Crump, 1974 | Crump, 1974 | N/A | Lutz, 1973 | N/A | N/A |
| Hylidae | *Hypsiboas guentheri* | Lutz, 1973 | N/A | N/A | N/A | Lutz, 1973 | N/A | N/A |
| Hylidae | *Hypsiboas heilprini* | Trueb, 1974 | N/A | N/A | N/A | Schwartz, 1991 | N/A | AmphibiaWeb |
| Hylidae | *Hypsiboas lanciformis* | Crump, 1974 | Crump, 1974 | Crump, 1974 | N/A | N/A | N/A | AmphibiaWeb |
| Hylidae | *Hypsiboas lemai* | Duellman, 1997 | Duellman, 1997 | Duellman, 1997 | N/A | Duellman, 1997 | N/A | N/A |
| Hylidae | *Hypsiboas marginatus* | N/A | N/A | N/A | N/A | N/A | N/A | AmphibiaWeb |
| Hylidae | *Hypsiboas marianitae* | Duellman, 1997 | N/A | N/A | N/A | Duellman, 1997 | N/A | N/A |
| Hylidae | *Hypsiboas pardalis* | Lutz, 1973 | N/A | N/A | Lutz, 1973 | N/A | Lutz, 1973 | N/A |
| Hylidae | *Hypsiboas polytaenius* | N/A | N/A | Lutz, 1973 | N/A | Lutz, 1973 | N/A | N/A |
| Hylidae | *Hypsiboas prasinus* | N/A | N/A | N/A | N/A | Lutz, 1973 | N/A | N/A |
| Hylidae | *Hypsiboas pulchellus* | Lutz, 1973 | Cei, 1980 | N/A | N/A | Lutz, 1973 | N/A | N/A |
| Hylidae | *Hypsiboas raniceps* | Lutz, 1973 | Cei, 1980;  Summers, 2006 | Cei, 1980 | N/A | Cei, 1980;  Lutz, 1973 | Summers, 2006 | N/A |
| Hylidae | *Hypsiboas rosenbergi* | Duellman, 2001 | Savage, 2002 | Savage, 2002 | Savage, 2002 | N/A | Savage, 2002 | N/A |
| Hylidae | *Hypsiboas rufitelus* | Duellman, 2001 | Savage, 2002 | N/A | N/A | Savage, 2002 | N/A | Savage, 2002 |
| Hylidae | *Hypsiboas semiguttatus* | Lutz, 1973 | N/A | N/A | N/A | Lutz, 1973 | N/A | N/A |
| Hylidae | *Hypsiboas sibleszi* | Duellman, 1997 | Duellman, 1997 | Duellman, 1997 | N/A | Duellman, 1997 | N/A | N/A |
| Hylidae | *Isthmohyla pseudopuma* | Duellman, 2001 | Savage, 2002 | Savage, 2002 | Crump, 1990 | N/A | N/A | Savage, 2002 |
| Hylidae | *Isthmohyla rivularis* | Duellman, 2001 | Summers, 2006 | Savage, 2002 | N/A | Savage, 2002 | N/A | N/A |
| Hylidae | *Isthmohyla tica* | Duellman, 2001 | Savage, 2002 | Savage, 2002 | N/A | Duellman, 2001 | N/A | N/A |
| Hylidae | *Isthmohyla zeteki* | Duellman, 2001 | N/A | Savage, 2002 | N/A | Savage, 2002 | N/A | Savage, 2002 |
| Hylidae | *Itapotihyla langsdorffii* | Lutz, 1973 | N/A | N/A | N/A | Lutz, 1973 | N/A | N/A |
| Hylidae | *Litoria arfakiana* | Menzies, 1976 | N/A | N/A | N/A | Menzies, 1976 | N/A | N/A |
| Hylidae | *Litoria aurea* | Moore, 1961 | Moore, 1961 | N/A | N/A | Moore, 1961 | N/A | N/A |
| Hylidae | *Litoria australis* | Parker, 1940 | Tyler, 1983 | Tyler, 1983 | N/A | Tyler, 1983 | N/A | Tyler, 1983 |
| Hylidae | *Litoria brevipes* | Parker, 1940 | Summers, 2006 | N/A | N/A | AmphibiaWeb | N/A | AmphibiaWeb |
| Hylidae | *Litoria caerulea* | Moore, 1961 | Tyler, 1983 | Moore, 1961;  Tyler, 1983 | N/A | Moore, 1961 | N/A | Tyler, 1983 |
| Hylidae | *Litoria cheesmani* | Menzies, 1976 | N/A | N/A | N/A | Zweifel, 1980 | N/A | N/A |
| Hylidae | *Litoria foricula* | Menzies, 1976 | N/A | N/A | N/A | Menzies, 1976 | N/A | N/A |
| Hylidae | *Litoria freycineti* | Moore, 1961 | Summers, 2006 | N/A | N/A | Moore, 1961 | N/A | N/A |
| Hylidae | *Litoria infrafrenata* | N/A | Animal Diversity Web | Animal Diversity Web | N/A | Menzies, 1976 | N/A | AmphibiaWeb |
| Hylidae | *Litoria kubori* | Menzies, 1976 | N/A | N/A | N/A | Menzies, 1976 | N/A | N/A |
| Hylidae | *Litoria meiriana* | N/A | N/A | Tyler, 1983;  AmphibiaWeb | N/A | Tyler, 1983 | N/A | Tyler, 1983 |
| Hylidae | *Litoria papua* | Menzies, 1976 | N/A | N/A | N/A | Menzies, 1976 | N/A | AmphibiaWeb |
| Hylidae | *Litoria peronii* | Tyler, 1978 | Summers, 2006 | N/A | Wells, 1977 | N/A | N/A | N/A |
| Hylidae | *Litoria rubella* | Tyler, 1978 | Tyler, 1983 | N/A | N/A | Hoser, 1989 | N/A | Tyler, 1978 |
| Hylidae | *Nyctimantis rugiceps* | Duellman, 2001 | N/A | N/A | N/A | Duellman, 1976 | AmphibiaWeb | N/A |
| Hylidae | Osteocephalus alboguttatus | Parker, 1940 | Summers, 2006 | N/A | N/A | Hoser, 1989 | N/A | N/A |
| Hylidae | *Osteocephalus buckleyi* | Trueb, 1971 | Crump, 1974;  Jungfer, 1999 | Jungfer, 1999 | N/A | AmphibiaWeb | N/A | N/A |
| Hylidae | *Osteocephalus leprieurii* | Trueb, 1971 | Crump, 1974 | Crump, 1974 | N/A | AmphibiaWeb | N/A | N/A |
| Hylidae | *Osteocephalus oophagus* | N/A | Jungfer, 1999 | Jungfer, 1999;  Schiesari, 2003 | Jungfer, 1999 | N/A | Jungfer, 1999 | AmphibiaWeb |
| Hylidae | *Osteocephalus taurinus* | Duellman, 1997;  Trueb, 1971 | Crump, 1974;  Summers, 2006 | Crump, 1974 | N/A | Duellman, 1997 | N/A | N/A |
| Hylidae | *Osteocephalus verruciger* | Trueb, 1971 | N/A | N/A | N/A | AmphibiaWeb | N/A | N/A |
| Hylidae | *Osteopilus brunneus* | Trueb, 1974 | Jungfer, 1999 | Schiesari, 2003 | N/A | Schwartz, 1991 | N/A | AmphibiaWeb |
| Hylidae | *Osteopilus crucialis* | Trueb, 1974 | N/A | N/A | N/A | Schwartz, 1991 | Duellman, 1994 | N/A |
| Hylidae | *Osteopilus dominicensis* | Trueb, 1974 | N/A | N/A | N/A | Schwartz, 1991 | N/A | N/A |
| Hylidae | *Osteopilus marianae* | Trueb, 1974 | N/A | N/A | N/A | Schwartz, 1991 | N/A | AmphibiaWeb |
| Hylidae | *Osteopilus pulchrilineatus* | Trueb, 1974 | N/A | N/A | N/A | Schwartz, 1991 | N/A | AmphibiaWeb |
| Hylidae | *Osteopilus septentrionalis* | Trueb, 1974;  Wright, 1995 | Savage, 2002 | Savage, 2002 | Salinas, 2006 | N/A | Salinas, 2006 | N/A |
| Hylidae | *Osteopilus vastus* | Trueb, 1974 | Schwartz, 1991 | N/A | N/A | Schwartz, 1991 | N/A | AmphibiaWeb |
| Hylidae | *Osteopilus wilderi* | Trueb, 1974 | N/A | N/A | N/A | N/A | N/A | AmphibiaWeb |
| Hylidae | *Pachymedusa dacnicolor* | Duellman, 2001 | Bagnara, 1986;  Wiewandt, 1971 | Bagnara, 1986;  Duellman, 2001 | Bagnara, 1986;  Duellman, 2001 | N/A | N/A | Bagnara, 1986;  Duellman, 2001 |
| Hylidae | *Phyllodytes luteolus* | N/A | Summers, 2006 | Schiesari, 2003 | N/A | N/A | N/A | AmphibiaWeb |
| Hylidae | *Phyllomedusa atelopoides* | Rodriguez, 1994 | AmphibiaWeb | AmphibiaWeb | N/A | AmphibiaWeb | AmphibiaWeb | N/A |
| Hylidae | *Phyllomedusa bicolor* | Rodriguez, 1994 | N/A | N/A | N/A | AmphibiaWeb | N/A | AmphibiaWeb |
| Hylidae | *Phyllomedusa hypochondrialis* | Duellman, 1997 | N/A | N/A | Abrunhosa, 2004 | N/A | N/A | AmphibiaWeb |
| Hylidae | *Phyllomedusa palliata* | Crump, 1974 | Crump, 1974 | Crump, 1974 | N/A | N/A | N/A | AmphibiaWeb |
| Hylidae | *Phyllomedusa tarsius* | Crump, 1974 | Crump, 1974 | Crump, 1974 | N/A | AmphibiaWeb | N/A | AmphibiaWeb |
| Hylidae | *Phyllomedusa tomopterna* | Crump, 1974 | Crump, 1974 | Crump, 1974 | N/A | N/A | N/A | AmphibiaWeb |
| Hylidae | *Phyllomedusa vaillantii* | Crump, 1974 | Crump, 1974 | Crump, 1974 | N/A | Zug, 2001 | N/A | N/A |
| Hylidae | *Plectrohyla arborescandens* | Caldwells, 1974;  Duellman, 2001 | Summers, 2006 | N/A | N/A | Duellman, 2001 | N/A | N/A |
| Hylidae | *Plectrohyla glandulosa* | Duellman, 2001 | N/A | N/A | N/A | AmphibiaWeb | N/A | N/A |
| Hylidae | *Plectrohyla guatemalensis* | Duellman, 1992 | Summers, 2006 | N/A | N/A | AmphibiaWeb | N/A | N/A |
| Hylidae | *Plectrohyla matudai* | Duellman, 2001 | N/A | N/A | N/A | AmphibiaWeb | N/A | AmphibiaWeb |
| Hylidae | *Plectrohyla pentheter* | Duellman, 2001 | N/A | N/A | N/A | AmphibiaWeb | N/A | N/A |
| Hylidae | *Pseudacris brachyphona* | Hulse, 2001 | Hulse, 2001;  Wright, 1995 | Hulse, 2001 | N/A | Wright, 1995 | N/A | Wright, 1995 |
| Hylidae | *Pseudacris brimleyi* | Wright, 1995 | N/A | AmphibiaWeb | N/A | Wright, 1995 | N/A | AmphibiaWeb |
| Hylidae | *Pseudacris cadaverina* | Duellman, 2001 | AmphibiaWeb | N/A | N/A | Duellman, 2001 | N/A | AmphibiaWeb |
| Hylidae | *Pseudacris clarkii* | Wright, 1995 | Wright, 1995 | Smith, 1950 | N/A | Duellman, 2001 | N/A | Wright, 1995 |
| Hylidae | *Pseudacris crucifer* | Wright, 1995 | Hulse, 2001;  Wright, 1995 | Hulse, 2001;  Wright, 1995 | Hulse, 2001 | N/A | N/A | N/A |
| Hylidae | *Pseudacris feriarum* | Wright, 1995 | Wright, 1995 | N/A | N/A | Wright, 1995 | N/A | N/A |
| Hylidae | *Pseudacris maculata* | Wright, 1995 | Wright, 1995 | N/A | N/A | Wright, 1995 | N/A | N/A |
| Hylidae | *Pseudacris nigrita* | Wright, 1995 | Wright, 1995 | Stebbins, 1951 | N/A | Dullman, 1994 | N/A | Stebbins, 1951 |
| Hylidae | *Pseudacris ocularis* | Wright, 1995 | Wright, 1995 | AmphibiaWeb | N/A | Wright, 1995 | N/A | N/A |
| Hylidae | *Pseudacris ornata* | Wright, 1995 | Wright, 1995 | AmphibiaWeb | N/A | Wright, 1995 | N/A | N/A |
| Hylidae | *Pseudacris regilla* | Duellman, 2001 | Stebbins, 1951 | Wright, 1995 | Wells, 1977 | N/A | N/A | Wright, 1995 |
| Hylidae | *Pseudacris streckeri* | Wright, 1995 | Wright, 1995 | Wright, 1995 | N/A | Wright, 1995 | N/A | N/A |
| Hylidae | *Pseudacris triseriata* | Wright, 1995 | Morrison, 2003 | Morrison, 2003 | N/A | Nussbaum, 1983 | N/A | Wright, 1995 |
| Hylidae | *Ptychohyla euthysanota* | Duellman, 2001 | N/A | N/A | N/A | Duellman, 2001 | N/A | N/A |
| Hylidae | *Ptychohyla hypomykter* | N/A | Summers, 2006 | N/A | N/A | N/A | N/A | N/A |
| Hylidae | *Ptychohyla leonhardschultzei* | Duellman, 2001 | N/A | N/A | N/A | Duellman, 2001 | N/A | N/A |
| Hylidae | *Ptychohyla spinipollex* | Duellman, 2001 | N/A | N/A | N/A | Duellman, 2001 | N/A | N/A |
| Hylidae | *Scarthyla goinorum* | Rodriguez, 1994 | N/A | N/A | N/A | N/A | N/A | AmphibiaWeb |
| Hylidae | *Scinax berthae* | Lutz, 1973 | Cei, 1980 | N/A | N/A | Cei, 1980 | N/A | N/A |
| Hylidae | *Scinax boulengeri* | Duellman, 2001 | Summers, 2006 | Savage, 2002 | N/A | Duellman, 2001;  Savage, 2002 | N/A | Savage, 2002 |
| Hylidae | *Scinax catharinae* | Lutz, 1973 | N/A | N/A | N/A | Lutz, 1973 | N/A | AmphibiaWeb |
| Hylidae | *Scinax elaeochraoa* | Duellman, 2001 | N/A | N/A | Savage, 2002 | N/A | N/A | Savage, 2002 |
| Hylidae | *Scinax fuscovarius* | Lutz, 1973;  Rodrigues, 2005 | Rodrigues, 2005 | Rodrigues, 2005 | N/A | Delariva, 1993;  Lutz, 1973 | N/A | N/A |
| Hylidae | *Scinax garbei* | Crump, 1974 | Crump, 1974 | Crump, 1974 | N/A | AmphibiaWeb | N/A | N/A |
| Hylidae | *Scinax nasicus* | Lutz, 1973 | N/A | N/A | N/A | Delariva, 1993 | N/A | N/A |
| Hylidae | *Scinax ruber* | Crump, 1974;  Duellman, 1996;  Duellman, 2001 | Crump, 1974 | Crump, 1974 | N/A | Duellman, 2001 | N/A | N/A |
| Hylidae | *Scinax squalirostris* | N/A | Cei, 1980 | N/A | N/A | Lutz, 1973 | N/A | Cei, 1980 |
| Hylidae | *Scinax staufferi* | Duellman, 2001 | N/A | N/A | N/A | Duellman, 2001;  Savage, 2002 | N/A | N/A |
| Hylidae | *Smilisca baudinii* | Duellman, 2001 | Duellman, 2001 | Duellman, 2001;  Savage, 2002 | N/A | Duellman, 2001 | N/A | Savage, 2002 |
| Hylidae | *Smilisca cyanosticta* | Duellman, 2001 | Duellman, 2001 | Duellman, 2001 | N/A | Duellman, 2001 | N/A | Duellman, 2001 |
| Hylidae | *Smilisca fodiens* | Duellman, 2001 | N/A | N/A | N/A | Duellman, 2001 | N/A | N/A |
| Hylidae | *Smilisca phaeota* | Savage, 2002 | N/A | Savage, 2002 | N/A | Savage, 2002 | N/A | N/A |
| Hylidae | *Smilisca puma* | Savage, 2002 | N/A | N/A | N/A | Savage, 2002 | N/A | N/A |
| Hylidae | *Sphaenorhynchus lacteus* | Rodriguez, 1994 | Summers, 2006 | N/A | N/A | AmphibiaWeb | N/A | N/A |
| Hylidae | *Tlalocohyla loquax* | Duellman, 2001 | N/A | Duellman, 2001 | N/A | Savage, 2002 | N/A | N/A |
| Hylidae | *Tlalocohyla picta* | Duellman, 2001 | N/A | N/A | N/A | Duellman, 2001 | N/A | N/A |
| Hylidae | *Tlalocohyla smithii* | Duellman, 2001 | N/A | N/A | N/A | Duellman, 2001 | N/A | N/A |
| Hylidae | *Trachycephalus coriaceus* | Rodriguez, 1994 | Crump, 1974 | Crump, 1974 | N/A | J. Bogart's personal communication | N/A | N/A |
| Hylidae | *Trachycephalus mesophaeus* | Lutz, 1973 | N/A | Lutz, 1973 | Lutz, 1973 | N/A | N/A | Lutz, 1973 |
| Hylidae | *Trachycephalus resinifictrix* | Lutz, 1973 | Schiesari, 2003 | Schiesari, 2003 | Schiesari, 2003 | N/A | Schiesari, 2003 | N/A |
| Hylidae | *Trachycephalus venulosus* | Duellman, 2001 | Rodrigues, 2005 | Rodrigues, 2005 | N/A | Savage, 2002 | N/A | N/A |
| Hylidae | *Triprion petasatus* | Duellman, 2001 | N/A | N/A | N/A | Duellman, 2001 | N/A | N/A |
| Hylidae | *Xenohyla truncata* | Lutz, 1973 | N/A | N/A | N/A | Lutz, 1973 | N/A | N/A |
| Hylodidae | *Hylodes phyllodes* | Hartmann, 2005 | N/A | N/A | Hartmann, 2005 | N/A | N/A | N/A |
| Hyperoliidae | *Afrixalus fornasini* | Schiotz, 1975 | Wager, 1965 | Wager, 1965 | N/A | Wager, 1965 | N/A | N/A |
| Hyperoliidae | *Alexteroon obstetricans* | N/A | N/A | N/A | N/A | AmphibiaWeb | Duellman, 1994 | N/A |
| Hyperoliidae | *Hyperolius lateralis* | Schiotz, 1975 | Vonesh, 2000 | Vonesh, 2000 | N/A | N/A | N/A | N/A |
| Hyperoliidae | *Hyperolius marmoratus* | Stewart, 1967 | Wager, 1965 | Wager, 1965 | Dyson, 1998 | N/A | N/A | N/A |
| Hyperoliidae | *Hyperolius nasutus* | Stewart, 1967  Wager, 1965 | Wager, 1965 | Wager, 1965 | Wager, 1965 | N/A | N/A | N/A |
| Hyperoliidae | *Hyperolius puncticulatus* | Clanning, 2001 | N/A | N/A | N/A | Wager, 1965 | N/A | N/A |
| Hyperoliidae | *Hyperolius tuberilinguis* | N/A | Wager, 1965 | Wager, 1965 | N/A | Wager, 1965 | N/A | N/A |
| Hyperoliidae | *Kassina senegalensis* | AmphibiaWeb | Wager, 1965 | Wager, 1965 | N/A | N/A | N/A | N/A |
| Leiopelmatidae | *Ascaphus truei* | Wright, 1995 | Nussbaum, 1983 | Stebbins, 1951 | N/A | Stebbins, 2003 | N/A | N/A |
| Leiopelmatidae | *Leiopelma archeyi* | AmphibiaWeb | Summers, 2006 | N/A | N/A | N/A | Duellman, 1994 | N/A |
| Leiopelmatidae | *Leiopelma hochstetteri* | AmphibiaWeb | Summers, 2006 | N/A | N/A | AmphibiaWeb | Duellman, 1994 | N/A |
| Leiuperidae | *Edalorhina perezi* | Duellman, 1996 | Crump, 1974 | Crump, 1974 | N/A | N/A | AmphibiaWeb | N/A |
| Leiuperidae | *Pleurodema brachyops* | Rivero, 1961 | N/A | N/A | N/A | Duellman, 1977 | N/A | AmphibiaWeb |
| Leiuperidae | *Pleurodema bufoninum* | Duellman, 1977 | N/A | N/A | N/A | Duellman, 1977 | N/A | Cei, 1980 |
| Leiuperidae | *Pleurodema thaul* | Duellman, 1977 | N/A | N/A | N/A | Cei, 1980 | N/A | Cei, 1980 |
| Leiuperidae | *Pseudopaludicola falcipes* | N/A | Cei, 1980 | N/A | N/A | Cei, 1980 | N/A | Cei, 1980 |
| Leptodactylidae | *Leptodactylus albilabris* | Heyer, 1978 | Schwartz, 1991 | Schwartz, 1991 | N/A | Schwartz, 1991 | N/A | N/A |
| Leptodactylidae | *Leptodactylus andreae* | Heyer, 1973 | Crump, 1974 | Crump, 1974 | N/A | N/A | N/A | N/A |
| Leptodactylidae | *Leptodactylus bufonius* | Reading, 2003  Heyer, 1978 | N/A | Cei, 1980 | N/A | Cei, 1980 | Reading, 2003 | Cei, 1980 |
| Leptodactylidae | *Leptodactylus fuscus* | Heyer, 1978 | Summers, 2006 | N/A | N/A | Martins, 1988 | Martins, 1998 | N/A |
| Leptodactylidae | *Leptodactylus gracilis* | Heyer, 1978 | N/A | N/A | N/A | Cei, 1980 | AmphibiaWeb | N/A |
| Leptodactylidae | *Leptodactylus hylaedactylus* | Heyer, 1973 | N/A | N/A | N/A | Heyer, 1973 | N/A | N/A |
| Leptodactylidae | *Leptodactylus labyrinthicus* | Silva, 2005 | Silva, 2005 | Silva, 2005 | Silva, 2005 | Cei, 1980 | Cei, 1980  Silva, 2005 | N/A |
| Leptodactylidae | *Leptodactylus leptodactyloides* | Heyer, 1994  Duellman, 1996 | N/A | N/A | N/A | Heyer, 1994 | N/A | N/A |
| Leptodactylidae | *Leptodactylus melanonotus* | Savage, 2002 | Savage, 2002 | Savage, 2002 | Duellman, 1994  Well, 1977 | Savage, 2002 | Savage, 2002 | N/A |
| Leptodactylidae | *Leptodactylus mystaceus* | Heyer, 1978  Duellman, 1997 | Crump, 1974 | Crump, 1974 | N/A | Cei, 1980 | N/A | N/A |
| Leptodactylidae | *Leptodactylus mystacinus* | Heyer, 1978 | Savage, 2002  Wright & Wright, 1949 | Savage, 2002  Wright & Wright, 1949 | N/A | Savage, 2002  Cei, 1980 | Savage, 2002 | N/A |
| Leptodactylidae | *Leptodactylus ocellatus* | Rivero, 1961 | Stebbins, 1951 | Stebbins, 1951 | N/A | Stebbins, 1951 | AmphibiaWeb | N/A |
| Leptodactylidae | *Leptodactylus pallidirostris* | Heyer, 1994 | N/A | N/A | N/A | Heyer, 1994 | N/A | N/A |
| Leptodactylidae | *Leptodactylus pentadactylus* | Savage, 2002  Rivero, 1961  Rodriguez, 1994  Duellman, 1997 | Savage, 2002 | Savage, 2002 | Well, 1977  Duellman, 1994 | Savage, 2002 | Savage, 2002 | N/A |
| Leptodactylidae | *Leptodactylus podicipinus* | Heyer, 1994 | N/A | N/A | N/A | Heyer, 1994 | N/A | N/A |
| Leptodactylidae | *Leptodactylus validus* | Heyer, 1994 | N/A | N/A | N/A | Heyer, 1994 | Downie, 1996 | N/A |
| Leptodactylidae | *Leptodactylus wagneri* | Heyer, 1994  Crump, 1974 | Crump, 1974 | Crump, 1974 | N/A | Schwartz, 1991 | N/A | N/A |
| Leptodactylidae | *Lithodytes lineatus* | Rodriguez, 1994 | Crump, 1974 | Crump, 1974 | N/A | N/A | AmphibiaWeb | N/A |
| Leptodactylidae | *Vanzolinius discodactylus* | Crump, 1974 | Crump, 1974 | Crump, 1974 | N/A | N/A | N/A | AmphibiaWeb |
| Limnodynastidae | *Adelotus brevis* | Katsikaros, 1997 | Summers, 2006 | N/A | Katsikaros, 1997 | N/A | Duellman, 1994 | N/A |
| Limnodynastidae | *Heleioporus australiacus* | Parker, 1940  Lee, 1967 | Moore, 1961 | Hoser, 1989 | N/A | Hoser, 1989  Moore, 1961  Lee, 1967 | Mains, 1965 | N/A |
| Limnodynastidae | *Lechriodus fletcheri* | Parker, 1940 | Moore, 1961 | AmphibiaWeb | N/A | Moore, 1961 | AmphibiaWeb | N/A |
| Limnodynastidae | *Limnodynastes convexiusculus* | Parker, 1940 | N/A | N/A | N/A | Hoser, 1989  Tyler, 1983 | Tyler, 1983 | N/A |
| Limnodynastidae | *Limnodynastes dumerilii* | Parker, 1940 | Summers, 2006 | AmphibiaWeb | Duellman, 1994 | N/A | Hoser, 1989 | N/A |
| Limnodynastidae | *Limnodynastes peronii* | Schauble, 2004 | Summers, 2006 | AmphibiaWeb | Wells, 1977 | N/A | AmphibiaWeb | N/A |
| Limnodynastidae | *Limnodynastes salmini* | Parker, 1940 | N/A | AmphibiaWeb | N/A | Frogs of the Greater Brisbane Region | N/A | N/A |
| Limnodynastidae | *Limnodynastes tasmaniensis* | Parker, 1940  Schauble, 2004 | N/A | Parker, 1940 | N/A | Hoser, 1989  Moore, 1961  Parker, 1940 | Parker, 1940 | N/A |
| Limnodynastidae | *Limnodynastes terraereginae* | Parker, 1940 | N/A | N/A | N/A | Hoser, 1989 | N/A | N/A |
| Limnodynastidae | *Neobatrachus pictus* | N/A | N/A | AmphibiaWeb | N/A | Moore, 1961 | N/A | N/A |
| Limnodynastidae | *Notaden melanoscaphus* | N/A | Tyler, 1983 | Tyler, 1983 | N/A | Tyler, 1983 | N/A | Tyler, 1983 |
| Limnodynastidae | *Philoria sphagnicolus* | Knowles, 2004 | N/A | Moore, 1961 | N/A | Moore, 1961 | AmphibiaWeb | N/A |
| Limnodynastidae | *Platyplectrum ornatum* | Parker, 1940 | Tyler, 1983 | Tyler, 1983 | N/A | Hoser, 1989 | Tyler, 1983 | N/A |
| Mantellidae | *Aglyptodactylus madagascariensis* | N/A | AmphibiaWeb | AmphibiaWeb | N/A | AmphibiaWeb | N/A | N/A |
| Mantellidae | *Boophis goudotii* | Blommers-  Schlosser, 1979 | Blommers-  Schlosser, 1979 | Blommers-  Schlosser, 1979 | N/A | Blommers-  Schlosser, 1979 | N/A | N/A |
| Mantellidae | *Boophis luteus* | Blommers-  Schlosser, 1979 | Blommers-  Schlosser, 1979 | Blommers-  Schlosser, 1979 | N/A | Blommers-  Schlosser, 1979 | N/A | N/A |
| Mantellidae | *Boophis rappiodes* | Blommers-  Schlosser, 1979 | Blommers-  Schlosser, 1979 | Blommers-  Schlosser, 1979 | N/A | N/A | N/A | N/A |
| Mantellidae | *Boophis tephraeomystax* | Blommers-  Schlosser, 1979 | Summers, 2006 | N/A | N/A | Blommers-  Schlosser, 1979 | N/A | N/A |
| Mantellidae | *Gephyromantis asper* | Vences, 2001 | N/A | N/A | N/A | Vence, 2001 | N/A | N/A |
| Mantellidae | *Gephyromantis horridus* | Vences, 2002 | N/A | N/A | N/A | Vences, 2002 | N/A | N/A |
| Mantellidae | *Gephyromantis luteus* | Vences, 2001 | N/A | N/A | N/A | Vence, 2001 | N/A | N/A |
| Mantellidae | *Gephyromantis malagasius* | Vences, 2002  Vences, 1998 | N/A | N/A | N/A | Vences, 2002 | N/A | N/A |
| Mantellidae | *Gephyromantis plicifer* | Vences, 2001 | N/A | N/A | N/A | Vence, 2001 | N/A | N/A |
| Mantellidae | *Gephyromantis striatus* | Vences, 2002 | N/A | N/A | N/A | Vences, 2002 | N/A | N/A |
| Mantellidae | *Mantella aurantiaca* | AmphibiaWeb | AmphibiaWeb | AmphibiaWeb | N/A | AmphibiaWeb | Glaw, 2000 | N/A |
| Mantellidae | *Mantella laevigata* | Vences, 1998 | N/A | Heying, 2004 | Heying, 2004  Heying, 2001 | N/A | Heying, 2004  Heying, 2001  Glaw, 2000 | N/A |
| Mantellidae | *Mantidactylus femoralis* | N/A | AmphibiaWeb | N/A | N/A | AmphibiaWeb | N/A | AmphibiaWeb |
| Megophryidae | *Leptobrachium ailaonicum* | Ye, 1993 | Fei, 1999 | Ye, 1993 | N/A | Fei, 1999 | N/A | Ye, 1993 |
| Megophryidae | *Leptobrachium banae* | Lathrop, 1998 | N/A | N/A | N/A | Lathrop, 1998 | N/A | N/A |
| Megophryidae | *Leptobrachium boringii* | Ye, 1993 | Ye, 1993 | Ye, 1993 | N/A | Fei, 1999 | Ye, 1993 | N/A |
| Megophryidae | *Leptobrachium chapaense* | Lathrop, 1998 | N/A | N/A | N/A | Fei, 1999 | N/A | N/A |
| Megophryidae | *Leptobrachium hasseltii* | Inger, 1966 | N/A | N/A | N/A | Dring, 1979 | N/A | N/A |
| Megophryidae | *Leptobrachium leishanense* | Ye, 1993 | Fei, 1999 | Ye, 1993 | N/A | Ye, 1993 | N/A | Ye, 1993 |
| Megophryidae | *Leptobrachium liui* | Ye, 1993 | Ye, 1993 | Ye, 1993 | N/A | Fei, 1999 | Huang, 1990 | N/A |
| Megophryidae | *Leptobrachium montanum* | Ohler, 2004 | N/A | N/A | N/A | N/A | N/A | N/A |
| Megophryidae | *Leptobrachium mouhoti* | Stuart, 2006 | N/A | N/A | N/A | Fei, 1999 | N/A | N/A |
| Megophryidae | *Leptobrachium promustache* | Rao, 2006 | N/A | N/A | N/A | Rao, 2006 | N/A | N/A |
| Megophryidae | *Leptolalax liui* | Fei, 1999 | N/A | N/A | N/A | Fei, 1999 | N/A | N/A |
| Megophryidae | *Leptolalax pelodytoides* | Huang, 1990 | Pope, 1931 | Pope, 1931 | N/A | Huang, 1990 | N/A | N/A |
| Megophryidae | *Oreolalax jingdongensis* | N/A | Fei, 1999 | Fei, 1999 | N/A | Fei, 1999 | N/A | N/A |
| Megophryidae | *Oreolalax liangbeiensis* | Ye, 1993 | Ye, 1993 | Fei, 1999 | N/A | Fei, 1999 | N/A | Ye, 1993 |
| Megophryidae | *Oreolalax multipunctatus* | N/A | Fei, 1999 | N/A | N/A | Fei, 1999 | Fei, 1999 | N/A |
| Megophryidae | *Oreolalax pingii* | Ye, 1993 | Ye, 1993 | Fei, 1999;  Ye, 1993 | N/A | Ye, 1993 | N/A | N/A |
| Megophryidae | *Oreolalax popei* | Ye, 1993 | Ye, 1993 | Ye, 1993 | N/A | Fei, 1999 | N/A | N/A |
| Megophryidae | *Oreolalax rugosus* | Ye, 1993 | Ye, 1993 | N/A | N/A | Ye, 1993 | N/A | N/A |
| Megophryidae | *Oreolalax schmidti* | Fei, 1999 | Fei, 1999 | Liu, 1950 | N/A | Fei, 1999 | N/A | N/A |
| Megophryidae | *Scutiger boulengeri* | Hu, 1987;  Ye, 1993 | Ye, 1993 | Ye, 1993 | N/A | Fei, 1999 | N/A | N/A |
| Megophryidae | *Scutiger glandulatus* | Yang, 1991 | Fei, 1999 | N/A | N/A | Fei, 1999 | N/A | Liu, 1950 |
| Megophryidae | *Scutiger mammatus* | Ye, 1993 | Ye, 1993 | Ye, 1993 | N/A | Fei, 1999 | N/A | N/A |
| Megophryidae | *Scutiger muliensis* | Fei, 1999 | N/A | Fei, 1999 | N/A | Fei, 1999 | N/A | N/A |
| Megophryidae | *Scutiger tuberculatus* | Ye, 1993 | Ye, 1993 | N/A | N/A | Ye, 1993 | N/A | Ye, 1993 |
| Megophyidae | *Brachytarsophrys feae* | Fei, 2009 | Fei, 2009 | Fei, 2009 | N/A | Fei, 2009 | N/A | Fei, 2009 |
| Megophyidae | *Ophryophryne microstoma* | Ye, 1993 | Ye, 1993 | N/A | N/A | N/A | N/A | N/A |
| Megophyidae | *Oreolalax chuanbeiensis* | Fei, 2009 | Fei, 2009 | Fei, 2009 | N/A | Fei, 2009 | Fei, 2009 | N/A |
| Megophyidae | *Oreolalax lichuanensis* | Fei, 2009 | Fei, 2009 | Fei, 2009 | N/A | Fei, 2009 | N/A | Fei, 2009 |
| Megophyidae | *Oreolalax major* | Fei, 2009 | Fei, 2009 | N/A | N/A | Fei, 2009 | N/A | Fei, 2009 |
| Megophyidae | *Oreolalax omeimontis* | Fei, 2009 | Fei, 2009 | Fei, 2009 | N/A | N/A | N/A | N/A |
| Megophyidae | *Oreolalax xiangchengensis* | Fei, 2009 | Fei, 2009 | N/A | N/A | N/A | N/A | N/A |
| Megophyidae | *Scutiger chintingensis* | Fei, 2009 | Fei, 2009 | Fei, 2009 | N/A | Fei, 2009 | N/A | Fei, 2009 |
| Megophyidae | *Xenophrys minor* | Fei, 2009 | N/A | N/A | N/A | Fei, 2009 | N/A | N/A |
| Megophyidae | *Xenophrys omeimontis* | Fei, 2009 | Fei, 2009 | Fei, 2009 | N/A | Fei, 2009 | N/A | Fei, 2009 |
| Megophyidae | *Xenophrys shapingensis* | Fei, 2009 | Fei, 2009 | N/A | N/A | Fei, 2009 | N/A | N/A |
| Megophyidae | *Xenophrys spinata* | Fei, 2009 | Fei, 2009 | N/A | N/A | Fei, 2009 | N/A | N/A |
| Microhylidae | *Chaperina fusca* | Inger, 1966 | N/A | N/A | N/A | Inger, 1966 | N/A | N/A |
| Microhylidae | *Gastrophryne olivacea* | N/A | Wright, 1995 | Degenhardt, 1996;  Smith, 1950 | N/A | Wells, 1977 | N/A | AmphibiaWeb |
| Microhylidae | *Hylophorbus rufescens* | Zweifel, 1956 | Bickford, 2004 | Bickford, 2004 | Bickford, 2004 | N/A | Bickford, 2004 | N/A |
| Microhylidae | *Kalophrynus pleurostigma* | Inger, 1966 | Pope, 1931 | Pope, 1931 | N/A | N/A | N/A | Inger, 1966 |
| Microhylidae | *Kaloula pulchra* | Inger, 1966 | N/A | N/A | N/A | N/A | N/A | N/A |
| Microhylidae | *Metaphrynella sundana* | Inger, 1966 | N/A | N/A | Lardner, 2004 | N/A | N/A | N/A |
| Microhylidae | *Microhyla butleri* | Fei, 2009 | Fei, 2009 | N/A | N/A | Fei, 2009 | N/A | N/A |
| Microhylidae | *Microhyla heymonsi* | Yang, 1991 | Huang, 1990 | Chen, 1991;  Huang, 1990 | N/A | Dring, 1979 | N/A | N/A |
| Microhylidae | *Microhyla ornata* | Yang, 1991  Ye, 1993  Pope, 1931 | Chen, 1991  Huang, 1990 | Chen, 1991  Huang, 1990 | N/A | Ye, 1993  Chen, 1991 | N/A | N/A |
| Microhylidae | *Microhyla pulchra* | Fei, 2009 | Fei, 2009 | Fei, 2009 | N/A | Fei, 2009 | N/A | Fei, 2009 |
| Microhylidae | *Phrynomantis bifasciatus* | N/A | Wager, 1965 | Wager, 1965 | N/A | Wager, 1965 | Beck, 1998 | N/A |
| Microphylidae | *Aphantophryne pansa* | N/A | N/A | Zweifel, 1956 | N/A | N/A | N/A | N/A |
| Myobatrachidae | *Crinia nimbus* | N/A | Mitchell, 2002 | AmphibiaWeb | N/A | AmphibiaWeb | Mitchell, 2002 | N/A |
| Myobatrachidae | *Crinia signifera* | Parker, 1940 | Moore, 1961 | Moore, 1961 | N/A | Hoser, 1989 | N/A | AmphibiaWeb |
| Myobatrachidae | *Geocrinia victoriana* | N/A | Morrison, 2003 | AmphibiaWeb | N/A | Parker, 1940 | N/A | AmphibiaWeb |
| Myobatrachidae | *Metacrinia nichollsi* | Parker, 1940 | AmphibiaWeb | N/A | N/A | N/A | N/A | N/A |
| Myobatrachidae | *Mixophyes fasciolatus* | Parker, 1940  Morrison, 2003 | Morrison, 2003 | Morrison, 2003 | N/A | Hoser, 1989 | N/A | N/A |
| Myobatrachidae | *Myobatrachus gouldii* | Parker, 1940 | Summers, 2006 | AmphibiaWeb | N/A | Main, 1965;  AmphibiaWeb | N/A | AmphibiaWeb |
| Myobatrachidae | *Paracrinia haswelli* | Parker, 1940 | N/A | N/A | N/A | Moore, 1961 | N/A | AmphibiaWeb |
| Myobatrachidae | *Pseudophryne bibronii* | Parker, 1940 | Moore, 1961 | Moore, 1961;  Parker, 1940;  Tyler, 1978 | Duellman, 1994 | N/A | Duellman, 1994 | N/A |
| Myobatrachidae | *Rheobatrachus silus* | N/A | Summers, 2006 | AmphibiaWeb | N/A | AmphibiaWeb | Deullman, 1994 | N/A |
| Myobatrachidae | *Spicospina flammocaerulea* | N/A | N/A | AmphibiaWeb | N/A | Smith, 2003 | N/A | AmphibiaWeb |
| Myobatrachidae | *Uperoleia laevigata* | N/A | N/A | N/A | N/A | Hoser, 1989 | N/A | AmphibiaWeb |
| Nyctibatrachidae | *Lankanectes corrugatus* | N/A | N/A | N/A | N/A | Alcala, 1986 | N/A | Alcala, 1986 |
| Pelobatidae | *Pelobates fuscus* | N/A | Kuzmin, 1999 | Kuzmin, 1999 | Kuzmin, 1999 | N/A | N/A | AmphibiaWeb |
| Pelobatidae | *Pelobates syriacus* | Kuzmin, 1999 | Kuzmin, 1999 | Kuzmin, 1999 | N/A | Kuzmin, 1999 | N/A | N/A |
| Pelodytidae | *Pelodytes caucasicus* | Kuzmin, 1999 | N/A | N/A | Kuzmin, 1999 | N/A | N/A | N/A |
| Petropedetidae | *Conraua goliath* | Sabater-Pi, 1985 | Sabater-Pi, 1985 | Sabater-Pi, 1985 | N/A | N/A | N/A | Sabater-Pi, 1985 |
| Petropedetidae | *Petropedetes yakusini* | Channing, 2002 | Channing, 2002 | Channing, 2002 | N/A | Channing, 2002 | Beck, 1998 | N/A |
| Phrynobatrachidae | *Phrynobatrachus mababiensis* | Stewart, 1967 | Wager, 1965 | Stewart, 1967;  Wager, 1965 | N/A | N/A | N/A | N/A |
| Phrynobatrachidae | *Phrynobatrachus natalensis* | Stewart, 1967 | Wager, 1965 | Wager, 1965 | N/A | Wager, 1965 | N/A | AmphibiaWeb |
| Pipidae | *Hymenochirus boettgeri* | Rabb, 1963 | Rabb, 1962 | Rabb, 1962 | Rabb, 1962 | N/A | N/A | Rabb, 1962 |
| Pipidae | *Pipa carvalhoi* | Trueb, 1986 | Weygoldt, 1976 | Weygoldt, 1976 | Duellman, 1994 | N/A | Duellman, 1994 | N/A |
| Pipidae | *Pipa parva* | Trueb, 1986 | N/A | N/A | Duellman, 1994 | N/A | Duellman, 1994 | N/A |
| Pipidae | *Pipa pipa* | Trueb, 1986 | Summers, 2006 | Bartlett, 2003 | Rabb, 1963 | N/A | Duellman, 1994 | N/A |
| Pipidae | *Xenopus laevis* | N/A | Wager, 1965 | AmphibiaWeb | Rabb, 1963 | N/A | N/A | Wager, 1965 |
| Pipidae | *Xenopus muelleri* | Stewart, 1967 | N/A | N/A | N/A | Vager, 1965 | N/A | N/A |
| Ptychadenidae | *Ptychadena anchietae* | N/A | N/A | N/A | N/A | Wager, 1965 | N/A | N/A |
| Ptychadenidae | *Ptychadena mascareniensis* | Channing, 2001 | Summers, 2006 | N/A | N/A | Wager, 1965 | N/A | N/A |
| Pyxicephalidae | *Afrana angolensis* | N/A | Wager, 1965 | N/A | N/A | Wager, 1965 | N/A | N/A |
| Pyxicephalidae | *Afrana fuscigula* | Stewart, 1967 | Wager, 1965 | Wager, 1965 | N/A | Wager, 1965 | N/A | N/A |
| Pyxicephalidae | *Anhydrophryne rattrayi* | N/A | N/A | AmphibiaWeb | N/A | Wager, 1965 | N/A | AmphibiaWeb |
| Pyxicephalidae | *Natalobatrachus bonebergi* | N/A | Wager, 1965 | Wager, 1965 | N/A | Wager, 1965 | N/A | AmphibiaWeb |
| Pyxicephalidae | *Pyxicephalus adspersus* | Cook, 2001 | Wager, 1965 | Wager, 1965 | Wager, 1965 | Wager, 1965  Cook, 2001 | Cook, 2001 | N/A |
| Pyxicephalidae | *Strongylopus grayii* | Channing, 2001 | N/A | N/A | N/A | Wager, 1965 | N/A | N/A |
| Pyxicephalidae | *Tomopterna delalandii* | N/A | Summers, 2006 | N/A | N/A | AmphibiaWeb | N/A | N/A |
| Ranidae | *Amolops bellulus* | Fei, 2009 | N/A | N/A | N/A | Fei, 2009 | N/A | N/A |
| Ranidae | *Amolops chunganensis* | Ye, 1993 | Ye, 1993 | Inger, 1968 | Liu, 1950 | N/A | N/A | Liu, 1950 |
| Ranidae | *Amolops cremnobatus* | Inger, 1998 | Inger, 1998 | N/A | N/A | N/A | N/A | N/A |
| Ranidae | *Amolops daiyunensis* | Fei, 2009 | Fei, 2009 | N/A | N/A | N/A | N/A | Fei, 2009 |
| Ranidae | *Amolops granulosus* | Fei, 2009 | N/A | N/A | N/A | Fei, 2009 | N/A | Fei, 2009 |
| Ranidae | *Amolops hainanensis* | Fei, 2009 | Fei, 2009 | N/A | N/A | N/A | N/A | Fei, 2009 |
| Ranidae | *Amolops hongkongensis* | Fei, 2009 | Fei, 2009 | N/A | N/A | N/A | N/A | N/A |
| Ranidae | *Amolops lifanensis* | Fei, 2009 | Fei, 2009 | N/A | N/A | N/A | N/A | Fei, 2009 |
| Ranidae | *Amolops loloensis* | Fei, 2009 | Fei, 2009 | Fei, 2009 | N/A | N/A | N/A | Fei, 2009 |
| Ranidae | *Amolops mantzorum* | Yang, 1991 | Ye, 1993 | N/A | N/A | AmphibiaWeb | N/A | N/A |
| Ranidae | *Amolops ricketti* | Pope, 1931 | Ye, 1993 | N/A | N/A | Ye, 1993 | N/A | N/A |
| Ranidae | *Amolops torrentis* | Fei, 2009 | Fei, 2009 | N/A | N/A | Fei, 2009 | N/A | N/A |
| Ranidae | *Amolops viridimaculatus* | Yang, 1991 | N/A | N/A | N/A | AmphibiaWeb | N/A | N/A |
| Ranidae | *Amolops wuyiensis* | Chen, 1991 | Huang, 1990 | Huang, 1990 | N/A | AmphibiaWeb | N/A | N/A |
| Ranidae | *Babina holsti* | Fei, 2009 | Fei, 2009 | N/A | N/A | N/A | N/A | Fei, 2009 |
| Ranidae | *Babina okinavana* | Inger, 1947 | N/A | AmphibiaWeb | N/A | AmphibiaWeb | N/A | N/A |
| Ranidae | *Babina pleuraden* | Yang, 1991 | Ye, 1993 | Yang, 1991 | N/A | Yang, 1991 | N/A | N/A |
| Ranidae | *Fejervarya nicobariensis* | Inger, 1966 | N/A | N/A | N/A | AmphibiaWeb | N/A | N/A |
| Ranidae | *Glandirana emeljanovi* | Ye, 1993 | Ye, 1993 | Ye, 1993 | N/A | Ye, 1993 | N/A | N/A |
| Ranidae | *Glandirana minima* | Ye, 1993 | N/A | Ye, 1993 | N/A | Ye, 1993 | N/A | Ye, 1993 |
| Ranidae | *Glandirana rugosa* | Fei, 2009 | Fei, 2009 | Fei, 2009 | N/A | N/A | N/A | Fei, 2009 |
| Ranidae | *Glandirana tientaiensis* | Chen, 1991 | Huang, 1990 | Chen, 1991 | N/A | Huang, 1990 | N/A | Huang, 1990 |
| Ranidae | *Hydrophylax galamensis* | Stewart, 1967 | AmphibiaWeb | AmphibiaWeb | N/A | AmphibiaWeb | N/A | AmphibiaWeb |
| Ranidae | *Hylarana baramica* | Fei, 2009 | N/A | N/A | N/A | Fei, 2009 | N/A | N/A |
| Ranidae | *Hylarana chalconota* | Bain, 2003 | N/A | N/A | N/A | Marquez, 2006 | N/A | N/A |
| Ranidae | *Hylarana daemeli* | Wells, 1977 | N/A | AmphibiaWeb | N/A | N/A | N/A | AmphibiaWeb |
| Ranidae | *Hylarana erythraea* | Inger, 1966 | N/A | N/A | N/A | Inger, 1966 | N/A | N/A |
| Ranidae | *Hylarana guentheri* | Pope, 1931 | Huang, 1990;  Pope, 1931 | Huang, 1990;  Pope, 1931 | N/A | Xu, 2005 | N/A | N/A |
| Ranidae | *Hylarana latouchii* | Fei, 2009 | Fei, 2009 | Fei, 2009 | N/A | Fei, 2009 | N/A | Fei, 2009 |
| Ranidae | *Hylarana macrodactyla* | Fei, 2009 | Fei, 2009 | Fei, 2009 | N/A | N/A | N/A | N/A |
| Ranidae | *Hylarana nigrovittata* | Yang, 1991 | Yang, 1991 | Yang, 1991 | N/A | N/A | N/A | N/A |
| Ranidae | *Hylarana signata* | Inger, 1966 | Alcala, 1986 | N/A | N/A | Dring, 1979 | N/A | AmphibiaWeb |
| Ranidae | *Hylarana taipehensis* | Yang, 1991 | Yang, 1991;  Ye, 1993 | N/A | N/A | Ye, 1993 | N/A | N/A |
| Ranidae | *Hylarana temporalis* | N/A | Summers, 2006 | N/A | N/A | AmphibiaWeb | N/A | N/A |
| Ranidae | *Lithobates areolatus* | Wright, 1995 | Wright, 1995 | Wright, 1995 | Wells, 1977 | N/A | N/A | N/A |
| Ranidae | Lithobates berlandieri | N/A | N/A | AmphibiaWeb | N/A | Degenhardt, 1996 | N/A | AmphibiaWeb |
| Ranidae | *Lithobates blairi* | N/A | N/A | AmphibiaWeb | N/A | Frost, 1977 | N/A | AmphibiaWeb |
| Ranidae | Lithobates capito | Wright, 1995 | Wright, 1995 | Goin, 1940 | N/A | Wright, 1995 | N/A | N/A |
| Ranidae | *Lithobates catesbeianus* | Hulse, 2001 | Wright, 1995;  Ye, 1993 | Degenhardt, 1996;  Hulse, 2001;  Smith, 1950;  Ye, 1993 | Wells, 1977 | Degenhardt, 1996 | N/A | Ye, 1993 |
| Ranidae | *Lithobates chiricahuensis* | N/A | N/A | AmphibiaWeb | N/A | Degenhardt, 1996 | N/A | AmphibiaWeb |
| Ranidae | *Lithobates clamitans* | Wright, 1995 | Wright, 1995 | Nussbaum, 1983;  Wright, 1995 | Wells, 1977 | N/A | N/A | Stebbins, 1951 |
| Ranidae | *Lithobates forreri* | Savage, 2002 | N/A | N/A | N/A | Savage, 2002 | N/A | N/A |
| Ranidae | *Lithobates grylio* | Wright, 1995 | N/A | AmphibiaWeb | N/A | Schwartz, 1991 | N/A | AmphibiaWeb |
| Ranidae | *Lithobates heckscheri* | Wright, 1995 | AmphibiaWeb | AmphibiaWeb | N/A | AmphibiaWeb | N/A | AmphibiaWeb |
| Ranidae | *Lithobates onca* | Wright, 1995 | N/A | AmphibiaWeb | N/A | AmphibiaWeb | N/A | AmphibiaWeb |
| Ranidae | *Lithobates palmipes* | Crump, 1974 | Crump, 1974 | Crump, 1974 | N/A | Zug, 2001 | N/A | N/A |
| Ranidae | *Lithobates palustris* | Wright, 1995 | Wright, 1995 | Wright, 1995 | Wells, 1977 | N/A | N/A | Smith, 1950 |
| Ranidae | *Lithobates pipiens* | Hulse, 2001 | Stebbins, 1951 | Degenhardt, 1996 | Wells, 1977 | N/A | N/A | N/A |
| Ranidae | *Lithobates septentrionalis* | Wright, 1995 | Hulse, 2001 | Hulse, 2001 | N/A | Wright, 1995 | N/A | N/A |
| Ranidae | *Lithobates sevosus* | Wright, 1995 | AmphibiaWeb | AmphibiaWeb | N/A | AmphibiaWeb | N/A | N/A |
| Ranidae | *Lithobates sphenocephalus* | Hulse, 2001 | Wright, 1995 | Hulse, 2001 | N/A | Schwartz, 1991 | N/A | N/A |
| Ranidae | *Lithobates sylvaticus* | Hulse, 2001 | Morrison, 2003;  Stebbins, 1951 | Morrison, 2003 | Wells, 1977 | N/A | N/A | Hulse, 2001 |
| Ranidae | *Lithobates tarahumarae* | N/A | Stebbins, 1951 | Stebbins, 1951 | N/A | Stebbins, 2003 | N/A | N/A |
| Ranidae | *Lithobates vaillanti* | N/A | N/A | N/A | N/A | Savage, 2002 | N/A | Savage, 2002 |
| Ranidae | *Lithobates vibicarius* | Savage, 2002 | Savage, 2002 | N/A | N/A | Savage, 2002 | N/A | N/A |
| Ranidae | *Lithobates virgatipes* | Wright, 1995 | Wright, 1995 | Wright, 1995 | Given, 1988 | N/A | N/A | N/A |
| Ranidae | *Lithobates warszewitschii* | Savage, 2002 | N/A | N/A | N/A | Savage, 2002 | N/A | N/A |
| Ranidae | *Meristogenys jerboa* | Inger, 1966 | Inger, 1968 | Inger, 1968 | N/A | AmphibiaWeb | N/A | N/A |
| Ranidae | *Meristogenys kinabaluensis* | Inger, 1966 | Inger, 1966 | N/A | N/A | AmphibiaWeb | N/A | N/A |
| Ranidae | *Odorrana andersonii* | Pope, 1931 | N/A | N/A | N/A | Liu, 1950 | N/A | N/A |
| Ranidae | *Odorrana banaorum* | Fei, 2009 | Fei, 2009 | N/A | N/A | N/A | N/A | Fei, 2009 |
| Ranidae | *Odorrana chapaensis* | Yang, 1991 | N/A | N/A | N/A | AmphibiaWeb | N/A | N/A |
| Ranidae | *Odorrana chloronota* | Fei, 2009 | Fei, 2009 | N/A | N/A | N/A | N/A | Fei, 2009 |
| Ranidae | *Odorrana grahami* | Yang, 1991 | Ye, 1993 | Yang, 1991 | N/A | Ye, 1993 | N/A | Ye, 1993 |
| Ranidae | *Odorrana hosii* | Fei, 2009 | Fei, 2009 | Fei, 2009 | N/A | N/A | N/A | Fei, 2009 |
| Ranidae | *Odorrana jingdongensis* | Fei, 2009 | Fei, 2009 | N/A | N/A | N/A | N/A | N/A |
| Ranidae | *Odorrana junlianensis* | Fei, 2009 | Fei, 2009 | N/A | N/A | N/A | N/A | N/A |
| Ranidae | *Odorrana livida* | Chen, 1991 | Chen, 1991;  Huang, 1990;  Yang, 1991 | Chen, 1991;  Huang, 1990 | N/A | Yang, 1991 | N/A | N/A |
| Ranidae | *Odorrana margaretae* | Bain, 2003 | N/A | N/A | N/A | AmphibiaWeb | N/A | N/A |
| Ranidae | *Odorrana morafkai* | Fei, 2009 | Fei, 2009 | N/A | N/A | N/A | N/A | Fei, 2009 |
| Ranidae | *Odorrana schmackeri* | Ye, 1993 | Chen, 1991 | Chen, 1991;  Huang, 1990 | N/A | Chen, 1991 | N/A | N/A |
| Ranidae | *Odorrana swinhoana* | Fei, 2009 | Fei, 2009 | Fei, 2009 | N/A | Fei, 2009 | N/A | Fei, 2009 |
| Ranidae | *Odorrana tiannanensis* | Fei, 2009 | Fei, 2009 | N/A | N/A | N/A | N/A | Fei, 2009 |
| Ranidae | *Odorrana tormota* | Fei, 2009 | Fei, 2009 | Fei, 2009 | N/A | Fei, 2009 | N/A | Fei, 2009 |
| Ranidae | *Odorrana versabilis* | Chen, 1991 | Chen, 1991 | Chen, 1991 | N/A | AmphibiaWeb | N/A | N/A |
| Ranidae | *Pelophylax esculenta* | Fei, 2009 | Fei, 2009 | Fei, 2009 | N/A | Fei, 2009 | N/A | Fei, 2009 |
| Ranidae | *Pelophylax fukienensis* | Fei, 2009 | Fei, 2009 | Fei, 2009 | N/A | Fei, 2009 | N/A | Fei, 2009 |
| Ranidae | *Pelophylax lessonae* | N/A | Kuzmin, 1999 | Beebee, 2000;  Kuzmin, 1999 | Beebee, 2000 | N/A | N/A | N/A |
| Ranidae | *Pelophylax lessonae* | N/A | N/A | Fei, 2009 | Fei, 2009 | N/A | N/A | Fei, 2009 |
| Ranidae | *Pelophylax nigromaculatus* | Chen, 1991 | Chen, 1991;  Kuzmin, 1999;  Liu, 1950 | Chen, 1991;  Huang, 1990;  Ye, 1993 | N/A | Kuramoto, 1977 | N/A | N/A |
| Ranidae | *Pelophylax plancyi* | Fei, 2009 | Fei, 2009 | Fei, 2009 | Fei, 2009 | N/A | N/A | Fei, 2009 |
| Ranidae | *Pelophylax ridibundus* | N/A | Fei, 2009 | Fei, 2009 | Fei, 2009 | N/A | N/A | Fei, 2009 |
| Ranidae | *Pseudorana weiningensis* | Fei, 2009 | Fei, 2009 | Fei, 2009 | Fei, 2009 | N/A | N/A | Fei, 2009 |
| Ranidae | *Rana adenopleura* | Yang, 1991 | Liu, 1950;  Pope, 1931;  Yang, 1991;  Ye, 1993 | Chen, 1991;  Huang, 1990;  Liu, 1950;  Yang, 1991;  Ye, 1993 | N/A | Xu, 2005 | Liu, 1950 | N/A |
| Ranidae | *Rana amurensis* | Pope, 1931 | Kuzmin, 1999 | Kuzmin, 1999 | N/A | AmphibiaWeb | N/A | N/A |
| Ranidae | *Rana arvalis* | N/A | Kuzmin, 1999 | Inger, 1968;  Kuzmin, 1999 | Wells, 1977 | N/A | N/A | N/A |
| Ranidae | *Rana asiatica* | Pope, 1931 | Kuzmin, 1999 | Kuzmin, 1999 | N/A | Kuzmin, 1999 | N/A | N/A |
| Ranidae | *Rana aurora* | Wright, 1995 | Wright, 1995 | Nussbaum, 1983 | Wells, 1977 | N/A | N/A | N/A |
| Ranidae | *Rana boylii* | Nussbaum, 1983 | Nussbaum, 1983 | Nussbaum, 1983;  Stebbins, 1951;  Wright, 1995 | N/A | Wright, 1995 | N/A | N/A |
| Ranidae | *Rana cascadae* | Wright, 1995 | Nussbaum, 1983;  Stebbins, 1951 | Nussbaum, 1983 | N/A | Nussbaum, 1983;  Wright, 1995 | N/A | N/A |
| Ranidae | *Rana chaochiaoensis* | Yang, 1991 | Fei, 2009 | N/A | N/A | AmphibiaWeb | N/A | N/A |
| Ranidae | *Rana chensinensis* | Okada, 1966 | N/A | Inger, 1968;  Ye, 1993 | N/A | Ye, 1993 | N/A | Ye, 1993 |
| Ranidae | *Rana dalmatina* | Lode, 2005 | Kuzmin, 1999 | Kuzmin, 1999 | Lode, 2005 | N/A | N/A | N/A |
| Ranidae | *Rana dybowskii* | N/A | Kuzmin, 1999 | Kuzmin, 1999 | N/A | AmphibiaWeb | N/A | AmphibiaWeb |
| Ranidae | *Rana graeca* | N/A | AmphibiaWeb | AmphibiaWeb | N/A | AmphibiaWeb | AmphibiaWeb | N/A |
| Ranidae | *Rana huanrenensis* | Fei, 2009 | Fei, 2009 | Fei, 2009 | N/A | Fei, 2009 | N/A | Fei, 2009 |
| Ranidae | *Rana japonica* | Pope, 1931 | Huang, 1990;  Okada, 1966 | Chen, 1991 | Wells, 1977 | N/A | N/A | N/A |
| Ranidae | *Rana johnsi* | Fei, 2009 | Fei, 2009 | Fei, 2009 | N/A | Fei, 2009 | N/A | Fei, 2009 |
| Ranidae | *Rana kukunoris* | Fei, 2009 | Fei, 2009 | Fei, 2009 | N/A | Fei, 2009 | N/A | Fei, 2009 |
| Ranidae | *Rana kunyuensis* | Fei, 2009 | Fei, 2009 | Fei, 2009 | N/A | Fei, 2009 | N/A | Fei, 2009 |
| Ranidae | *Rana longicrus* | Fei, 2009 | N/A | Fei, 2009 | N/A | Fei, 2009 | N/A | Fei, 2009 |
| Ranidae | *Rana luteiventris* | N/A | Wright, 1995 | Wright, 1995 | N/A | Stebbins, 2003 | N/A | N/A |
| Ranidae | *Rana macrocnemis* | N/A | N/A | Kuzmin, 1999 | N/A | Kuzmin, 1999 | N/A | N/A |
| Ranidae | *Rana muscosa* | Wright, 1995 | Wright, 1995 | Stebbins, 1951;  Wright, 1995 | N/A | Wright, 1995 | N/A | N/A |
| Ranidae | *Rana omeimontis* | N/A | Ye, 1993 | N/A | N/A | Ye, 1993 | N/A | N/A |
| Ranidae | *Rana ornativentris* | Okada, 1966 | Okada, 1966 | Okada, 1966 | N/A | AmphibiaWeb | N/A | N/A |
| Ranidae | *Rana pretiosa* | Fei, 2009 | Fei, 2009 | Fei, 2009 | Fei, 2009 | N/A | N/A | Fei, 2009 |
| Ranidae | *Rana sauteri* | Fei, 2009 | Fei, 2009 | Fei, 2009 | N/A | Fei, 2009 | N/A | Fei, 2009 |
| Ranidae | *Rana shuchinae* | Yang, 1991 | N/A | Yang, 1991 | N/A | Yang, 1991 | N/A |  |
| Ranidae | *Rana tagoi* | Fei, 2009 | Fei, 2009 | N/A | N/A | N/A | N/A | Fei, 2009 |
| Ranidae | *Rana temporaria* | Yang, 1991 | Morrison, 2003 | Morrison, 2003 | Wells, 1977 | N/A | N/A | N/A |
| Ranidae | *Rana tsushimensis* | Okada, 1966 | AmphibiaWeb | AmphibiaWeb | N/A | AmphibiaWeb | N/A | AmphibiaWeb |
| Ranidae | *Rana zhenhaiensis* | Fei, 2009 | Fei, 2009 | Fei, 2009 | Fei, 2009 | N/A | N/A | Fei, 2009 |
| Ranidae | *Staurois latopalmatus* | Inger, 1966 | N/A | N/A | N/A | N/A | N/A | N/A |
| Ranidae | *Staurois natator* | Inger, 1966 | Inger, 1966 | N/A | N/A | AmphibiaWeb | N/A | AmphibiaWeb |
| Rhacophoridae | *Buergeria buergeri* | Okada, 1966 | Okada, 1966 | N/A | N/A | N/A | N/A | N/A |
| Rhacophoridae | *Buergeria japonica* | Okada, 1966 | Fei, 1999 | N/A | N/A | Fei, 1999 | N/A | Fei, 1999 |
| Rhacophoridae | *Buergeria oxycephala* | Ye, 1993  Pope, 1931 | Ye, 1993 | N/A | N/A | N/A | N/A | N/A |
| Rhacophoridae | *Buergeria robusta* | Fei, 2009 | Fei, 2009 | N/A | N/A | Fei, 2009 | N/A | Fei, 2009 |
| Rhacophoridae | *Chirixalus doriae* | Pope, 1931 | Fei, 1999 | N/A | N/A | Fei, 1999 | N/A | Fei, 1999 |
| Rhacophoridae | *Chirixalus vittatus* | Yang, 1991 | Hu, 1987 | Hu, 1987;  Ye, 1993 | N/A | Hu, 1987 | N/A | Fei, 1999 |
| Rhacophoridae | *Chiromantis xerampelina* | Schiotz, 1999 | Wager, 1965;  Yang, 1991 | Wager, 1965;  Yang, 1991 | Pough, 2001 | N/A | Beck, 1998;  Duellman, 1994 | N/A |
| Rhacophoridae | *Gracixalus gracilipes* | Yang, 1991 | N/A | N/A | N/A | N/A | N/A | AmphibiaWeb |
| Rhacophoridae | *Kurixalus eiffingeri* | Fei, 2009 | Fei, 2009 | Fei, 2009 | N/A | Fei, 2009 | Fei, 2009 | N/A |
| Rhacophoridae | *Philautus aurifasciatus* | Inger, 1966  Dring, 1979 | Alcala, 1986 | Alcala, 1986 | N/A | N/A | N/A | N/A |
| Rhacophoridae | *Polypedates leucomystax* | Inger, 1966 | Yang, 1991 | Yang, 1991 | N/A | Marquez, 2006 | N/A | Yang, 1991 |
| Rhacophoridae | *Polypedates megacephalus* | Fei, 2009 | Fei, 2009 | Fei, 2009 | N/A | Fei, 2009 | N/A | Fei, 2009 |
| Rhacophoridae | *Polypedates mutus* | Yang, 1991 | Yang, 1991 | N/A | N/A | N/A | N/A | N/A |
| Rhacophoridae | *Rhacophorus arboreus* | Okada, 1966 | Okada, 1966  Pope, 1931 | Okada, 1966  Pope, 1931 | N/A | N/A | N/A | N/A |
| Rhacophoridae | *Rhacophorus bipunctatus* | N/A | N/A | N/A | N/A | Dring, 1979 | N/A | AmphibiaWeb |
| Rhacophoridae | *Rhacophorus dennysi* | Ye, 1993  Chen, 1991  Huang, 1990  Pope, 1931 | Ye, 1993  Huang, 1990 | Ye, 1993 | N/A | Pope, 1931 | N/A | Chen, 1991 |
| Rhacophoridae | *Rhacophorus dugritei* | Fei, 2009 | Fei, 2009 | Fei, 2009 | N/A | Fei, 2009 | N/A | Fei, 2009 |
| Rhacophoridae | *Rhacophorus feae* | Fei, 2009 | N/A | N/A | N/A | Fei, 2009 | N/A | Fei, 2009 |
| Rhacophoridae | *Rhacophorus kio* | Fei, 2009 | Fei, 2009 | N/A | Fei, 2009 | N/A | N/A | Fei, 2009 |
| Rhacophoridae | *Rhacophorus moltrechti* | Fei, 2009 | Fei, 2009 | Fei, 2009 | N/A | Fei, 2009 | N/A | Fei, 2009 |
| Rhacophoridae | *Rhacophorus nigropunctatus* | Fei, 2009 | Fei, 2009 | N/A | N/A | Fei, 2009 | N/A | Fei, 2009 |
| Rhacophoridae | *Rhacophorus omeimontis* | Fei, 2009 | Fei, 2009 | N/A | N/A | Fei, 2009 | Fei, 2009 | N/A |
| Rhacophoridae | *Rhacophorus reinwardtii* | Yang, 1991  Ye, 1993 | Pope, 1931 | Pope, 1931 | N/A | Dring, 1979 | N/A | N/A |
| Rhacophoridae | *Rhacophorus rhodopus* | Fei, 2009 | N/A | Fei, 2009 | N/A | Fei, 2009 | N/A | Fei, 2009 |
| Rhacophoridae | *Rhacophorus schlegelii* | Inger, 1947  Okada, 1966 | Okada, 1966 | Okada, 1966 | N/A | N/A | N/A | N/A |
| Rhinophrynidae | *Rhinophrynus dorsalis* | N/A | Savage, 2002 | Savage, 2002 | N/A | Savage, 2002 | N/A | Savage, 2002 |
| Scaphiopodidae | *Scaphiopus couchii* | Wright, 1995 | Wright, 1995 | Degenhardt, 1996 | N/A | Wright, 1995 | N/A | Wright, 1995 |
| Scaphiopodidae | *Scaphiopus holbrookii* | Wright, 1995 | Hulse, 2001 | N/A | N/A | Wright, 1995 | N/A | Wright, 1995 |
| Scaphiopodidae | *Scaphiopus hurterii* | Wright & Wright, 1949 | Wright & Wright, 1949 | N/A | N/A | Wright & Wright, 1949 | N/A | N/A |
| Scaphiopodidae | *Spea bombifrons* | Wright & Wright, 1949 | N/A | Smith, 1950  Wright & Wright, 1949  Hoser, 1989 | N/A | Wright & Wright, 1949 | N/A | N/A |
| Scaphiopodidae | *Spea hammondii* | Wright, 1995 | Stebbin, 1951 | Stebbin, 1951 | N/A | Stebbin, 1951 | N/A | Wright, 1995 |
| Scaphiopodidae | *Spea intermontana* | Wright & Wright, 1949  Nussbaum, 1983 | N/A | Nussbaum, 1983  Stebbin, 1951 | N/A | Wright & Wright, 1949 | N/A | N/A |
| Sooglossidae | *Sooglossus sechellensis* | AmphibiaWeb | N/A | N/A | N/A | N/A | AmphibiaWeb | N/A |
| Strabomantidae | *Oreobates quixensis* | Duellman, 1996 | Crump, 1974 | Crump, 1974 | N/A | N/A | N/A | N/A |
| Strabomantidae | *Strabomantis bufoniformis* | Savage, 2002 | N/A | N/A | N/A | Savage, 2002 | N/A | Savage, 2002 |

**References:**

Abrunhosa, P. A., and Wogel, H. 2004. Breeding behavior of the leaf-frog *Phyllomedusa burmeisteri* (Anura: Hylidae). *Amphibia-Reptilia* **25**:125-135.

Alcala, A. C. 1986. *Guide to Philippine Flora and Fauna: Amphibians and Reptiles.* Natural Resources Management Center and University of the Philippines, Manilla.

Bagnara, J. T., Iela, L., Morrisett, F., and Rastogi, R. K. 1986. Reproduction in the Mexican leaf frog (*Pachymedusa dacnicolor*) I. Behavioral and morphological aspects. *Occasional Papers of the Museum of Natural History, The University of Kanasa, Lawrence, Kansas* **121**:1-31.

Bain, R. H., Lathrop, A., Murphy, R. W., Orlov, N. L., and Ho, T. C. 2003. Cryptic species of a cascade frog from Southeast Asia: taxonomic revisions and descriptions of six new species. *American Museum novitiates* **3417**:1-60.

Bartlett, R. D., and Bartlett, P. 2003. *Reptiles and Amphibians of the Amazon.*University Press of Florida, Gainesville.

Bastos, R. P., and Haddad, C. F. B. 1996. Breeding activity of the neotropical treefrog *Hyla elegans* (Anura, Hylidae). *Journal of Herpetology* **30**:355-360.

Beck, C. W. 1998. Mode of fertilization and parental care in anurans. *Animal Behavior* **55**:439-449.

Beebee, T. J. C., and Griffiths, R. A. 2000. *Amphibians and Reptiles-A Natural History of the British Herpetofauna.* HarperCollins Publishers, London.

Bernal, X. E., Guarnizo, C., and Luddecke, H. 2005. Geographic variation in advertisement call and genetic structure of *Colostethus palmatus* (Anura, Dendrobatidae) from the Colombian Andes. *Herpetologica* **61**:395-408.

Bickford, D. P. 2004. Differential parental care behaviors of arboreal and terrestrial microhylid frogs from Papua New Guinea. *Behavioral Ecology and Sociobiology* **55**:402–409.

Blommers-Schlosser, R. M. A. 1979. Biosystematics of the Malagasy frogs II. The genus *Boophis* (Rhacophoridae). *Bijdragen tot de Dierkunde* **19**:261-312.

Bogert, C. M. 1962. Isolation mechanisms in toads of the *Bufo debilis* group in Arizona and western Mexico. *American Museum Novitates* **2100**:1-37.

Bourne, G. R., Collins, A. C., Holder, A. M., and McCarthy, C. L. 2001. Vocal communication and reproductive behavior of the frog *Colostethus beebei* in Guyana. *Journal of Herpetology* **35**:272-281.

Brown, J. L., Twomey, E., Morales, V., and K. Summers. 2008. Phytotelm size in relation to parental care and mating strategies in two species of Peruvian poison frogs. *Behaviour* **145**:1139-1165.

Brzoska, J., Schneider, H., and Nevo, E. 1982. Territorial behavior and vocal responses in male *Hyla arborea savignyi* (Amphibia: Anura). *Israel Journal of Zoology* **31**:27-37.

Bush, S. L. 1996. Why is double clutching rare in the Majorcan midwife toad? *Animal Behavior* **52**:913-922.

Busse, K. 2002. Is the Chile Darwin’s frog (*Rhinoderma rufum*) still alive? *The Web Site of Huina-pukios*. http://www.geocities.com/biodiversidadchile/rhinorufum.htm. (Accessed on January 2, 2008)

Caldwell, J. P. 1974. A re-evaluation of the *Hyla bistincta* species group, with descriptions of three new species (Anura: Hylidae). *Occasional Papers of the Museum of Natural History, University of Kansas* **28**:1-37.

Caldwell, J. P., and de Araujo, M. C. 2004. Historical and ecological factors influence survivorship in two clades of phytotelm-breeding frogs (Anura: Bufonidae, Dendrobatidae). *Miscellaneous Publications, Museum of Zoology, University of Michigan* **193**:11-21.

Caldwell, J. P., and de Oliveira, V. R. L. 1999. Determinants of biparental care in the spotted poison frog, *Dendrobates vanzolinii* (Anura: Dendrobatidae). *Copeia* **1999**:565-575.

Caldwell, J. P., Lima, A. P. 2003. A new Amazonian species of *Colostethus* (Anura: Dendrobatidae) with a nidicolous tadpole. *Herpetologica* **59**:219-234.

Caramaschi, U. 2003. A new species of the *Bufo margaritifer* (Laruenti, 1768) complex from the State of Mato Grosso do Sul, Brazil (Amphibia, Anura, Bufonidae). *Boletim Do Museu Nacional-Zoologia* **501**:1-16.

Cei, J. M. 1980. *Amphibians of Argentina.* Monitore Zoologico Italiano, Florence.

Channing, A. 2001. *Amphibians of Central and Southern Africa.* Cornell University Press, Ithaca.

Channing, A., Moyer, D. C., and Howell, K. M. 2002. Description of a new torrent frog in the genus *Arthroleptides* from Tanzania (Amphibia, Anura, Ranidae). *Alytes* **20**:13-27.

Chen, B. H. 1991. *The Amphibian and Reptilian Fauna of Anhui*. Publishing House of Science and Technology, Hefei, Anhui.

Cherry, M. I. 1992. Sexual selection in the leopard toad, *Bufo pardalis*. *Behavior* **120**:164-176.

Cocroft, R. B., and Ryan, M. J. 1995. Patterns of advertisement call evolution in toads and chorus frogs. *Animal Behavior* **49**:283-303.

Coloma, L. A. 1995. Ecuadorian frogs of the genus *Colostethus* (Anura: Dendrobatidae). *University of Kansas,* *Museum of Natural History,* *Miscellaneous Publication* **87**:1-72.

Cook, C. L., Ferguson, J. W. H., and Telford, S. R. 2001. Adaptive male parental care in the giant bullfrog, *Pyxicephalus adspersus*. *Journal of Herpetology* **35**:310-315.

Crump, M. L. 1974. Reproductive strategies in a tropical anuran community. *University of Kansas Museum of Natural History, University of Kansas, Lawrence, Miscellaneous Publication* **61**:1-68.

Crump, M. L., and Townsend D. S. 1990. Random mating by size in a neotropical treefrog, *Hyla pseudopuma*. *Herpetologica* **46**:383-386.

Degenhardt, W. G., Painter, C. W., and Price, A. H. 1996. *Amphibians and Reptiles of New Mexico.* University of New Mexico Press, Albuquerque.

Diesel, R., Baurle, G., Vogel, P . 1995. Cave breeding and froglet transport: a novel pattern of anuran brood. Care in the Jamaican frog, *Eleutherodactylus cundalli*. *Copeia* **1995**:354-360.

Digital Library Project. 2008. *AmphibiaWeb: Information on Amphibian Biology and Conservation*. http://amphibiaweb.org/.(Accessed on January 2, 2008)

Dring, J. C. M. 1979. Amphibians and reptiles from northern Trengganu, Malaysia, with descriptions of two new geckos, *Cnemaspis* and *Cyrtodactylus*.

*Bulletin of the British Museum (Natural History)* **34**:181-240.

Duellman, W. E. 1956. The frogs of the hylid genus *Phrynohyas* Fitzinger, 1843. *Miscellaneous Publications, Museum of Zoology, University of Michigan* **96**:1-47.

Duellman, W. E. 1966. Aggressive behavior in dendrobatid frogs. *Herpetologica* **22**:217-221.

Duellman, W. E. 1972. A review of the neotropical frogs of the *Hyla bogotensis* group. *Occasional Papers of the Museum of Natural History, The University of Kansas, Lawrence, Kansas* **11**:1-31.

Duellman, W. E. 1976. The systematic status and relationships of the hylid frog *Nyctimantis Rugiceps* Boulenger. *Occasional Papers of the Museum of Natural History, The University of Kansas, Lawrence, Kansas* **58**:1-14.

Duellman, W. E. 1982. A new species of small yellow *Hyla* from Peru (Anura: Hylidae). *Amphibia-Reptilia* **3**:153-160.

Duellman, W. E. 1989. New species of hylid frogs from the Andes of Columbia and Venezuela. *Occasional Papers of the Museum of Natural History, The University of Kansas, Lawrence, Kansas* **131**:1-12.

Duellman, W. E. 1996. Anuran amphibians from a seasonally dry forest in southeastern Peru and comparisons of the anurans among sites in the upper Amazon Basin. *Occasional Papers of the Museum of Natural History, The University of Kansas, Lawrence, Kansas* **180**:1-34.

Duellman, W. E. 1997. Amphibians of La Escalera Region, southeastern Venezuela: Taxonomy, Ecology and Biogeography. *Scientific Papers* **2**:1-52.

Duellman, W. E. 2004. Frogs of the Genus *Colostethus* (Anura; Dendrobatidae) in the Andes of northern Peru. *Scientific Papers Natural History Museum University of Kansas* **34**:1-49.

Duellman, W. E., and Campbell, J. A. 1992. Hylid frogs of the genus *Plectrohyla*: systematics and phylogenetic relationships. *Miscellaneous Publications, Museum of Zoology, University of Michigan* **181**:1-32.

Duellman, W. E., and Crump, M. L. 1974b. Speciation in frog of the *Hyla parviceps* group in the upper Amazon Basin. *Occasional Papers of the Museum of Natural History, The University of Kansas, Lawrence, Kansas* **23**:1-40.

Duellman, W. E., and Dennis, D. M. 2001. *The hylid frogs of Middle America.* Society for the Study of Amphibians and Reptiles, Kansas.

Duellman, W. E., and Hoogmoed, M. S. 1984. The taxonomy and phylogenetic relationships of the hylid frog genus *Stefania*. *The University of Kansas Muesum of Natural History* **75**:1-39.

Duellman, W. E., and Trueb, L. 1994. *Biology of Amphibians*. The Johns Hopkins University Press, Baltimore and London.

Duellman, W. E., and Veloso, A. 1977. Phylogeny of *Pleurodema* (Anura: Leptodactylidae): a biogeographic model. *Occasional Papers of the Museum of Natural History, The University of Kansas, Lawrence, Kansas* **64**:1-46.

Duellman, W. E., and Wild, E. R. 1993. Anuran amphibians from the Cordillera de Huancabamba, northern Peru: Systematics, Ecology and biogeography. *Occasional Papers of the Museum of Natural History, The University of Kansas, Lawrence, Kansas* **157**:1-53.

Dunn, E. R. 1941. Notes on *Dendrobates auratus*. *Copeia* **1941**:88-92.

Dyson, M. L., Henzi, S. P., Halliday, T. R., and Barrett, L. 1998. Success breeds success in mating male reed frogs (*Hyperolius marmoratus*). *Proceedings of the Royal Society B: Biological Sciences* **265**:1417-1421.

Edwards, S. R. 1971. Taxonomic notes on South American *Colostethus* with descriptions of two new species (Amphibia, Dendrobatidae). *Proceedings of the Biological Society of Washington* **84**:147-162.

Eens, M., and Pinxten, R. 2000. Sex-role reversal in vertebrates: behavioral and endocrinological accounts. *Behavioral Processes* **51**:135-147.

Emerson, S. B., and Voris, H. 1992. Competing explanations for sexual dimorphism in a voiceless Bornean frog. *Functional Ecology* **6**:654-660.

Fei, L., Ye, C. Y., Huang, Y. S., Liu, M. Y., Wang, Y. S., and Li, J. 1999. *Atlas of Amphibians of China.* Henan Publishing House of Science and Technology, Zhengzhou.

Fei, L., Hu, S., Ye C., Huang, Y. *et al*., 2009. *Fauna Sinica, Amphibia*. *Vol 2 & 3, Anura*. Science Press, Beijing (in Chinese).

Fellers, G. M. 1979. Aggression, territoriality and mating behavior in North American treefrogs. *Animal Behavior* **27**:107-119.

Frost, J. S., and Bagnara, J. T. 1977. Sympatry between *Rana blairi* and the southern form of leopard frog in southeastern Arizona (Anura:Ranidae). *Southwest Naturalist* **22**:443-453.

Garton, J. S., and Brandon, R. A. 1975. Reproductive ecology of the green treefrog, *Hyla cinerea*, in southern Illinois. *Herpetologica* **31**:150-161.

Given, M. F. 1988. Territoriality and aggressive interactions of male carpenter frogs, *Rana virgatipes*. *Copeia* **1988**:411-421.

Given, M. F. 2002. Interrelationships among calling effort, growth rate and chorus tenure in *Bufo fowleri*. *Copeia* **2002**:979-987.

Glaw, F., Vences, M., and Gossmann, V. 2000. A new species of *Mantidactylus* (subgenus *Guibemantis*) from Madagascar, with a comparative survey of internal femoral gland structure in the genus (Amphibia: Ranidae: Mantellinae). *Journal of Natural History* **34**:1135-1154.

Godwin, G. J., and Roble, S. 1983. Mating success in male treefrogs, *Hyla chrysoscelis* (Anura: Hylidae). *Herpetologica* **39**:141-146.

Goin, C. J., and Netting, M. G. 1940. A new gopher frog from the Gulf Coast, with comments upon the *Rana areolata* group. *Annals of the Carnegie Museum* **28**:137-169.

Gottsberger, B., and Gruber, E. 2004. Temporal partitioning of reproductive activity in a neotropical anuran community. *Journal of Tropical Ecology* **20**:271-280.

Gramapurohit, N. P., Shanbhag, B. A., and Saidapur, S. K. 2005. Post-metamorphic growth, sexual maturation and body size dimorphism in the skipper frog, *Euphlyctis cyanophlyctis* (Schneider). *Herpetological Journal* **15**:113-119.

Grant, T., Bolivar-G., W., and Castro, F. 1998. The advertisement call of *Centrolene geckoideum*. *Journal of Herpetology* **32**:452-455.

Haddad, C. F. B., Faivovich, J., and Garcia, P. C. A. 2005. The specialized reproductive mode of the treefrog *Aplastodiscus perviridis* (Anura: Hylidae). *Amphibia-Reptilia* **26**:87-92.

Hartmann, M. T., Hartmann, P. A., and Haddad, C. F. B. 2004. Visual signaling and reproductive biology in a nocturnal treefrog, genus *Hyla* (Anura: Hylidae). *Amphibia-Reptilia* **25**:395-406.

Hartmann, M. T., Giasson, L. O. M., Hartmann, P. A., and Haddad, C. F. B. 2005. Visual communication in Brazilian species of anurans from the Atlantic forest. *Journal of Natural History* **39**:1675–1685.

Hermans, K., Pinxten, R., and Eens, M. 2002. Territorial and vocal behavior in a captive dart-poison frog, *Epipedobates tricolor* Boulenger, 1899 (Anura: Dendrobatidae). *Belgian Journal of Zoology* **132**:105-109.

Hettyey, A., Torok, J., and Hevizi, G. 2005. Male mate choice lacking in the agile frog, *Rana dalmatina*. *Copeia* **2**:403-408

Heyer, W. R. 1973. Systematics of the *Marmoratus* group of the frog genus *Leptodactylus* (Amphibia, Leptodactylidae). *Natural History Museum* 25:1-50.

Heyer, W. R. 1978. Systematics of the *fuscus* group of the frog genus *Leptodactylus* (Amphibia, Leptodactylidae). *National History Museum of Los Angeles County* **29**:1-85.

Heyer, W. R. 1994. Variation within the *Leptodactylus podicipinus*-wagneri complex of frogs (Amphibia: Leptodactylidae). *Smithsonian Contributions to Zoology* **546**.

Heying, H. E. 2001. Social and reproductive behaviour in the Madagascan poison frog, *Mantella laevigata*, with comparisons to the dendrobatids. *Animal Behaviour* **61**:567–577.

Heying, H. 2004. Reproductive limitation by oviposition site in a treehole breeding Madagascan poison frog (*Mantella laevigata*). *Miscellaneous Publications Museum of Zoology University of Michigan* **193**:23-30.

Honolulu Zoo. 2008. *Honolulu Zoo: Surinam Toad*. http://www.honoluluzoo.org/surinam toad.htm. (Accessed on January 2, 2008)

Hoser, R. T. 1989. *Australian Reptiles and Frogs.* Pierson, Sydney.

Howard, R. D. 1988. Sexual selection on male body size and mating behaviour in American toads, *Bufo americanus*. *Animal Behaviour* **36**: 1796-1808.

Hu, S. Q., Fei, L., Hu Q. X., Huang, Q. Y., Huang, Y. Z., Jiang, Y. M., Tian, W. S., Ye, C. Y., and Zhao, E. M. 1987. *The Series of the Scientific Expedition to the Qinghai-Xizang Plateau.* Science Press, Beijing.

Huang, M. H., Jin, Y. L., and Cai, C. M. 1990. *Fauna of Zhejiang: Amphibia, Reptilia.* Zhejiang Science and Technology Publishing House, Hangzhou.

Hulse, A. C., McCoy, D. J., and Densky, E. J. 2001. *Amphibians and Reptiles of Pennsylvania and the Northeast.* Comstock Publishing Associates, Cornell University Press, Ithaca.

Inger, R. F. 1947. Preliminary survey of the amphibians of the Riukiu Islands. *Fieldiana: Zoology* **32**:297-352.

Inger, R. F. 1954. Systematics and Zoogeography of Philippine Amphibia. *Fieldiana: Zoology* **33**:181-531.

Inger, R. F. 1966. The systematics and zoogeography of the Amphibia of Borneo. *Fieldiana: Zoology* **52**:1-402.

Inger, R. F., and Bacon, J. P. Jr. 1968. Annual reproduction and clutch size in rain forest frogs from Sarawak. *Copeia* **968**:602-606.

Inger, R. F., and Kottelat, M. 1998. A new species of ranid frog from Laos. *The Raffles Bulletin of Zoology* **46**:29-34.

IUCN, Conservation International and Nature Serve. 2006. *Global Amphibian Assessment*. http://www.globalamphibians.org. (Accessed on January 2, 2008)

Jowers, M. J., and Downie, J. R. 2005. Tadpole deposition behavior in male stream frogs *Mannophryne trinitatis* (Anura: Dendrobatidae). *Journal of Natural History* **39**:3013-3027.

Junca, F. A., and Rodrigues, M. T. 2006. The reproductive success of *Colostethus stepheni* (Anura: Dendrobatidae). *Studies on Neotropical Fauna and Environment* **41**:9-17.

Jungfer, K-H. 1985. Beitrag zur kenntnis von *Dendrobates* *speciosus* O. Schmidt, 1857 (Salientia: Dendrobatidae). *Salamandra* **21**:263-280.

Jungfer, K-H., and Weygoldt, P. 1999. Biparental care in the tadpole-feeding Amazonian treefrog *Osteocephalus oophagus*. *Amphibia-Reptilia* **20**:235-249.

Katsikaros, K., and Shine, R. 1997. Sexual dimorphism in the tusked frog, *Adelotus brevis* (Anura: Myobatrachidae): the roles of natural and sexual selection. *Biological Journal of the Linnean Society* **60**:39-51.

Keith, R. 1968. A new species of Bufo from Africa with comments on the toads of the *Bufo regularis* complex. *American Museum Novitates* **2345**:1-22.

Kubicki, B. 2007. *Glass Frogs of Costa Rica*. Santo Domingo de Heredia, Costa Rica: Instituto Nacional de Biodiversidad.

Knowles, R., Mahony, M., Armstrong, J., and Donnellan, S. 2004. Systematics of sphagnum frogs of the genus *Philoria* (Anura: Myobatrachidae) in Eastern Australia, with the description of two new species. *Records of the Australian Museum* **56**:57-74.

Kuramoto, M. 1977. Mating call structures of the Japanese pond frogs, *Rana nigromaculata* and *Rana brevipoda* (Amphibia, Anura, Ranidae). *Journal of Herpetology* **11**:249-254.

Kuzmin, S. L. 1999. *The Amphibians of the Former Soviet Union.* Pensoft, Sofia-Moscow.

Lardner, B., and Lakim, M. B. 2004. Female call preferences in tree-hole frogs: why are there so many unattractive males? *Animal Behaviour* **68**:265-272.

Lathrop, A., Murphy, R. W., Orlov, N., and Ho, C. T. 1998. Two new species of *Leptolalax* (Anura: Megophryidae) from northern Vietnam. *Amphibia-Reptilia* **19**:253-267.

Lee, A. K. 1967. Studies in Australian Amphibia II. Taxonomy, ecology, and evolution of the genus *Heleioporus* Gray (Anura: Leptodactylidae). *Australian Journal of Zoology* **15**:367-439.

Lee, J. C. 2000. *A Field Guide to the Amphibians and Reptiles of the Maya World.* Cornell University Press, Ithaca.

Lehr, E, Koehler, H., Aguilar, C., and Ponce, E. 2001. New Species of *Bufo* (Anura: Bufonidae) from central Peru. *Copeia* **18**:216-223.

Lima, A. P., Caldwell, J. P., and Biavati, G. M. 2002. Territorial and reproductive behavior of an Amazonian dendrobatid frog, *Colostethus caeruleodactylus*. *Copeia* **1**:44-51.

Liu, C. Z. 1950. *Amphibians of Western China.* Chicago Natural History Museum, Chicago.

Lode, T., Holveck, M. J., and Lesbarreres, D. 2005. Asynchronous arrival pattern, operational sex ratio and occurrence of multiple paternities in a territorial breeding anuran, *Rana dalmatina*. *Biological Journal of the Linnean Society* **86**:191-200.

Loveridge, A. 1936. Scientific results of an expedition to rain forest regions in eastern Africa. VII. Amphibians. *Bulletin of the Museum of Comparative Zoology, Harvard College* **79**:369-430.

Lutz, B. 1973. *Brazilian Species of Hyla.* University of Texas Press, Austin and London.

Lynch, J., Duellman, W. E. 1973. A review of the centrolenid frogs of Ecuador, with descriptions of new species. *Occasional Papers of the Museum of Natural History, The University of Kansas, Lawrence, Kansas* **16**:1-66.

Main, A. R. 1965. *Frogs of Southern Western Australia.* Western Australia Naturalist’s Club, Perth.

Marquez, R. 1993. Male reproductive success in two midwife toads, *Alytes obstetricans* and *A. cisternasii*. *Behavioral Ecology and Sociobiology* **32**:283-291.

Marquez, R. 2006. Advertisement calls of six species of anurans from Bali, Republic of Indonesia. *Journal of Natural History* **40**:571-588.

Martins, M. 1988. Reproductive biology of *Leptodactylus fuscus* in Boa Vista, Roraima (Amphibia: Anura). *Revista Brasileira de biologia* **48**:969-977.

Martins, M., Pombal, J. P. Jr., and Haddad, C. F. B. 1998. Escalated aggressive behavior and facultative parental care in the nest building gladiator frog *Hyla faber* Wied. *Amphibia-Reptilia* **19**:65-73.

Mendelson, J. R. III, Williams, B. L., Sheil, C. A., and Mulcahy, D.G. 2005 Systematics of the Bufo coccifer complex (Anura: Bufonidae) of Mesoamerica. *Scientific Papers Natural History Museum the University of Kansas* **38**:1-27.

Menzies, J. I. 1976. *Handbook of Common New Guinea frogs.* Wau Ecology Institute, Papua New Guinea.

Mitchell, N. J. 2002. Nest-site selection in a terrestrially breeding frog with protracted development. *Australian Journal of Zoology* **50**:225-235.

Moore, J. A. 1961. *The Frogs of Eastern New South Wales.* American Museum of Natural History, New York.

Morrison, C., and Hero, J-M. 2003. Geographic variation in life-history characteristics of amphibians: a review. *Journal of Animal Ecology* **72**:270-279.

Murphy, J. F., Simandle, E. T., and Becker, D. E. 2003. Population status and conservation of the black toad, *Bufo exsul*. *Southwestern Naturalist* **48**:54-60.

Myers, C. W. and Bohme, W. 1996. On the type specimens of two Colombian poison frogs described by A. A. Berthold (1845) and their bearing on the locality “Provinz Popayan”. *American Museum Novitates* **3185**:1-20.

Myers, C. W. 1982. Spotted poison frogs: descriptions of three new *Dendrobates* from western Amazonia and resurrection of a lost species from “Chiriqui”. *American Museum Novitates* **2721**:1-23.

Myers, C. W., and Daly, J. W. 1976. A new species of poison frog (*Dendrobates*) from Andean Ecuador, including an analysis of its skin toxins. *Occasional Papers of the Natural History Museum, the University of Kansas* **59**:1-12.

Myers, C. W., and Daly, J. W. 1979. A name for the poison frog of Cordillera Azul, Eastern Peru, with notes on its biology and skin toxins (*Dendrobatidae*). *American Museum Novitates* **2674**:1-24.

Myers, C. W., Daly, J. W., and Malkin, B. 1978. A dangerously toxic new frog (*Phyllobates*) used by Embera Indians of western Colombia, with discussion of blowgun fabrication and dart poisoning. *Bulletin of the American Museum of Natural History* **161**:309-365.

Myers, P., Espinosa, R., Parr, C. S., Jones, T., Hammond, G. S., and Dewey, T. A. 2006. *The Animal Diversity Web*. http://animaldiversity.org. (Accessed on January 2, 2008)

Navas, C. A., and Bevier, C. R. 2001. Thermal dependency of calling performance in the eurythermic frog *Colostethus subpunctatus*. *Herpetologica* **57**:384-395.

Net Industries. 2008. Vocal sac-brooding frogs: Rhinodermatidae - Darwin’s frog (*Rhinoderma darwinii*): species account. *Animal Life Resource.* http://animals.jrank.org/pages/127/Vocal-Sac-Brooding-Frogs-Rhinodermatidae-DARWIN-S-FROG-Rhinoderma-darwinii-SPECIES-ACCOUNT.html. (Accessed on January 2, 2008)

Nussbaum, R., Brodie, E. D. Jr., and Storm, R. M. 1983. *Amphibians and Reptiles of the Pacific Northwest.* University Press of Idaho, Idaho.

Ohler, A. 2004. A green-eyed *Leptobrachium* (Anura: Megophryidae) from southern Laos. *The Raffles Bulletin of Zoology* **52**:695-700.

Ohler, A., Swan, S. R., and Daltry, J. C. 2002. A recent survey of the amphibian fauna of the Cardamom Mountains, southwest Cambodia with descriptions of three new species. *The Raffles Bulletin of Zoology* **50**:465-481.

Okada, Y. 1966. *Fauna Japonica Anura (Amphibia).* Tokyo Electrical Engineering College Press, Tokyo.

Olson, D. H., Blaustein, A. R. and O'hara, R. K. 1986. Mating patterns variability among western toad (*Bufo boreas*) populations. *Oecologia* **70**: 351-356.

Ortega J. E., Serrano, V. H., and Ramírez-Pinilla, M. P. 2005. Reproduction of an introduced population of *Eleutherodactylus johnstonei* at Bucaramanga, Colombia. Copeia **2005**:642–648.

Ovaska, K., and Rand, A. S. 2001. Courtship and reproductive behavior of the frog *Eleutherodactylus diastema* (Anura: Leptodactylidae) in Gamboa, Panama. *Journal of Herpetology* **35**:44-50.

Parker, H. W. 1940. The Australiasian frogs of the family *Leptodactylidae*. *Novitates Zoologicae* **42**:1-105.

Passmore, N. I. 1981. Sound levels of mating calls of some African frogs. *Herpetologica* **37**:166-171.

Penna, M., and Veloso, A. 1990. Vocal diversity in frogs of the South American temerate forest. *Journal of Herpetology* **24**:23-33.

Pino, E. M., Venegas-Ferrín, M., Romero-Carvajal, A., Montenegro-Larrea, P., Sáenz-Ponce, N., Moya, I. M., Alarcón, I., Sudou, N., Yamamoto, S., and Taira, M. 2007. A comparative analysis of frog early development. *Proceedings of the National Academy of Sciences* **104**:11882-11888.

Poelman, E. H., and Dicke, M. 2006. Offering offspring as food to cannibals: oviposition strategies of Amazonian poison frogs (*Dendrobates ventrimaculatus*). *Evolutionary Ecology* **21**:215-227.

Pope, C. H. 1931. Notes on Amphibians from Fukien, Hainan and other parts of China. *Bulletin of the American Museum of Natural History* **61**:307-611

Pough, F. H., Andrews, R. M., Cadle, J. E., Crump, M. L., Savitzky, A. H., and Wells, K. D. 2001. *Herpetology.* Second Edition. Prentice Hall, New Jersey.

Prohl, H. 2005. Territorial behavior in dendrobatid frog. *Journal of Herpetology* **39**:354-365.

Queensland Frog Society. 2002. *Limnodynastes salmini*. *Frogs of the Greater Brisbane Region*. http://www.geocities.com/gem3007/limsalmi.htm. (Accessed on January 2, 2008)

Rabb, G. B., and Rabb, M. S. 1962. On the behavior and breeding biology of the African pipid frog *Hymenochirus boettgeri*. *Sonderdruck aus Zeitschrift fur Tierpsychologie* **20**:215-241.

Rabb, G. G., and Rabb, M. S. 1963. Additional observations on breeding behavior of the Surinam toad, *Pipa pipa*. *Copeia* **1963**:636-642.

Rao, D. Q., Wilkinson, J. A and Zhang, M. W. 2006. A new species of the genus *Vibrissaphora* (Anura: Megophryidae) from Yunnan Province, China. *Herpetologica* **62**:90-95.

Reading, C. J., and Jofré, G. M. 2003. Reproduction in the nest building vizcacheras frog *Leptodactylus bufonius* in central Argentina. *Amphibia-Reptilia* **24**:415-427.

Rivero, J. A. 1961. Salientia of Venezuela. *Bulletin Museum Comparative Zoology* **126**:1–207.

Rodrigues, D. J., Uetanabaro, M., Lopes, F. S. 2005. Reproductive patterns of *Trachycephalus venulosus* (Laurenti, 1768) and *Scinax fuscovarius* (Lutz, 1925) from the Cerrado, central Brazil. *Journal of Natural History* **39**:3217-3226.

Rodriguez, L. O., and Duellman, W. E. 1994. *Guide to the frogs of the Iquitos region, Amazonian Peru.* Lawrence: Natural History Museum, University of Kansas.

Roithmair, M. E. 1994. Male territoriality and female mate selection in the dart-poison frog *Epipedobates trivittatus* (Denderobatidae, Anura). *Copeia* **1994**:107-115.

Sabater-Pi, J. 1985. Contribution to the biology of the giant frog (Conraua goliath, Boulenger). *Amphibia-Reptilia* **6**:143-153.

Salinas, F. V. 2006. Breeding behavior and colonization success of the Cuban treefrog *Osteopilus septentrionalis*. *Herpetologica* **62**:398-408.

Savage, J. M. 1972. The systematic status of *Bufo simus* O. Schmidt with description of a new toad from western Panama. *Journal of Herpetology* **6**:25-33.

Savage, J. M. 2002. *The Amphibians and Reptiles of Costa Rica.* The University of Chicago Press, Chicago and London.

Schauble, C. S. 2004. Variation in body size and sexual dimorphism across geographical and environmental space in the frogs *Limnodynastes tasmaniensis* and *L. peronii*. *Biological Journal of the Linnean Society* **82**:39-56.

Schiesari, L., Gordo, M., and Hodl, W. 2003. Treeholes as calling, breeding and developmental sites for the Amazonian canopy Frog, *Phrynohyas resinifictrix* (Hylidae). *Copeia* **2003**:263-272.

Schiotz, A. 1975. *The Treefrogs of Eastern Africa.* Steenstrupia, Copenhagen.

Schiotz, A. 1999. *The Treefrogs of Africa.* Edition Chimaira, Frankfurt.

Schmidt, K. P. 1927. *Notes on Chinese Amphibians Bulletin of the American Museum of Natural History* **54**:553-575.

Schuierer, F. W. 1962. Remarks upon the natural history of *Bufo exsul* Myers, the endemic toad of Deep Springs Valley, Inyo County, California. *Herpetologica* **17**:260-266.

Schwartz, A., and Henderson, R. W. 1991. *Amphibians and Reptiles of the West Indies: Descriptions, Distributions and natural History*. University Press of Florida, Gainesville.

Seidel, B. 1999. Water-wave communication between territorial male *Bombina variegata*. *Journal of Herpetology* **33**:457-462.

Sexton, O. J. 1960. Some aspects of the behavior and of the territory of a dendrobatid frog, *Prostherapis trinitatis*. *Ecology* **41**:107-115.

Silva, W., Giaretta, A., and Facure, K. 2005. On the natural history of the South American pepper frog, *Leptodactylus labyrinthicus* (Spix, 1824) (Anura: Leptodactylidae). *Journal of Natural History* **39**:555–566.

Silverstone, P. A. 1975. A revision of the poison-arrow frogs of the genus *Dendrobates Wagler*. *Natural History Museum of Los Angeles County Science Bulletin* **21**:37-40.

Silverstone, P. A. 1976. A revision of the poison-arrow frogs of the genus *Phyllobates* Bibron in Sagra (family Dendrobatidae). *Natural History Museum of Los Angeles County Science Bulletin* **27**:1-53.

Smith, M. J., Roberts, J. D., Hammond, T. J., and Davis, R. A. 2003. Intraspecific variation in the advertisement call of the sunset frog *Spicospina flammocaerulea* (Anura : Myobatrachidae): A frog with a limited geographic distribution. *Journal of Herpetology* **37**:285-291.

Smith. H. M. 1950. Handbook of amphibians and reptiles of Kansas. *University of Kansas, Museum of Natural History,* *Miscellaneous Publication* **9**:1-356.

Stebbin, R. C. 2003 *A Field Guide to Western Reptiles and Amphibians*. Houghton Mifflin, Boston.

Stebbins, R. C. 1951. *Amphibians of Western North America.* University of California Press, Berkeley.

Stebbins, R. C., and Hendrickson, J. R. 1959. Field studies of amphibians in Colombia, South America. *Univeristy of California Publications in Zoology* **56**:497-540.

Stewart, M. M. 1967. *Amphibians of Malawi.* State University of New York, Albany.

Stuart, B. L. 2006. A collection of amphibians and reptiles from Hilly Eastern Cambodia. *The Raffles Bulletin of Zoology* **54**:129-155.

Sullivan, B. K. 1983. Sexual selection in the great plains toad *Bufo cognatus*. *Behaviour* **84**: 258-264.

Summers, K. 1989. Sexual selection and intra-female competition in the green poison-dart frog, *Dendrobates auratus*. *Animal Behavior* **37**:797-805.

Summers, K. 1992a. Mating strategies in two species of dart-poison frogs: a comparative study. *Animal Behavior* **43**:907-919.

Summers, K. 1992b. Essay on contemporary issues in ethology. *Ethology* **91**:89-107.

Summers, K. 2000. Mating and aggressive behavior in dendrobatid frogs from Corcovado National Park, Costa Rica: a comparative study. *Behavior* **137**:7-24.

Summers, K., McKeon, C. S., and Heying, H. 2006. The evolution of parental care and egg size: a comparative analysis in frogs. *Proceedings of Royal Society of London Series B* **273**:687-692.

Summers, K., Symula, R., Clough, M., and Cronin, T. 1999a. Visual mate choice in poison frogs. *Proceedings of Royal Society of London Series B* **266**:2141-2145.

Summers, K., and Earn, D. J. D. 1999b. The cost of polygyny and the evolution of female care in poison frogs. *Biological Journal of the Linnaean Society* **66**:515-538.

Tandy, M., and Keith, R. 1972. *Bufo* of Africa*.* In *Evolution in the Genus Bufo.* Pages 119-170 *in* Blair, W. R., ed. University of Texas Press, Austin.

Tandy, M., Tandy, J., Keith, R., and Duff-Mackay, A. 1976. A new species of *Bufo* (Anura: Bufonidae) from Africa’s dry savannas. *The Pearce-Sellards Series* **24**:1-20.

Telford, S. R. and Van Sickle, J. 1989. Sexual selection in an African toad (*Bufo gutturalis*): the roles of morphology, amplexus displacement and chorus participation. *Behaviour* **110**: 62-75.

Townsend, D. S., Stewart, M. M., and Pough, F. H. 1984. Male parental care and its adaptive significance in a Neotropical frog. *Animal Behaviour* **32**:421-431.

Trueb, L., and Duellman, W. E. 1971. A synopsis of Neotropical Hylid frogs Genus *Osteochphalus*. *Occasional Papers of the Museum of Natural History, The University of Kansas, Lawrence, Kansas*. **29**:1-47.

Trueb, L. 1974. Systematic relationships of Neotropical horned frogs, Genus *Hemiphractus* (Anura: Hylidae). *Occasional Papers of the Museum of Natural History, The University of Kansas, Lawrence, Kansas* **29**:1-60.

Trueb, L., and Cannatella, D. C.1986. Systematics, morphology and phylogeny of Genus *Pipa* (Anura: Pipidae). *Herpetologica* **42**:412-449.

Trueb, L., and Duellman, W. E. 1974. A synopsis of neotropical hylid frogs Genus *Osteochphalus*. *Occasional Papers of the Museum of Natural History, The University of Kansas, Lawrence, Kansas* **29**:1-47.

Tsuji, H. 2004. Reproductive ecology and mating success of male *Limnonectes kuhlii*, a fanged frog from Taiwan. *Herpetologica* **60**:155-167.

Tyler, M. J. 1978. *Amphibians of South Australia.* Government Printer, Adelaide.

Tyler, M. J., Crook, G. A., and Davies M. 1983. Reproductive biology of the frogs of the Magela Creek system, Northern Territory. *Records of the South Australian Museum* **18**:415-440.

Valdes, A. 1988. Systematic comments on *Peltophryne peltocephala* (Anura: Bufonidae) in the Cuban Archipelago. *Caribbean Journal of Science* **24**:39-43.

Van Doorn, M. 2002. *www.poison-frogs.com.* http://www.poison-frogs.com. (Accessed on January 2, 2008)

Vaucher, P.-Y. 2008. *Bufo mauritanicus*, le crapaud berbère ou de Maurétanie. *Batraciens et Reptiles du Monde*. http://www.batraciens-reptiles.com. (Accessed on January 2, 2008)

Vences, M., Glaw, F., and Bohme, W. 1998. Evolutionary correlates of microphagy in alkaloid-containing frogs (Amphibia: Anura). *Zoologischer Anzeiger* **236**:217-230.

Vences, M., and Glaw, F. 2001. Systematic review and molecular phylogenetic relationships of the direct developing Malagasy anurans of the *Mantidactylus asper* group (Amphibia, Mantellidae). *Alytes* **19**:107-139.

Vences, M., Glaw, F., Andreone, F., Jesu, R., and Schimmenti, G. 2002. Systematic revision of the enigmatic Malagasy broad-headed frogs (Laurentomantis Dubois, 1980), and their phylogenetic position within the endemic mantellid radiation of Madagascar. *Contributions to Zoology* **70**:191-212.

Vonesh, J. R. 2000. Dipteran predation on the arboreal eggs of four *Hyperolius* frog species in Western Uganda. *Copeia* **2000**:560-566.

Wager, V. A. 1965. *The Frogs of South Africa*. Purnell and Sons, Cape Town.

Wagner, W. E. Jr. 1989. Graded aggressive signals in Blanchard’s cricket frog: vocal responses to opponent proximity and size. *Animal Behavior* **38**:1025-1038.

Wells, K. D. 1977. The social behavior of anuran amphibians. *Animal Behavior* **25**:666-693.

Weygoldt, P. 1976. Notes on the biology and ethology of *Pipa carvalhoi* Mir. Rib. 1937 (Anura, Pipidae). *Zeitschrift fur Tierpsychologie* **40**:80-99.

Wiewandt, T. A. 1971. Breeding biology of the Mexican leaf-frog. *Fauna (Rancho Mirage, California)* **2**:29-34.

Wilbur, H. M., Rubenstein, D. I., and Fairchild, D. L. 1978. Sexual selection in toads the roles of female choice and male body size. *Evolution* **32**:264-270.

Wright, A. H., and Wright, A. A. 1949. *Handbook of Frogs and Toads of the United States and Canada.* Third Edition. Comstock Publishing Associates, Ithaca and London.

Yang, D. T. 1991. *The Amphibia-Fauna of Yunnan.* China Forestry Publishing House, Beijing.

Ye, C. Y., Fei, L., Hu, S. Q. 1993. *Rare and Economic Amphibians of China.* Sichuan Science and Technology publishing House, Chengdu.

Zimmermann, H., and Zimmermann, E. 1985. Zur Fortpflanzungsstrategie des Pfeilgiftfrosches *Phyllobates terribilis* Myers, Daly & Malkin 1978 (Salientia: Dendrobatidae). *Salamandra* **21**:281-297.

Zimmermann, H., and Zimmermann, E. 1988. Etho-Taxonomie und zoogeographische Artengruppenbildung bei Pfeilgiftfroschen. *Salamandra* **24**:125-160.

Zug, G. R., Vitt, L. J., and Galdwell, J. P. 2001. *Herpetology: An Introductory Biology of Amphibians and Reptiles*. Second Edition. Academic Press, San Diego, San Francisco, New York, Boston, London, Sydney and Tokyo.

Zweifel, R. G. 1956. Notes on microhylid frogs, genus *Cophixalus*, from New Guinea. *American Museum Novitates* **1785**:1-8.

Zweifel, R. G. 1980. Results of the archbold expeditions 103. Frogs and lizards from the Huon Peninsula Papua New-Guinea. *Bulletin of the American Museum of Natural History* **182**:265-388.
