# Supplementary material for: Does life history shape sexual size dimorphism in anurans? A comparative analysis
Source: BMC Evol Biol. 2013 Jan 31;13:27. doi: 10.1186/1471-2148-13-27 (PMC3570426; doi:10.1186/1471-2148-13-27)
Supplement: Additional file 3 — TheDstatistic for all binary traits. The non-significant p values are in bold, which means the traits are under Brownian evolution. [file 1471-2148-13-27-S3.docx]

**Additional file 3: The *D* statistic for all binary traits**. The non-significant *p* values are in bold, which means the traits are under Brownian evolution.

|  | Combat behaviour | | | | Parental care | | |
| --- | --- | --- | --- | --- | --- | --- | --- |
|  | Male combat | Female combat | Male scramble competition | Male territory defence | Parental care | Female parental care | Male parental care |
| Estimated *D* | 0.741 | 0.250 | 0.679 | 0.682 | 0.074 | 0.496 | -0.066 |
| *p* random model | <0.01 | <0.01 | <0.01 | <0.01 | <0.01 | <0.01 | <0.01 |
| *p* Brownian model | <0.01 | **0.32** | <0.01 | <0.01 | **0.36** | <0.01 | **0.64** |
