# Supplementary material for: Does life history shape sexual size dimorphism in anurans? A comparative analysis
Source: BMC Evol Biol. 2013 Jan 31;13:27. doi: 10.1186/1471-2148-13-27 (PMC3570426; doi:10.1186/1471-2148-13-27)
Supplement: Additional file 4 — Results from simple linear regression analyses on the ‘extended dataset’ using phylogenetic generalized least squares (PGLS) model and phylogenetic independent contrasts (PIC). PIC analyses are conducted at both all-anuran and family levels; outliers, which deviated from the majority of the data points by more than three interquartile ranges from the quartiles, were excluded. All species with relevant data are included in each pairwise correlation analysis. Only correlations with degrees of freedom ≥ 3 are presented. P < 0.10 are in bold. * Dicro = Dicroglossidae; the family has ‘mean’ data only. [file 1471-2148-13-27-S4.docx]

**Additional file 4: Results from simple linear regression analyses on the ‘extended dataset’ using phylogenetic generalized least squares (PGLS) model and phylogenetic independent contrasts (PIC).** PIC analyses are conducted at both all-anuran and family levels; outliers, which deviated from the majority of the data points by more than three interquartile ranges from the quartiles, were excluded. All species with relevant data are included in each pairwise correlation analysis. Only correlations with degrees of freedom ≥ 3 are presented. P < 0.10 are in bold. * Dicro= Dicroglossidae; the family has ‘mean’ data only.

| **Models** | **PGLS** | | **PIC** | | | | | | | | | | | | |
| --- | --- | --- | --- | --- | --- | --- | --- | --- | --- | --- | --- | --- | --- | --- | --- |
|  | **All anurans** | | **All anurans** | | **Bufonidae** | | **Dendrobatoidea** | | **Dicro*** | **Hylidae** | | **Megophryidae** | | **Ranidae** | |
|  | **Range +mean** | **Mean** | **Range +mean** | **Mean** | **Range+mean** | **Mean** | **Range +mean** | **Mean** | **Mean** | **Range +mean** | **Mean** | **Range +mean** | **Mean** | **Range +mean** | **Mean** |
| FBS  ~  ES  +  CS | λ= 0.966  R^2^=0.36  df=2, 249  ES: **p<0.01**  Slope= 0.46  t=8.06  CS: **p<0.01**  Slope= 0.15  t=10.24 | λ= 0.970  R^2^=0.41  df=2, 196  ES: **p<0.01**  Slope= 0.49  t=7.92  CS: **p<0.01**  Slope= 0.16  t=9.96 | df=2, 246  R^2^=0.39  ES: **p<0.01**  Slope= 0.36  t=7.17  CS: **p<0.01**  Slope= 0.14  t=10.50  Exclude 3 outliers | df=2, 194  R^2^=0.44  ES: **p<0.01**  Slope= 0.37  t=6.98  CS: **p<0.01**  Slope= 0.15  t=10.53  Exclude 2 outliers | df=1, 16  R^2^=0.34  CS: **p=0.01**  Slope= 0.17  t=2.84 | df=1, 12  R^2^=0.28  CS: **p=0.05**  Slope= 0.13  t=2.18 | df=2, 25  R^2^=0.33  CS: **p<0.01**  Slope= 0.27  t=3.53 | df=2, 21  R^2^=0.39  CS: **p<0.01**  Slope= 0.28  t=3.64 | df=2, 19  R^2^=0.38  ES: **p<0.01**  Slope= 0.69  t=3.37 | df=2, 73  R^2^=0.27  ES: **p=0.04**  Slope= 0.20  t=2.11  CS: **p<0.01**  Slope= 0.11  t=4.75 | df=2, 48  R^2^=0.39  ES: **p<0.01**  Slope= 0.35  t=3.32  CS: **p<0.01**  Slope= 0.17  t=5.19 | df=2, 15  R^2^=0.70  ES: **p<0.01**  Slope= 0.69  t=3.80  CS: **p<0.01**  Slope= 0.24  t=5.10 | df=2, 14  R^2^=0.73  ES: **p<0.01**  Slope= 0.78  t=4.11  CS: **p<0.01**  Slope= 0.24  t=5.30 | df=2, 49  R^2^=0.44  ES: **p<0.01**  Slope= 0.47  t=2.58  CS: **p<0.01**  Slope= 0.15  t=5.57  Exclude 1 outlier | df=2, 48  R^2^=0.45  ES: **p<0.01**  Slope= 0.47  t=2.59  CS: **p<0.01**  Slope= 0.15  t=5.54  Exclude 1 outlier |
| SDI  ~  ES  +  CS | λ=0.849  df=249  ES: p=0.64  CS: p=0.38 | λ=0.895  df=255  R^2^=0.02  ES: **p=0.05**  Slope=  -0.15  t=-1.99 | df=1, 291  R^2^=0.01  CS: **p=0.07**  Slope= 0.03  t=1.81  Exclude 1 outlier | df=1, 194  R^2^=0.01  ES: p=0.18  CS: p=0.75  Exclude 2 outliers | df=1, 16  R^2^=0.18  CS: **p=0.08**  Slope= 0.18  t=1.84 | df=1, 12  R^2^=0.31  CS: **p=0.04**  Slope= 0.22  t=2.34 | df=1, 25  R^2^=0.24  CS: **p=0.01**  Slope= 0.08  t=2.78 | df=1, 21  R^2^=0.24  CS: **p=0.02**  Slope= 0.07  t=2.56 | df=2, 9  ES: p=0.45  CS: p=0.41 | df=2, 73  ES: p=0.65  CS: p=0.35 | df=2, 48  ES: p=0.58  CS: p=0.56 | df=2, 15  ES: p=0.27  CS: p=0.13 | df=2, 14  ES: p=0.29  CS: p=0.15 | df=2, 49  ES: p=0.44  CS: p=0.81  Exclude 1 outlier | df=2, 48  ES: p=0.45  CS: p=0.81  Exclude 1 outlier |
| FBS  ~  FC | λ=0.985  df=457  t=1.43  p=0.15 | λ=0.974  df=314  t=1.52  p=0.12 | df=8  t=1.18  p=0.27 | df=7  t=1.54  p=0.17 | --- | --- | df=6  t=1.41  p=0.21 | df=6  t=1.42  p=0.21 | --- | --- | --- | --- | --- | --- | --- |
| SDI  ~  FC | λ=0.822  df=457  t=-0.46  p=0.65 | λ=0.829  df=314  t=-0.36  p=0.72 | df=8  t=-1.35  p=0.22 | df=7  t=-0.55  p=0.60 | --- | --- | df=6  t=-0.33  p=0.75 | df=6  t=0.09  p=0.93 | --- | --- | --- | --- | --- | --- | --- |
| MBS  ~  MC | λ=0.982  df=457  t=0.68  p=0.50 | λ=0.982  df=314  t=0.87  p=0.38 | df=73  t=0.66  p=0.51 | df=51  t=0.29  p=0.78 | df=7  t=2.05  **p=0.08** | df=5  t=2.35  **p=0.07** | df=10  t=2.16  **p=0.06** | df=8  t=2.51  **p=0.04** | --- | df=19  t=0.81  p=0.43 | df=10  t=0.73  p=0.48 | --- | --- | df=10  t=-1.08  p=0.30 | df=9  t=-1.24  p=0.25 |
| SDI  ~  MC | λ=0.815  df=457  t=-1.00  p=0.32 | λ=0.826  df=314  t=-0.47  p=0.64 | df=73  t=0.46  p=0.65 | df=51  t=0.65  p=0.52 | df=7  t=1.01  p=0.35 | df=5  t=0.48  p=0.65 | df=10  t=-0.91  p=0.38 | df=8  t=-0.82  p=0.44 | --- | df=19  t=-0.60  p=0.56 | df=10  t=-1.72  p=0.12 | --- | --- | df=10  t=0.79  p=0.45 | df=9  t=0.84  p=0.42 |
| MBS  ~  MSC | λ=0.982  df=453  t=0.43  p=0.67 | λ=0.982  df=312  t=0.42  p=0.68 | df=34  t=0.15  p=0.88 | df=29  t=0.19  p=0.85 | df=5  t=1.54  p=0.18 | df=5  t=2.35  **p=0.07** | df=6  t=2.21  **p=0.07** | df=6  t=2.37  **p=0.06** | --- | df=5  t=0.01  p=1.00 | df=3  t=0.42  p=0.71 | --- | --- | df=9  t=-1.24  p=0.25 | df=8  t=-1.59  p=0.15 |
| SDI  ~  MSC | λ=0.819  df=453  t=0.66  p=0.51 | λ=0.825  df=312  t=0.75  p=0.45 | df=34  t=1.49  p=0.15 | df=29  t=1.17  p=0.25 | df=5  t=0.71  p=0.51 | df=5  t=0.48  p=0.65 | df=6  t=-2.26  **p=0.06** | df=6  t=-2.08  **p=0.08** | --- | df=5  t=1.09  p=0.36 | df=3  t=-1.56  p=0.22 | --- | --- | df=9  t=0.90  p=0.39 | df=8  t=0.97  p=0.36 |
| MBS  ~  MTD | λ=0.971  df=453  t=0.95  p=0.34 | λ=0.971  df=312  t=0.98  p=0.33 | df=49  t=1.02  p=0.31 | df=30  t=0.65  p=0.52 | --- | --- | df=10  t=0.53  p=0.61 | df=7  t=0.61  p=0.56 | --- | df=15  t=1.23  p=0.24 | df=8  t=0.98  p=0.36 | --- | --- |  |  |
| SDI  ~  MTD | λ=0.805  df=453  t=-1.71  **p=0.09** | λ=0.811  df=312  t=-1.49  p=0.14 | df=49  t=-1.42  p=0.16 | df=30  t=-1.03  p=0.31 | --- | --- | df=10  t=-0.32  p=0.76 | df=7  t=-0.35  p=0.74 | --- | df=15  t=-1.05  p=0.31 | df=8  t=-1.72  p=0.12 | --- | --- | --- | --- |
| SDI  ~  PC | λ=0.902  df=407  t=-3.21  **p<0.01** | λ=0.899  df=295  t=-2.59  **p=0.01** | df=38  t=-2.20  **p=0.03**  Exclude 1 outlier | df=27  t=-1.50  p=0.14  Exclude 1 outlier | --- | --- | --- | --- | --- | df=9  t=-2.41  **p=0.04** | df=5  t=-0.80  p=0.46 | --- | --- | --- | --- |
| FBS  ~  FPC | λ=0.990  df=402  t=-0.52  p=0.60 | λ=0.990  df=291  t=0.15  p=0.88 | df=28  t=-0.13  p=0.90 | df=19  t=0.49  p=0.63 | --- | --- | df=4  t=-0.21  **p<0.05** | --- | --- | df=6  t=-0.74  p=0.49 | df=3  t=0.20  p=0.85 | --- | --- | --- | --- |
| SDI  ~  FPC | λ=0.910  df=402  t=-1.28  p=0.20 | λ=0.908  df=291  t=-0.47  p=0.64 | df=28  t=-1.17  p=0.25 | df=19  t=-0.12  p=0.91 | --- | --- | df=4  t=-0.47  p=0.66 | --- | --- | df=6  t=-2.44  **p<0.05** | df=3  t=0.24  p=0.83 | --- | --- | --- | --- |
| MBS  ~  MPC | λ=0.984  df=402  t=-0.22  p=0.83 | λ=0.976  df=291  t=0.46  p=0.65 | df=27  t=0.07  p=0.95 | df=20  t=0.04  p=0.97 | --- | --- | --- | --- | --- | --- | --- | --- | --- | --- | --- |
| SDI  ~  MPC | λ=0.901  df=402  t=-2.86  **p<0.01** | λ=0.894  df=291  t=-2.84  **p<0.01** | df=27  t=-2.03  **p=0.05** | df=20  t=-1.90  **p=0.07** | --- | --- | --- | --- | --- | --- | --- | --- | --- | --- | --- |
| ES  ~  PC | λ=0.964  df=409  t=1.37  p=0.17 | --- | df=47  t=0.91  p=0.37 |  | --- | --- | --- | --- | --- | df=8  t=-0.16  p=0.88 | --- | --- | --- | --- | --- |
| CS  ~  PC | λ=0.964  df=371  t=-2.84  **p<0.01** | --- | df=49  t=-2.32  **p=0.02** | --- | --- | --- | --- | --- | --- | df=9  t=-1.70  p=0.12 | --- | --- | --- | --- | --- |
| ES  ~  FPC | λ=0.966  df=404  t=-0.64  p=0.52 | --- | df=23  t=-0.50  p=0.62 | --- | --- | --- | df=3  t=-1.55  p=0.22 | --- | --- | df=4  t=-0.62  p=0.57 | --- | --- | --- | --- | --- |
| CS  ~  FPC | λ=0.961  df=366  t=-2.87  **p<0.01** | --- | df=28  t=-2.60  **p=0.01** | --- | --- | --- | df=5  t=-1.32  p=0.24 | --- | --- | df=6  t=-2.08  **p=0.08** | --- | --- | --- | --- | --- |
| ES  ~  MPC | λ=0.963  df=404  t=1.74  **p=0.08** | --- | df=35  t=0.07  **p=0.09** | --- | --- | --- | --- | --- | --- | --- | --- | --- | --- | --- | --- |
| CS  ~  MPC | λ=0.961  df=366  t=-0.59  p=0.55 | --- | df=31  t=-0.54  p=0.60 | --- | --- | --- | --- | --- | --- | --- | --- | --- | --- | --- | --- |

SDI=sexual size dimorphism index; CS=log clutch size; ES=log egg size; MBS=log male body size; FBS=log female body size; FC=female combat; MC=male combat; MSC=male scramble competition; MTD=male territory defence; PC=parental care; FPC=female parental care; MPC=male parental care.
